# A linker histone acts as a transcription factor to orchestrate malic acid accumulation in apple in response to sorbitol

**DOI:** 10.1093/plcell/koae328

**Published:** 2024-12-20

**Authors:** Da-Gang Hu, Mengxia Zhang, Chunlong Li, Ting-Ting Zhao, Lian-Da Du, Quan Sun, Chu-Kun Wang, Dong Meng, Cui-Hui Sun, Zhangjun Fei, Abhaya M Dandekar, Lailiang Cheng

**Affiliations:** Section of Horticulture, School of Integrative Plant Science, Cornell University, Ithaca, NY 14853, USA; National Research Center for Apple Engineering and Technology, Shandong Collaborative Innovation Center for Fruit and Vegetable Quality and Efficient Production, College of Horticulture Science and Engineering, Shandong Agricultural University, Tai’an, Shandong 271018, China; Section of Horticulture, School of Integrative Plant Science, Cornell University, Ithaca, NY 14853, USA; Section of Horticulture, School of Integrative Plant Science, Cornell University, Ithaca, NY 14853, USA; National Research Center for Apple Engineering and Technology, Shandong Collaborative Innovation Center for Fruit and Vegetable Quality and Efficient Production, College of Horticulture Science and Engineering, Shandong Agricultural University, Tai’an, Shandong 271018, China; National Research Center for Apple Engineering and Technology, Shandong Collaborative Innovation Center for Fruit and Vegetable Quality and Efficient Production, College of Horticulture Science and Engineering, Shandong Agricultural University, Tai’an, Shandong 271018, China; National Research Center for Apple Engineering and Technology, Shandong Collaborative Innovation Center for Fruit and Vegetable Quality and Efficient Production, College of Horticulture Science and Engineering, Shandong Agricultural University, Tai’an, Shandong 271018, China; Section of Horticulture, School of Integrative Plant Science, Cornell University, Ithaca, NY 14853, USA; National Research Center for Apple Engineering and Technology, Shandong Collaborative Innovation Center for Fruit and Vegetable Quality and Efficient Production, College of Horticulture Science and Engineering, Shandong Agricultural University, Tai’an, Shandong 271018, China; Section of Horticulture, School of Integrative Plant Science, Cornell University, Ithaca, NY 14853, USA; Section of Horticulture, School of Integrative Plant Science, Cornell University, Ithaca, NY 14853, USA; National Research Center for Apple Engineering and Technology, Shandong Collaborative Innovation Center for Fruit and Vegetable Quality and Efficient Production, College of Horticulture Science and Engineering, Shandong Agricultural University, Tai’an, Shandong 271018, China; Boyce Thompson Institute, Ithaca, NY 14853, USA; Department of Plant Sciences, University of California at Davis, Davis, CA 95616, USA; Section of Horticulture, School of Integrative Plant Science, Cornell University, Ithaca, NY 14853, USA

## Abstract

High carbohydrate availability promotes malic acid accumulation in fleshy fruits, but the underlying mechanism is not known. Here, we show that antisense repression of *ALDOSE-6-PHOSPHATE REDUCTASE* in apple (*Malus domestica*) decreases the concentrations of sorbitol and malate and the transcript levels of several genes involved in vacuolar malate transport, including the aluminum-activated malate transporter (ALMT) gene *MdALMT9* (*Ma1*), the P-ATPase gene *MdPH5*, the MYB transcription factor gene *MdMYB73*, and the cold-induced basic helix-loop-helix transcription factor gene *MdCIbHLH1*, in fruit and leaves. We identified a linker histone H1 variant, MdH1.1, which complements the Arabidopsis (*Arabidopsis thaliana*) H1 deficient mutant and functions as a transcription factor. MdH1.1 activates *MdMYB73*, *MdCIbHLH1*, and *MdPH5* expression by directly binding to their promoters. MdMYB73, in return, binds to the promoter of *MdH1.1* to enhance its transcription. This MdH1.1-MdMYB73 feedback loop responds to sorbitol, regulating *Ma1* expression. Antisense suppression of either *MdH1.1* or *MdMYB73* expression significantly decreases whereas overexpression increases *Ma1* expression and malate accumulation. These findings demonstrate that MdH1.1, in addition to being an architectural protein for chromatin structure, operates as a transcription factor orchestrating malic acid accumulation in response to sorbitol, revealing how sugar signaling modulates vacuolar malate transport via a linker histone in plants.

## Teaser

Sorbitol modulates malic acid accumulation in apple by enhancing the expression of a linker histone that acts as a transcription factor for promoting the expression of a tonoplast malate transporter.

## Introduction

Malate is a key metabolite in the glycolysis and tricarboxylic acid (TCA) cycle and the glyoxylate cycle as well as crassulacean acid metabolism and C4 photosynthesis; its accumulation in the vacuole allows for carbon and energy storage, osmotic adjustment, and cytosolic pH homeostasis in response to developmental cues and abiotic stress whereas cellular export of malate from roots enables acidification of the rhizosphere for aluminum detoxification and nutrient uptake (Fernie and Martinoia 2009; Finkemeier and Sweetlove 2009; Hu et al. 2016a; Kamran et al. 2020). In many fleshy fruits, malate is the predominant organic acid and its accumulation in the vacuole largely determines fruit acidity and the perception of sweetness (Etienne et al. 2013; Butelli et al. 2019; Wege 2020), 2 key components of taste and flavor (Colaric et al. 2005; Bugaud et al. 2011). Fruit malate level is genetically controlled (Ye et al. 2017; Li et al. 2020a), but is also affected by source-sink relationship and environmental factors (Etienne et al. 2013). A high source to sink ratio enhances the accumulation of malic acid as well as soluble sugars in apple (*Malus domestica*) (Serra et al. 2016; Anthony et al. 2019), peach (*Prunus persica*) (Souty et al. 1999), and grape (*Vitis vinifera*) (Kliewer and Dokoozlian 2005). However, the molecular mechanism linking fruit acidity to carbohydrate availability is not known. High carbohydrate availability could trigger sugar signaling, regulating vacuolar malate accumulation.

Although malate metabolism can alter fruit acidity (Sweetman et al. 2009; Centeno et al. 2011; Gao et al. 2024), intracellular malate transport from the cytosol into the vacuole is largely responsible for natural variations in fruit acidity (Berüter 2004; Hu et al. 2017; Ye et al. 2017; Li et al. 2020a). The transport of malate across the tonoplast occurs by facilitated diffusion (Etienne et al. 2013), a process mediated by 2 members of the aluminum-activated malate transporter (ALMT) family, ALMT9 and ALMT6 (Kovermann et al. 2007; Meyer et al. 2011), as well as tonoplast dicarboxylate transporter (tDT) (Emmerlich et al. 2003). *ALMT9* underlies a major malic acid (*Ma*) locus, in apple (Bai et al. 2012, 2015; Khan et al. 2013; Li et al. 2020a) and the major quantitative trait locus responsible for fruit acidity in tomato (Ye et al. 2017). To drive the facilitated diffusion of malate, vacuolar-type H^+^-ATPase (V-ATPase, VHA), H^+^- pyrophosphatase (V-PPase, VHP), and P-type ATPase (P-ATPase such as PH1, PH5, etc.) pump protons into the vacuole, lowering its pH (Etienne et al. 2013; Martinoia 2018). Upon entry into the acidic vacuole, the dianion malate gets protonated instantly, which traps malate in the acid form to effectively maintain its concentration gradient across the tonoplast for continuous diffusion into the vacuole (Martinoia et al. 2007; Etienne et al. 2013; Martinoia 2018). Both tonoplast malate transporters and vacuolar proton pumps are transcriptionally regulated. A WRKY transcriptional repressor, SlWRKY42, directly binds to the promoter of *SlALMT9*, negatively regulating malate accumulation in tomato fruit (Ye et al. 2017). In apple, an MYB transcription factor, MdMYB73 interacts with a cold-induced basic helix-loop-helix (bHLH) transcription factor, MdCIbHLH1, enhancing vacuolar acidification and malate accumulation by activating the expression of *MdALMT9* (*Ma1*), *MdVHA-A* (V-ATPase subunit A), and *MdVHP1* (V-PPase 1) (Hu et al. 2017). A BTB-TAZ domain protein, MdBT2 ubiquitinates both MdMYB73 and MdCIbHLH1 for their degradation, lowering malate accumulation in response to nitrate (Zhang et al. 2020a, 2020b). MdMYB1/10 interacts with MdbHLH3, regulating the expression of several genes encoding vacuolar proton pump subunits, including *MdVHA-Bs*, *MdVHA-E2* and *MdVHP1*, and *MdtDT*, in promoting vacuolar acidification and malate accumulation in response to low temperature (Xie et al. 2012; Hu et al. 2016b). In addition, the expression of *Ma1* is activated by MdMYB123 but repressed by MdMYB44 and MdMYB21 (Jia et al. 2021; Peng et al. 2023; Zheng et al. 2023), and *Ma1* is regulated at the post-transcriptional level via alternative splicing (Li et al. 2024). If sugar signaling is involved in regulating malate accumulation, it likely targets some of the players in this molecular network.

Linker histones (H1s) are chromatin-associated proteins that play essential roles in stabilizing nucleosomes and compacting them into higher order chromatin structures. All H1s across eukaryotes have a structured globular domain with a winged helix-turn-helix DNA-binding motif and intrinsically disordered, flexible N and C-terminal regions (Hergeth and Schneider 2015; Hao et al. 2021). There are 3 canonical H1 variants in Arabidopsis (Wierzbicki and Jerzmanowski 2005; Kotliński et al. 2017). Triple T-DNA mutant *h1.1h1.2h1.3* (*3h1*) exhibits de-regulation of developmental transitions including seed dormancy, flowering time, lateral root number, root hair density, and stomatal patterning (Rutowicz et al. 2019). The distribution of H1 is not uniform across the Arabidopsis genome, and the level of gene expression is inversely correlated with nucleosome density (Li et al. 2014; Rutowicz et al. 2019). The interactions of H1 with DNAs (core nucleosome DNAs and linker DNAs) and other proteins in the chromatin organization modulate gene expression in several ways. First, it restricts the access of RNA polymerase II and transcription factors to the promoter of a gene (Hergeth and Schneider 2015). Second, it enhances the epigenetic silencing of genes via both DNA methylation and core histone H3 methylation (Wierzbicki and Jerzmanowski 2005; Yang et al. 2013). In Arabidopsis, H1 prevents non-CG methylation-mediated small RNA biogenesis and loss of H1 leads to a higher level of small RNA-directed DNA methylation in heterochromatin (Choi et al. 2021). Third, it interacts with transcription activators/repressors, altering their activity (Hergeth and Schneider 2015; Soler et al. 2017). Finally, it was shown in mouse embryonic stem cells that H1 prevents noncoding RNA transcription and regulates noncoding transcript turnover on chromatin (Fernández-Justel et al. 2022). However, despite H1s being DNA-binding proteins, it is not known if any of the H1s functions as a transcription factor, directly regulating gene expression.

Sugar alcohols are end products of photosynthesis and transport carbohydrates in many plant species, contributing about 30% to the global primary carbon production (Bieleski 1982). In apple and many other tree fruits in the Rosaceae family, sorbitol is the predominant photosynthate produced in the leaves and transported in the phloem (Zimmermann and Ziegler 1975; Bieleski and Redgwell 1985; Moing et al. 1992; Cheng et al. 2005). In source leaves, sorbitol is synthesized from glucose-6-phosphate (G6P) via a 2-step process in the cytosol: conversion of G6P to sorbitol-6-phosphate (S6P) by aldose-6-phosphate reductase (A6PR), and dephosphorylation of S6P to sorbitol by S6P phosphatase (Negm and Loescher 1981; Zhou et al. 2003a, 2003b). After entering the phloem via diffusion through plasmodesmata (Reidel et al. 2009; Fu et al. 2011), sorbitol is transported to sink tissues where it is primarily converted to fructose by sorbitol dehydrogenase (Negm and Loescher 1979; Yamaguchi et al. 1994). In addition to being a carbon and energy source, sorbitol acts as a signal regulating carbohydrate metabolism in both fruit and shoot tips (Archbold 1999; Zhou et al. 2006; Meng et al. 2023), flower development and pollen tube growth (Meng et al. 2018a; Li et al. 2020b), and resistance to a fungal pathogen, *Alternaria alternata* (Meng et al. 2018b). In transgenic apple trees where sorbitol synthesis is decreased by antisense repression of *A6PR* expression (Cheng et al. 2005), more sucrose is transported to sink tissues and the subsequent upregulation of sucrose metabolism largely maintains tree vegetative growth and fruiting (Zhou et al. 2006; Li et al. 2018). However, reduced malate levels were detected in the fruit and leaves of these antisense *A6PR* trees (Teo et al. 2006; Ma 2012). This has led us to postulate that sorbitol may modulate malate accumulation in apple. In this work, we report the identification and characterization of a linker histone H1 variant, MdH1.1, which functions as a transcription factor that forms a positive feedback loop with MdMYB73 to orchestrate sorbitol-modulated malate accumulation in apple.

## Results

### Decreased sorbitol synthesis leads to reduced malate accumulation in antisense *A6PR* apple fruit and leaves

**Figure 1.**
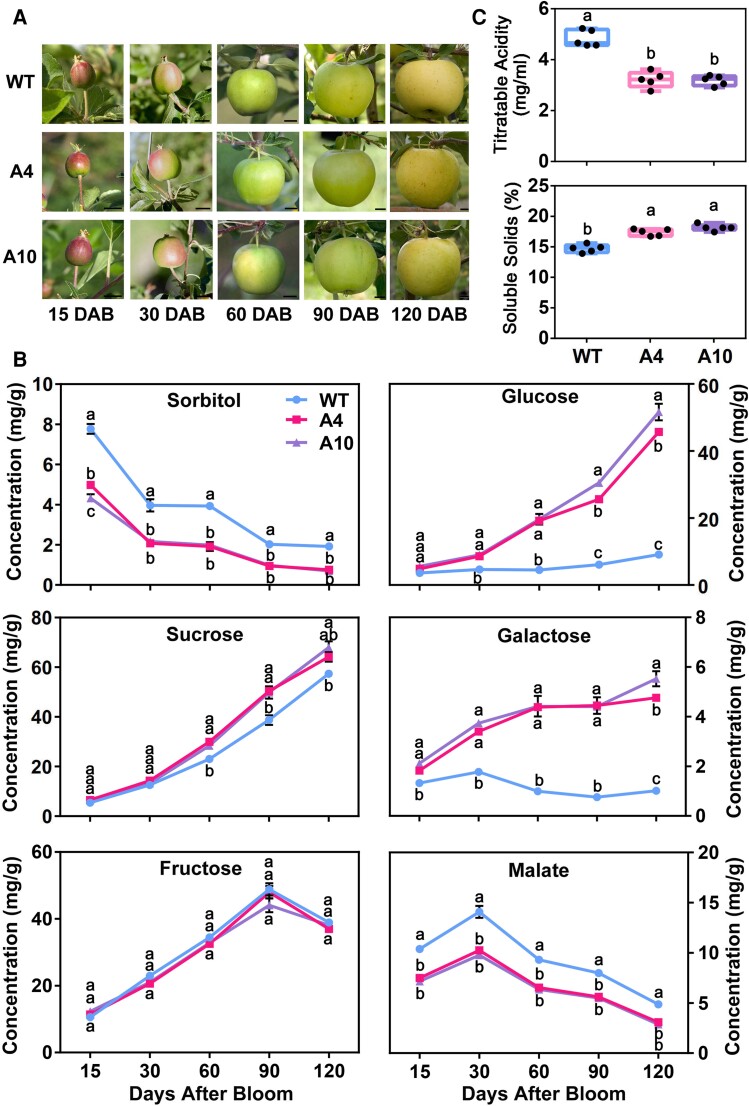
Decreased sorbitol synthesis leads to reduced malate accumulation in the fruit of *ALDOSE-6-PHOSPHATE REDUCTASE (A6PR)* antisense lines of “Greensleeves” apple. **A)** Photos of fruits from the 2 antisense lines A4 and A10 and the WT control, collected at 15, 30, 60, 90, and 120 days after bloom (DAB). Scale bars = 2 cm. **B)** Concentrations of sorbitol, sucrose, fructose, glucose, galactose, and malate in fruit as shown in **A)**. Data are mean ± SE of 5 biological replicates with 4 to 6 fruits each from a single-tree replicate. **C)** Titratable acid and soluble solids content in the fruit at harvest (120 DAB) of the 2 antisense lines A4 and A10, and WT control. Data are obtained from 5 biological replicates with 6 fruits each from a single-tree replicate. The boxes represent interquartile ranges, with the middle lines as medians and the whiskers as the maximum and minimum values. In **B)** and **C)**, different letters indicate significant difference using Tukey's HSD test at *P* < 0.05 after one-way ANOVA.

In earlier work, we found that antisense repression of *A6PR*, the gene encoding the key enzyme for sorbitol synthesis, decreased sorbitol synthesis in leaves and sorbitol concentrations in both leaves and fruit, and the fruit had lower malate levels at harvest (Cheng et al. 2005; Teo et al. 2006; Li et al. 2018). To fully characterize the developmental profiles of fruit sugars and malate in response to *A6PR* suppression, we collected fruits from 2 antisense *A6PR* lines (A4 and A10) and wild-type (WT) control at 5 key developmental stages, i.e. 15 (active cell division), 30 (end of cell division and beginning of cell expansion), 60 (early rapid cell expansion), 90 (late rapid cell expansion), and 120 (maturity) days after bloom (DAB) for metabolite analysis via gas chromatography/mass spectrometry (GC/MS) (Fig. 1A). Compared with WT, the 2 antisense lines, A4 and A10, had lower sorbitol levels, similar fructose levels, but higher concentrations of glucose and galactose throughout fruit development; sucrose concentration was not significantly different between the 2 antisense lines and WT at the early stages of fruit development, but was slightly higher later during fruit development in the 2 antisense lines (Fig. 1B). Fruit malate levels peaked at 30 DAB and then declined to fruit harvest in all 3 genotypes, but the transgenic fruit had a lower malate level at each developmental stage (Fig. 1B). At fruit harvest, the transgenic fruit had significantly lower titratable acidity (about 2/3 of the WT fruit) but significantly higher soluble solids (Fig. 1C).

To compare sugar and malate profiles between fruit and leaves, we measured the concentrations of soluble sugars and malate in fully expanded leaves of the 2 antisense lines (A4 and A10) and WT via GC/MS (Supplementary Fig. S1). Antisense suppression of *A6PR* significantly decreased sorbitol concentrations but increased sucrose concentrations in leaves, with those of fructose, glucose, and galactose unaltered. Leaf malate levels in both transgenic lines (A4 and A10) were also significantly lower than in WT control (Supplementary Fig. S1). Among the sugars that differed in concentrations between the antisense lines and WT, glucose, and galactose showed contrasting responses to antisense repression of *A6PR* between fruit and leaves (Fig. 1B; Supplementary Fig. S1); sucrose concentration was negatively associated with malate levels in leaves, but no association or a slightly negative association was detected at the early stages of fruit development (15 DAB and 30 DAB) vs. at later stages (Fig. 1B; Supplementary Fig. S1). Sorbitol appears to be the only sugar that was positively associated with malate levels in both fruit and leaves, suggesting it may modulate malate accumulation in apple.

### Identification of candidate genes involved in malate transport via genome-wide RNA sequencing and Reverse transcription quantitative PCR in fruit and leaves of the WT and antisense *A6PR* lines

To identify genes involved in the reduction of malate accumulation in the transgenic fruit, we extracted total RNA from the fruits of WT and antisense *A6PR* line A4 at 15, 60, and 120 DAB, and conducted genome-wide RNA sequencing (RNA-seq) analysis, with 5 replicates each. After removing adapters, rRNAs and low-quality reads, we obtained a total of 445.5 million high quality reads, ∼85% of which were mapped to the “Golden Delicious” doubled haploid reference genome, GDDH13 (Supplementary Data Set 1). After identifying differentially expressed genes (DEGs), we first focused on the genes associated with malate metabolism and transport and found that the expression of *ALMT9* (*Ma1*), *tDT*, and others involved in malate transport were decreased in A4 transgenic fruit to various degrees across the 3 developmental stages compared with the WT control (Supplementary Fig. S2A; Supplementary Data Set 1). The expression of *MYB73*, a key transcription factor for regulating *Ma1* expression (Hu et al. 2017), was also lower in A4 transgenic fruit than in WT control (Supplementary Fig. S2A; Supplementary Data Set 1).

To determine if similar patterns of gene expression are present in the leaves, we searched an RNA-seq dataset obtained previously on fully expanded leaves of the 2 antisense *A6PR* lines, A4 and A10, and WT (Wu et al. 2015) for the same group of potential candidate genes selected from the fruit RNA-seq that are associated with malate metabolism and transport. We found that the expression of *ALMT9* (*Ma1*), *tDT*, and *MdMYB73* was decreased in leaves of the 2 antisense lines compared with the WT control (Supplementary Fig. S2B; Supplementary Data Set 2). These data suggest that some of these genes may play a role in reducing malate accumulation in response to decreased sorbitol synthesis.

**Figure 2.**
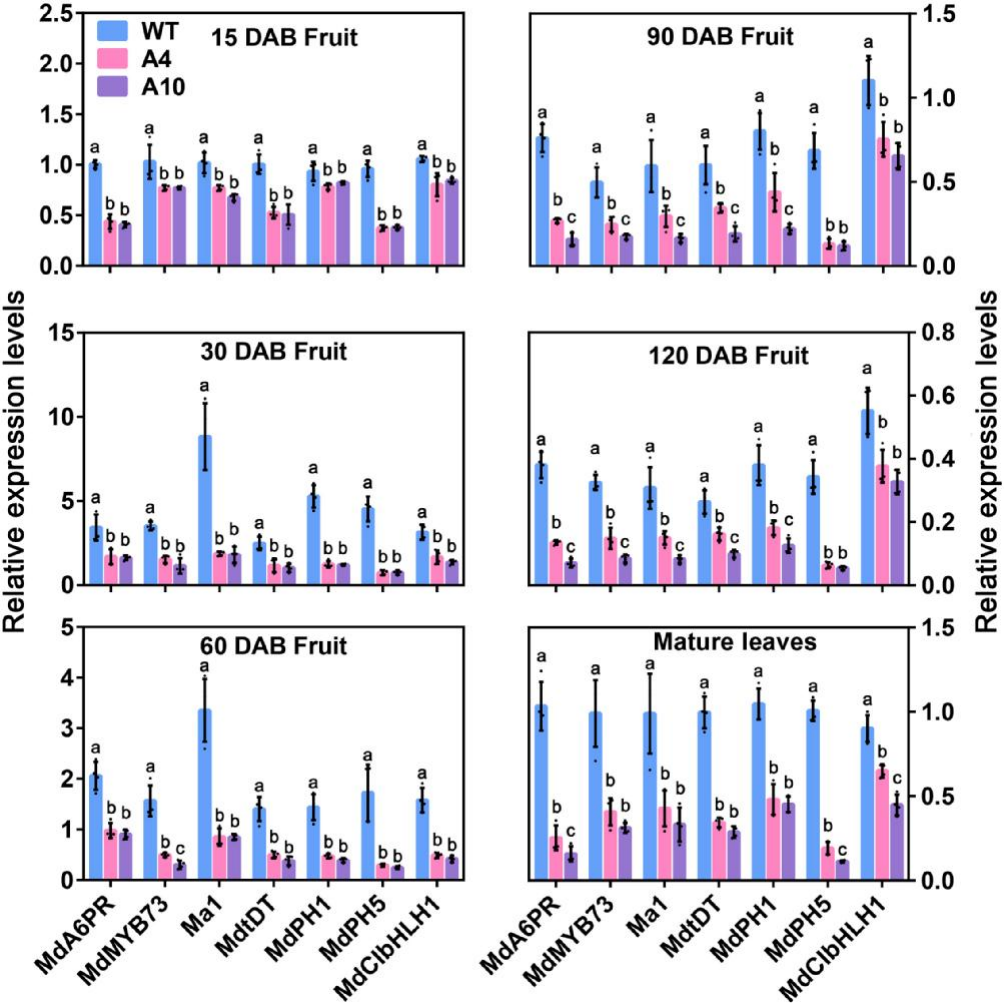
Relative expression levels of potential candidate genes involved in malate accumulation in the fruit and fully expanded leaves of the control (WT) and *A6PR* antisense lines (A4 and A10) of “greensleeves” apple. Relative expression levels of genes encoding the MYB domain protein 73 (*MdMYB73*), aluminum-activated malate transporter 9 (*MdALMT9*/*Ma1*), tonoplast dicarboxylate transporter (*MdtDT*), P-type H^+^-ATPases (*MdPH1* and *MdPH5*), and bHLH transcription factor *MdCIbHLH1* in the fruit and mature leaves of the WT and 2 *A6PR* antisense lines A4 and A10 at fruit developmental stages of 15, 30, 60, 90, and 120 DAB. The relative expression level of each gene was obtained using the ddCT method, with *ACTIN* as a reference. Data are mean ± SE of 4 biological replicates with 4 to 6 fruits from a single-tree replicate or 4 leaves pooled from 2 trees per replicate. Different letters indicate significant difference using Tukey's HSD test at *P* < 0.05 after ANOVA.

To ascertain the expression patterns of potential candidate genes associated with malate accumulation, we performed reverse transcription quantitative PCR (RT-qPCR) analysis on both fruit and leaves. The 2 antisense *A6PR* lines A4 and A10 had significantly lower expression levels of malate transporters, *Ma1* and *MdtDT*, P-type *H^+^-ATPases*, *MdPH1*, and *MdPH5*, and transcription factors, *MdMYB73,* and *MdCIbHLH1*, involved in malate transport and vacuolar acidification in the fruit throughout fruit development and in fully expanded leaves (Fig. 2). These data strongly suggest that reduced malate accumulation in the antisense lines is associated with the expression of these potential candidate genes.

### The expression of *MdH1.1*, *MdMYB73*, and *Ma1* responds specifically to sorbitol

**Figure 3.**
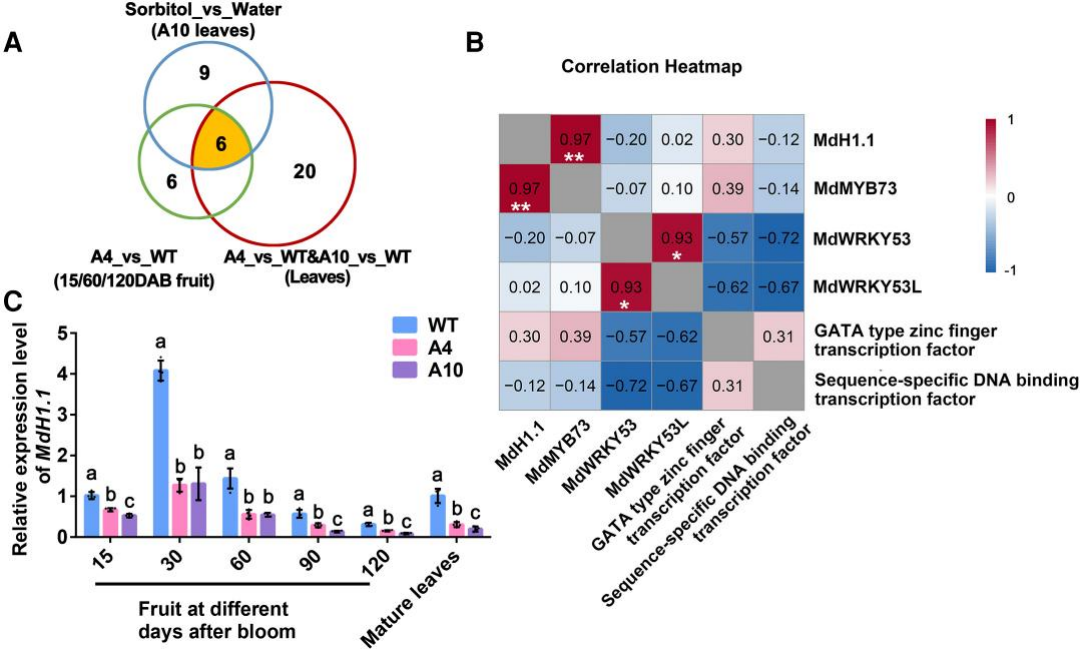
Three candidate genes encoding linker histone (*MdH1.1*), MYB domain protein 73 (*MdMYB73*), and aluminum-activated malate transporter 9 (*MdALMT9*/Ma1) respond to sorbitol. **A)** Schematic of the Venn diagram analysis of the sorbitol-feeding RNA-seq dataset combined with the other 2 datasets (A4 fruit vs. WT fruit at 3 developmental stages; A4 and A10 leaves vs. WT leaves) to identify transcription factors that are overlapped in all 3 datasets and differentially expressed between 50 mm sorbitol treatment and the control (Fold change > 1.5; FDR < 0.05). **B)** Correlation heatmap of relative expression level of 6 transcription factors, *MdH1.1*, *MdMYB73*, *MdWRKY53*, *MdWRKY53L*, a GATA type zinc finger transcription factor, and a sequence-specific DNA-binding transcription factor in A4 vs. WT fruit collected at 15, 60, and 120 DAB during fruit development. Red and blue colors represent positive and negative correlations, respectively. Gray indicates the correlation of transcription factors with themselves. The *P* values for the correlations between *MdH1.1* and *MdMYB73* and between *MdWRKY53* and *MdWRKY53L* are 0.97 and 0.93, respectively. **C)** Relative expression levels of *MdH1.1* in the fruit at 15, 30, 60, 90, and 120 DAB and in mature leaves of the WT and 2 *A6PR* antisense lines A4 and A10. The relative expression level of each gene was obtained using the ddCT method, with *ACTIN* as a reference. Data are mean ± SE of 4 biological replicates with 4 to 6 fruits from a single-tree replicate or 4 leaves pooled from 2 trees per replicate. Different letters indicate significant difference using Tukey's HSD test at *P* < 0.05 after ANOVA.

To narrow down the list of candidate genes, we conducted an RNA-seq analysis on A10 leaves fed with sorbitol via the petiole based on the idea that any gene downregulated by sorbitol in the transgenic leaves would respond positively to sorbitol feeding. We used 50 mm sorbitol, with water as control (Zhou et al. 2006), replicated 5 times each. After cleaning the raw reads, we obtained a total of 130.6 million high quality reads, ∼95% of which were mapped to the GDDH13 apple reference genome. From the RNA-seq data, we found that the expression of those involved in malate metabolism and transport were significantly higher in the sorbitol feeding treatment than the water control (FDR < 0.05), with *MdMYB73* and *Ma1* upregulated 2.8-, and 1.6-fold, respectively (Supplementary Fig. S3; Supplementary Data Set 3). To identify new transcription factor(s) involved in the regulation of malate accumulation in response to sorbitol, we combined the sorbitol-feeding RNA-seq dataset with the other 2 datasets (A4 fruit vs. WT fruit at 3 developmental stages; A4 and A10 leaves vs. WT leaves) to look for transcription factors that are overlapped in all 3 datasets (Fold Change > 1.5; FDR < 0.05) (Fig. 3A). This yielded a total of 6 transcription factors: MdMYB73, a transcriptional activator for *Ma1* (Hu et al. 2017); MD12G1243700, which was annotated as a transcription factor with a winged-helix DNA-binding domain (WHD) but has homology with Arabidopsis linker histone protein H1.1 (Supplementary Figures S4 and S5; Supplementary Files 1 and 2; Kotliński et al. 2017), so was subsequently named as MdH1.1; 2 WRKY transcription factors, MdWRKY53 and MdWRKY53-Like protein (MdWRKY53L); a GATA type zinc finger transcription factor; and a sequence-specific DNA-binding transcription factor. *MdH1.1* was highly co-expressed with *MdMYB73*, whereas *MdWRKY53* was highly co-expressed with *MdWRKY53L* during fruit development in A4 and WT fruits (Fig. 3B). By using virus-induced gene silencing via the tobacco (*Nicotiana tabacum*) rattle virus (TRV) vector in combination with injection of 50 mm sorbitol into fruit cortex tissue, we found that, while the expression of all 4 genes was stimulated by sorbitol, suppression of *MdH1.1* blocked sorbitol-induced upregulation of *Ma1* expression and corresponding increase in malate levels whereas suppression of *MdWRKY53* or *MdWRKY53L* did not (Supplementary Fig. S6). The expression level of *MdH1.1* was significantly lower in both fruit and leaves of A4 and A10 relative to WT (Fig. 3C). These data indicate that MdH1.1 is involved in sorbitol-modulated malate accumulation.

**Figure 4.**
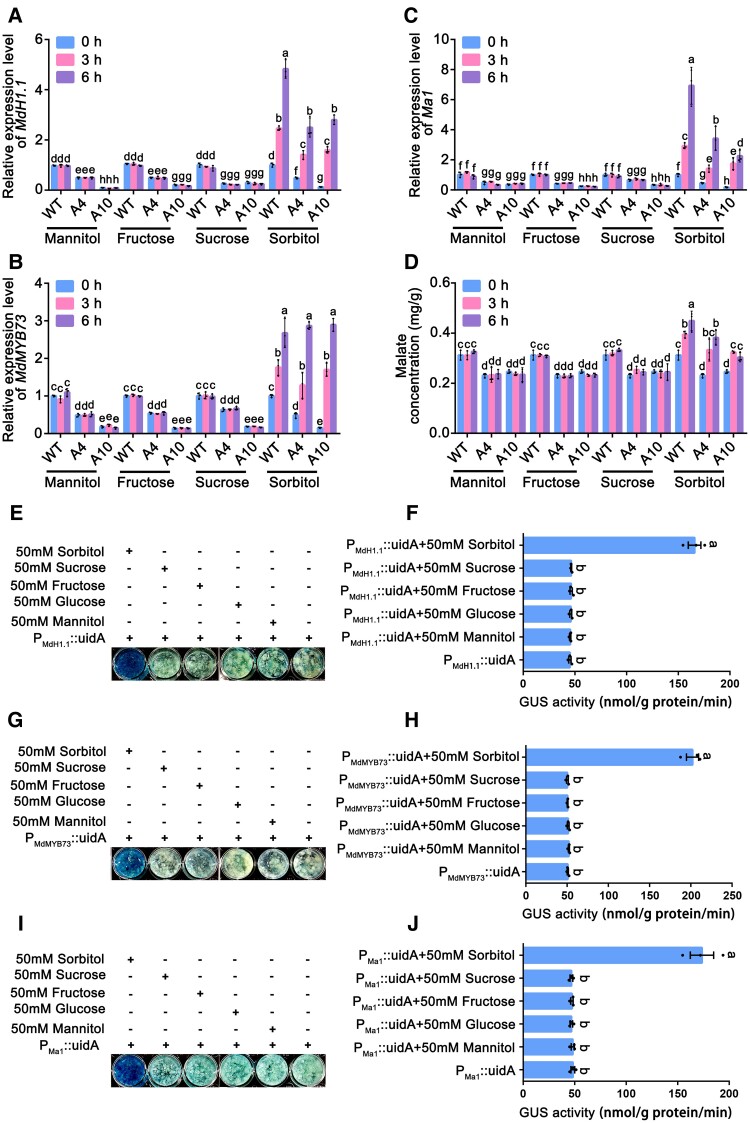
The expression levels of 3 candidate genes (*MdH1.1*, *MdMYB73*, and *Ma1*) and malate levels in the leaves of WT and *A6PR* antisense lines (A4 and A10) of the “Greensleeves” apple, along with GUS assays of the promoters of these genes, in response to sugar feeding. **A)** to **C)** Relative expression levels of *MdH1.1***A)**; *MdMYB73***B)**; and *Ma1***C)**; in the leaves of WT control and antisense lines A4 and A10 in response to feeding with 50 mm mannitol, sorbitol, fructose, or sucrose via the leaf petioles. The relative expression level of each gene was obtained using the ddCT method, with *ACTIN* as a reference. **D)** Malate concentrations in the leaves of WT control and antisense lines A4 and A10 in response to feeding with 50 mm mannitol, sorbitol, fructose, or sucrose. **E)** GUS assays of *P_MdH1.1_::uidA* transgenic apple calli on different sugars. The *P_MdH1.1_::uidA* reporter construct was introduced into “Orin” apple calli. The transgenic apple calli were provided with 50 mm sorbitol, sucrose, fructose, glucose, or mannitol for 12 h at 25 °C in the dark, and were stained to visualize GUS activity, with no sugar treatment as a control. **F)** GUS activity in *P_MdH1.1_::uidA* transgenic apple calli on different sugars as indicated in **E)**. **G)** GUS assays of *P_MdMYB73_::uidA* transgenic apple calli on different sugars with no sugar treatment as a control. **H)** GUS activity in *P_MdMYB73_::uidA* transgenic apple calli on sugars as indicated in **G)**. **I)** GUS assays of *P_Ma1_::uidA* transgenic apple calli on different sugars, with no sugar treatment as a control. **J)** GUS activity in *P_Ma1_::uidA* transgenic apple calli on different sugars as indicated in **I)**. In **A)** to **D)**, data are mean ± SE of 3 biological replicates with 3 leaves pooled from 3 shoots per replicate. Different letters indicate significant difference using Tukey's HSD test at *P* < 0.05 after ANOVA. In **F)**, **H)**, and **J)**, data are mean ± SE of 3 biological replicates with calli grown in 1 petri dish as a replicate. All experiments were repeated 3 times with similar results. Different letters indicate significant difference using Tukey's HSD test at *P* < 0.05 after ANOVA.

To confirm the response of *MdH1.1*, *MdMYB73*, and *Ma1* expression to sorbitol while excluding the possibility of being an osmotic effect, we fed leaves of 2 antisense *A6PR* lines A4 and A10 along with WT with 50 mm sorbitol and 50 mm mannitol, respectively. In addition, we included 50 mm fructose and 50 mm sucrose as treatments to determine if any gene expression response to sorbitol is specific and signaling in nature considering that sorbitol is primarily converted to fructose and transgenic leaves have higher sucrose levels (Supplementary Fig. S1). In response to the sorbitol feeding (leaf sugar levels shown in Supplementary Fig. S7), transcript levels of *MdH1.1*, *MdMYB73*, and *Ma1* and malate levels increased significantly at 3 and 6 h in all 3 genotypes (Fig. 4A to D), whereas those of the 3 genes and malate levels remained unchanged for the feeding of mannitol, fructose or sucrose (Fig. 4A to D; Supplementary Fig. S7). These findings strongly suggest that *MdH1.1*, *MdMYB73*, and *Ma1* respond specifically to sorbitol and sorbitol modulates malate accumulation via signaling.

To determine the response of fruit to exogenous sugar application, we injected the fruit of WT and 2 antisense *A6PR* lines A4 and A10 with 50 mm mannitol, sorbitol, glucose, or sucrose, and kept them at 16℃ for 7 d. In the WT fruit, sugar injection elevated the corresponding sugar level without causing any tissue browning around the injection site, with only sorbitol injection leading to significantly higher expression levels of *MdH1.1*, *MdMYB73*, and *Ma1* and higher concentrations of malate (Supplementary Fig. S8). However, the A4 and A10 fruits exhibited extensive browning around the injection site (Supplementary Fig. S8A), which prevented evaluation of any effect of the sugars on malate accumulation. The experiment was repeated 3 times with fruit surface sterilization, but the same phenotype was observed for the transgenic fruit. It appears that the A4 and A10 fruits were much more susceptible to mechanical damage than WT possibly due to altered cell wall composition and structure.

To verify the sorbitol-specific response of *MdH1.1*, *MdMYB73*, and *Ma1*, we cloned their promoters and used these promoters to drive GUS expression in apple calli, respectively, and then assayed GUS activity in response to addition of 50 mm mannitol (osmotic control) and 50 mm fructose (equimolar carbon control) as well as those showing some responses in either fruit or leaves of the antisense lines to decreased sorbitol synthesis: 50 mm sucrose, 50 mm glucose, 50 mm galactose, and 50 mm Myo-inositol (Fig. 1B; Supplementary Fig. S1; Li et al. 2018). We found that *pMdH1.1::GUS*, *pMdMYB73::GUS*, and *pMa1::GUS* transgenic calli treated with 50 mm sorbitol exhibited significantly higher GUS activities than the mannitol control whereas no difference in GUS activity was detected between any other sugar treatment and the mannitol control (Fig. 4E to J; Supplementary Fig. S9), confirming the sorbitol-specific response of these 3 genes for malate accumulation.

### Both linker histone MdH1.1 and transcription factor MdMYB73 are essential for sorbitol-modulated malate accumulation

**Figure 5.**
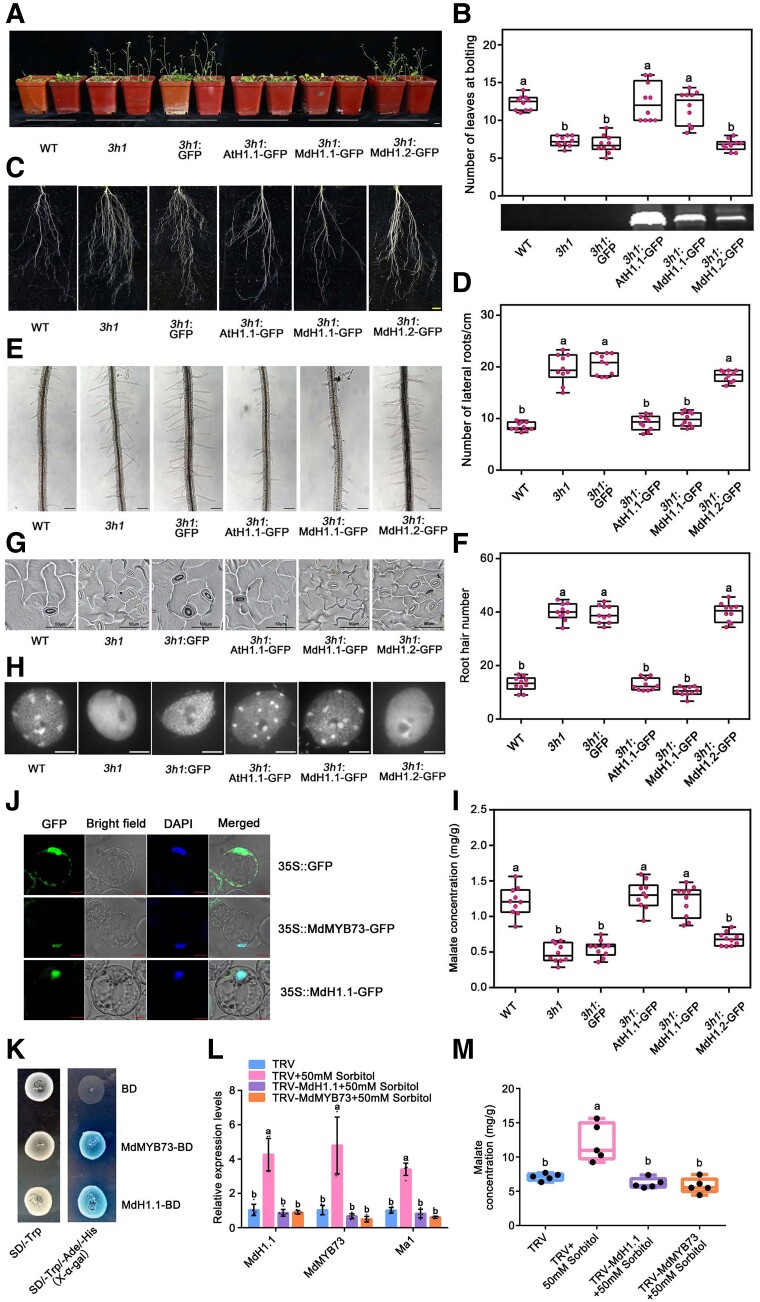
Complementation of Arabidopsis *3h1* mutant with apple linker histone H1.1 (MdH1.1) and the essential roles of *MdH1.1* and MdMYB73 in sorbitol-mediated malate accumulation. **A)** Flowering phenotypes of Arabidopsis WT, H1 triple mutant (*3h1*), and *3h1* complemented with empty vector GFP (*3h1*:GFP), Arabidopsis AtH1.1-GFP (*3h1*:AtH1.1-GFP), apple MdH1.1-GFP (*3h1*:MdH1.1-GFP), and MdH1.2-GFP (*3h1*:MdH1.2-GFP) driven by the *RPS5a* promoter, respectively. Bar = 2 cm. **B)** Flowering time expressed as the number of rosette leaves at bolting for the genotypes described in **A)**. The bottom blot shows PCR bands detected with the forward primer of *H1.1* or *H1.2* and the reverse primer of GFP (Supplementary Table S1) using genomic DNA from these 6 Arabidopsis genotypes as template, corresponding to a sequence length of 735 bp. **C)** Lateral root phenotypes for the genotypes described in (A). All images are of the same scale. Bar = 2 cm, **D)** Lateral root formation expressed as the number of lateral roots per cm root length as shown in **C)**. **E)** Root hair phenotypes for the genotypes described in **A)**. Bars = 100 μm. **F)** Root hair density as shown in **E)**. **G)** Stomatal patterning phenotypes for the genotypes described in **A)**. Bars = 50 μm. **H)** Heterochromatic chromocenters of somatic nuclei for the genotypes described in **A)**. Bars = 2 μm. **I)** Leaf malate concentrations for the genotypes described in **A)**. **J)** Subcellular localization of pCaMV35S::MdH1.1-GFP fusions in transiently transformed protoplasts of apple calli. All constructs were transiently expressed in the apple protoplasts via a PEG-mediated method. The protoplasts were stained with DAPI to locate the nuclei. Protoplasts expressing pCaMV35S::GFP and pCaMV35S::MdMYB73-GFP were used as a negative and a positive control, respectively. Bars = 10 µm. **K)** Transcription activity analysis of MdMYB73 and MdH1.1 in yeast cells. The empty vector BD was used as a negative control. Yeasts grown on SD (-Trp) medium and SD (-Trp/-Ade/-His) medium supplemented with x-α-gal (x-α-galactosidase) are indicated. **L)** RT-qPCR analysis of the expression levels of *MdH1.1*, *MdMYB73*, and *Ma1* genes in apple fruit transiently expressing antisense *MdH1.1* or antisense *MdMYB73* by the viral vector-based transformation in combination with or without 50 mm sorbitol in “Greensleeves” apple fruit. The antisense cDNA fragments of *MdH1.1* and *MdMYB73* were inserted into the TRV vector for suppression, respectively, with the empty vector as control. The relative expression level of each gene was obtained using the ddCT method, with *ACTIN* as a reference. **M)** Malate concentrations in apple fruit transiently expressing antisense *MdH1.1* or antisense *MdMYB73* by the viral vector-based transformation in combination with or without 50 mm sorbitol in “Greensleeves” apple fruit. In **B)**, **D)**, **F)**, and **I)**, data are obtained from 10 biological replicates with 3 Arabidopsis plants per replicate. In **L)**, data are mean ± SE of 5 biological replicates with 3 fruits per replicate. In **M)**, data are obtained from 5 biological replicates with 3 fruits per replicate. In **B)**, **D)**, **F)**, **I)**, and **M)**, the boxes represent interquartile ranges, with the middle lines as medians and the whiskers as the maximum and minimum values. In **B)**, **D)**, **F)**, **I)**, **L)**, and **M)**, different letters indicate significant difference using Tukey's HSD test at *P* < 0.05 after ANOVA.

As MdH1.1 is homologous to Arabidopsis linker histone protein H1.1 with a similar predicted structure (Supplementary Figs. S4 and S5), we first determined whether MdH1.1 is a linker histone. In our functional characterization, we also included MdH1.2, encoded by *MD04G1226600* that shows high sequence similarity to MdH1.1 and differs with MdH1.1 in only 5/7 amino acids in the WHD domain/globular domain (Supplementary Fig. S5). We cloned the ribosomal protein S5a (*RPS5a*) promoter from Arabidopsis and used it to drive the expression of the apple *MdH1.1* and *MdH1.2* genes, with Arabidopsis *H1.1* gene as a positive control, in the Arabidopsis H1 triple mutant *3h1* (Rutowicz et al. 2019). The triple mutant *3h1* exhibited the phenotype of earlier flowering (Fig. 5A and B), higher number of lateral roots per unit root length (Fig. 5C and D), a higher density of root hairs (Fig. 5E and F), and a higher occurrence of high-degree (tertiary and quaternary) clusters of stomates in the adaxial epidermis of cotyledons (Fig. 5G) as reported earlier (Rutowicz et al. 2019). Complementation of the *3h1* mutant with *MdH1.1* or Arabidopsis *H1.1* restored the phenotype to the WT, but *MdH1.2* or the empty vector did not (Fig. 5A to G). The *3h1* mutant failed to form the typical chromocenters in heterochromatin as normally seen in most WT somatic nuclei (Fig. 5H), which was restored to the WT by complementation with *MdH1.1* or Arabidopsis *H1.1*, but not by *MdH1.2* or the green fluorescent protein (GFP) empty vector. These data indicate that apple MdH1.1 is indeed a linker histone, an essential architectural protein of the chromatin structure. In addition, Arabidopsis *3h1* mutant had lower levels of malate and *AtALMT9* transcripts in leaves compared with the WT, which was also restored by complementation with *MdH1.1* or Arabidopsis *H1.1*, but not by *MdH1.2* or the GFP empty vector (Fig. 5I; Supplementary Fig. S10).

To confirm the subcellular localization of MdH1.1, we constructed pCaMV35S::MdH1.1-GFP fusion vectors and introduced them into protoplasts isolated from apple calli, with pCaMV35S::GFP and pCaMV35S::MdMYB73-GFP as the negative and positive controls, respectively. By visualization with a confocal microscope, we detected pCaMV35S::MdH1.1-GFP and pCaMV35S::MdMYB73-GFP fusion proteins exclusively in the nucleus, whereas the pCaMV35S::GFP control was distributed throughout the protoplast (Fig. 5J). Moreover, both MdH1.1 and MdMYB73 proteins showed autoactivation when fused with the BD vector in the yeast two-hybrid (Y2H) system, suggesting that they have transactivation activity in the single cellular yeast system (Fig. 5K).

As *Ma1* encodes a key tonoplast transporter mediating the diffusion of malate into the vacuole and a direct target gene of MdMYB73 in apple (Hu et al. 2017; Li et al. 2020a), we investigated the roles of MdH1.1 and MdMYB73 in sorbitol-modulated malate accumulation in fruit. To this end, we used the TRV vector for virus-induced gene silencing to suppress their expression. Two viral constructs, TRV-MdH1.1 and TRV-MdMYB73 were made, and each along with empty vector TRV control was combined with 50 mm sorbitol, i.e. TRV + 50 mm sorbitol, TRV-MdH1.1 + 50 mm sorbitol, TRV-MdMYB73 + 50 mm sorbitol, and TRV empty vector control for fruit infiltration. We found that *MdH1.1* and *MdMYB73* transcript levels were significantly increased in TRV + 50 mm sorbitol from the TRV control, but these increases were blocked by TRV-MdH1.1 and TRV-MdMYB73, respectively (Fig. 5L). As a direct target gene of MdMYB73, *Ma1* expression showed changes corresponding to *MdMYB73* expression (Fig. 5L). Malate concentrations of the infiltrated fruit cortex tissue were significantly higher in TRV + 50 mm sorbitol compared with the TRV control, but this stimulation effect was blocked by TRV-MdH1.1 or TRV-MdMYB73 (Fig. 5M). These results indicate that both MdH1.1 and MdMYB73 are essential for sorbitol-modulated malate accumulation in apple.

### MdH1.1 activates the transcription of *MdMYB73*, *MdCIbHLH1*, and *MdPH5* by binding to their promoters

**Figure 6.**
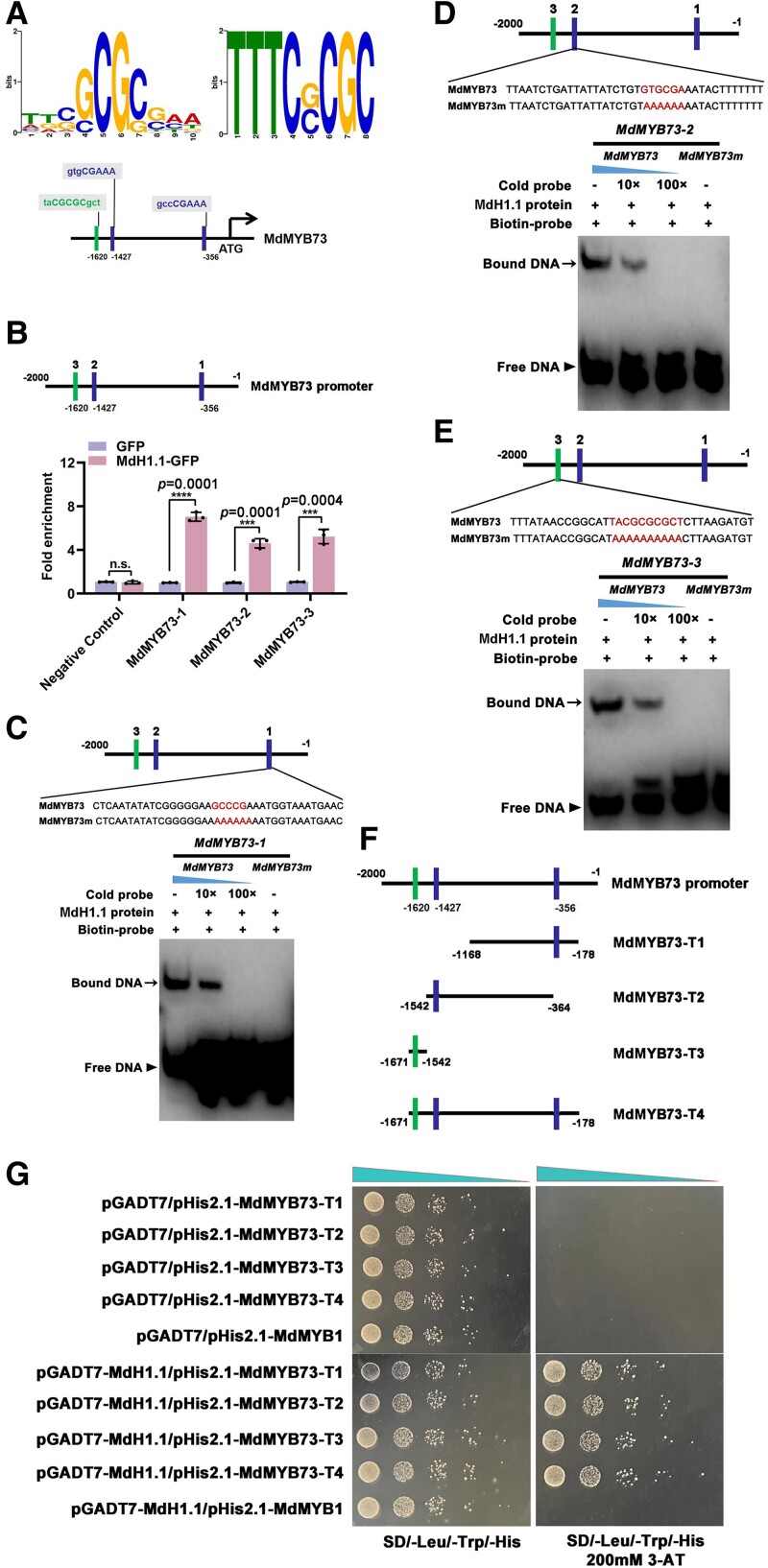
MdH1.1 binds to the promoter of *MdMYB73*. **A)** The putative WHD cis-elements in the *MdMYB73* promoter. The upper panel shows the core sequences of the WHD-binding cis-element. The bottom diagram indicates the 3 putative WHD-binding cis-elements, with green and blue nucleotides in the gray rectangles corresponding to the 2 WHD-binding motifs. **B)** ChIP-qPCR assays of the enrichment of the *MdMYB73* promoter fragments in *35S::MdH1.1-GFP* transgenic apple calli relative to *35S::GFP* transgenic apple calli. The MdH1.1-DNA complex was co-immunoprecipitated from *35S::GFP* and *35S::MdH1.1-GFP* transgenic apple calli using a GFP antibody. The 3 rectangles (1, 2, and 3) represent the positions of the putative WHD-binding elements in the *MdMYB73* promoter. The promoter region of *MdMYB1* without a WHD-binding cis-element was used as the negative control. Data are mean ± SE of 5 biological replicates with calli grown in 1 petri dish as 1 replicate. Statistical difference was determined using Student's *t*-test at *P* < 0.001 against the GFP empty vector control at an enrichment value designated as 1. **C)** EMSAs of the interaction between MdH1.1 and its first binding element in the *MdMYB73* promoter. Lane 1 shows the labeled DNA probe (MdMYB73-1) and the MdH1.1 protein without a competitor. Increasing amounts (10× and 100×) of the unlabeled DNA fragment were added in Lanes 2 and 3 as cold competitors. Lane 4 shows the labeled mutant DNA probe. **D)** EMSAs of the interaction between MdH1.1 and its second binding element in the *MdMYB73* promoter. Lane 1 shows the labeled DNA probe (MdMYB73-2) and the MdH1.1 protein without a competitor. Increasing amounts (10× and 100×) of the unlabeled DNA fragment were added in Lanes 2 and 3 as cold competitors. Lane 4 shows the labeled mutant DNA probe. **E)** EMSAs of the interaction between MdH1.1 and its third binding element in the *MdMYB73* promoter. Lane 1 shows the labeled DNA probe (MdMYB73-3) and the MdH1.1 protein without a competitor. Increasing amounts (10× and 100×) of the unlabeled DNA fragment were added in Lanes 2 and 3 as cold competitors. Lane 4 shows the labeled mutant DNA probe. **F)** Schematic representation of the different truncations of the *MdMYB73* promoter in yeast vectors. The *MdMYB73* promoter was divided into 4 fragments according to the positions of the 3 putative WHD-binding elements. **G)** Y1H assays showing that MdH1.1 binds to the promoter fragments of *MdMYB73*, containing the WHD-binding motifs. The basal concentration of 3-AT at 200 mm was used to screen interacting fragments. The empty vectors combined with different promoter fragments of *MdMYB73* (pGADT7/pHis2.1-MdMYB73-T1∼T4) were used as negative controls, with the promoter of *MdMYB1* without a WHD-binding cis-element as an additional negative control. The yeast cells were spotted with serial dilutions (1/1, 1/10, 1/100, 1/1,000, and 1/10,000) onto the yeast medium.

Considering that MdH1.1 has a WHD domain that overlaps with the histone H1 globular domain (Supplementary Fig. S5A) and both MdH1.1 and MdMYB73 are essential for sorbitol-modulated malate accumulation, we explored the relationship between MdH1.1 and MdMYB73. By analyzing the *MdMYB73* promoter, we identified 3 putative WHD-binding cis-elements (Fig. 6A). To verify in vivo binding of MdH1.1 to the *MdMYB73* promoter, we conducted chromatin immunoprecipitation (ChIP)-PCR assays by using *35S::MdH1.1-GFP* and *35S::GFP* transgenic apple calli, and found that these 3 WHD-elements-containing promoter regions of the *MdMYB73* promoter were enriched in the *35S::MdH1.1-GFP* transgenic calli, compared with the *35S::GFP* control, whereas the promoter region of *MdMYB1* without a WHD-binding cis-element (Negative control) was not (Fig. 6B).

To determine the binding of MdH1.1 to the *MdMYB73* promoter in vitro, we did electrophoretic mobility shift assays (EMSAs) by using a prokaryote-expressed and purified MdH1.1-His fusion protein. When a biotin-labeled oligonucleotide probe containing a WHD binding cis-element is used, a specific DNA-MdH1.1 protein complex was detected, but the formation of this complex was attenuated with an increasing amount of the unlabeled competitor probe with the same sequence and the complex was not detected with a mutated version of the probe (Fig. 6C to E). Furthermore, the binding specificity was corroborated by the lack of a specific DNA-MdH1.1 protein complex detected for the *MdMYB73* promoter region without a WHD binding cis-element or the *MdtDT* promoter containing a putative WHD binding cis-element but MdH1.1 does not bind to (Supplementary Fig. S11). These results demonstrate that MdH1.1 binds directly to the *MdMYB73* promoter and specifically to the WHD-binding cis-elements. We also tested the binding of MdH1.1 to the *MdMYB73* promoter via yeast one-hybrid (Y1H) assay. In this assay, various fragments of the *MdMYB73* promoter were tested, with MdMYB1, a transcription factor without a WHD binding cis-element in its promoter, as an additional negative control. MdH1.1 was found to bind to each of the fragments of the *MdMYB73* promoter containing a WHD-motif (Fig. 6F and G). In contrast to MdH1.1, MdH1.2 does not bind to the promoter of *MdMYB73* as detected by ChIP-PCR or Y1H assays (Supplementary Fig. S12).

In earlier work, we found that MdCIbHLH1 interacts with MdMYB73 in regulating malate accumulation and vacuolar acidification in apple (Hu et al. 2017). Analysis of the *MdCIbHLH1* promoter revealed the presence of 1 putative WHD-binding cis-element (Supplementary Fig. S13A). In addition, the *MdPH5* gene with lower expression detected in the fruit and leaves of the 2 antisense lines A4 and A10 earlier (Fig. 2) also has a putative WHD-binding cis-element in its promoter (Supplementary Fig. S13A). Subsequently, we conducted ChIP-PCR, EMSA, and Y1H assays and confirmed that MdH1.1 also binds directly to the promoters of *MdCIbHLH1* and *MdPH5* (Supplementary Figs. S13B to S13H).

**Figure 7.**
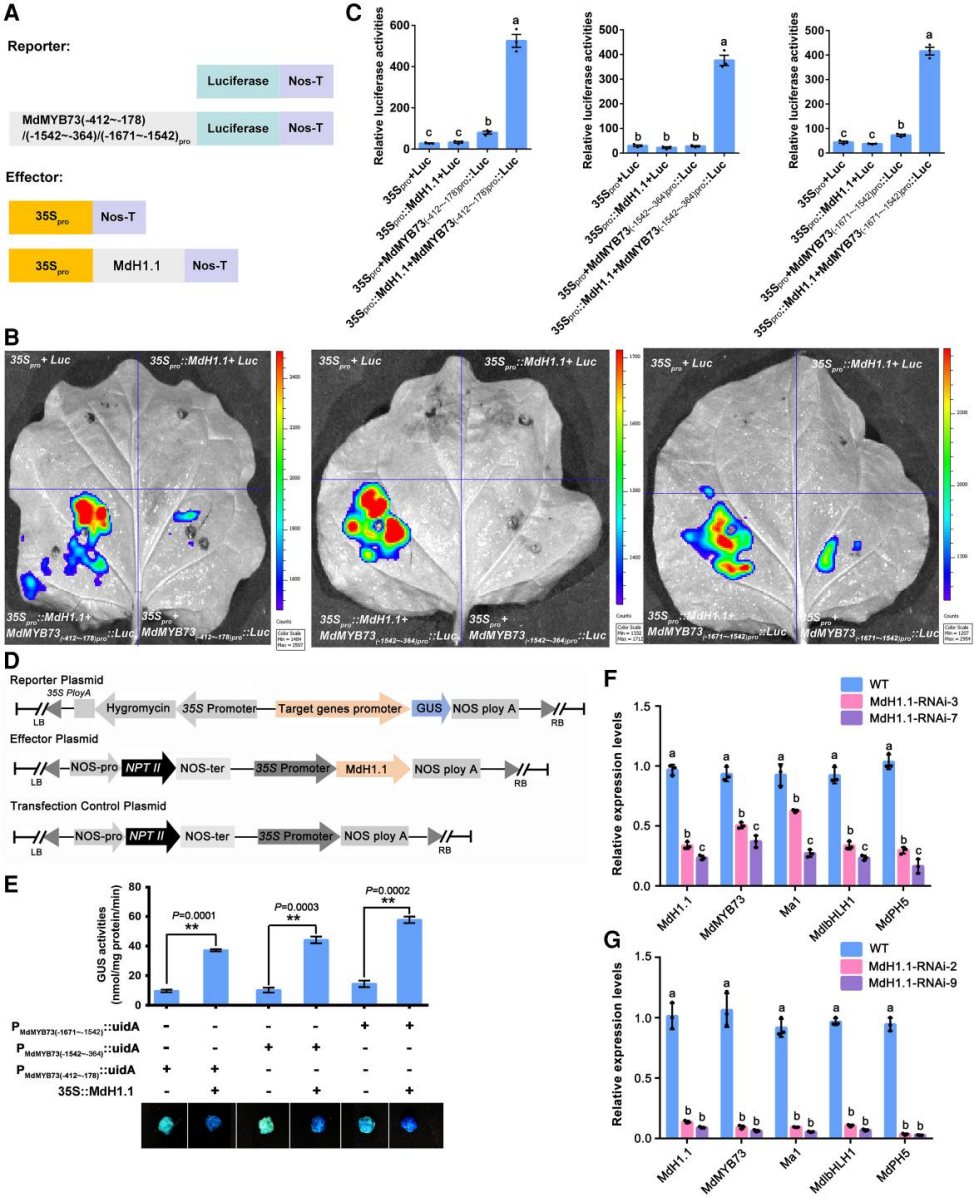
MdH1.1 activates the transcription of *MdMYB73* to enhance its expression. **A)** Schematic representation of the Luc reporter vector containing the 3 truncated promoters of *MdMYB73* (−412∼−178; −1542∼−364; −1671∼−1542) and the effector vector containing MdH1.1. **B)** Transient expression assays showing that MdH1.1 activates the expression of *MdMYB73_(−412∼−178)pro_::Luc* (left image); *MdMYB73_(−1542∼−364)pro_::Luc* (middle image); *MdMYB73_(−1671∼−1542)pro_::Luc* (right image). Representative images of *N. benthamiana* leaves 72 h after infiltration were shown. **C)** Quantitative analysis of luminescence intensity as shown in **B)**, with *35S_pro_+Luc* as a negative control. Data are mean ± SE of 3 biological replicates with 3 leaves per replicate. Different letters indicate significant difference using Tukey's HSD test at *P* < 0.01 after ANOVA. **D)** Schematic diagrams of the effector (*35S::MdH1.1*) and the reporter vectors (3 *MdMYB73* truncated promoters-GUS) for transient expression analysis. **E)** The effector and reporter constructs in the binary vectors were introduced into apple calli for GUS activity assays. *P_MdMYB73(−412∼−178)_::uidA*, *P_MdMYB73(−1542∼−364)_::uidA* and *P_MdMYB73(−1671∼−1542)_::udiA* transgenic apple calli with or without the *35S::MdH1.1* effector, were grown at 25 °C in the dark and were stained to visualize GUS activity. Data are mean ± SE of 3 biological replicates with calli grown in 1 petri dish as 1 replicate. Statistical difference was determined using Student's *t*-test at **P < 0.01. The experiment was repeated 3 times with similar results. **F)** RT-qPCR analysis of the relative expression levels of *MdH1.1*, *MdMYB73*, *Ma1*, *MdCIbHLH1*, and *MdPH5* genes in the leaves of WT “Royal Gala” apple and *MdH1.1* antisense lines (MdH1.1-RNAi-3, and MdH1.1-RNAi-7). The relative expression level of each gene was obtained using the ddCT method, with *ACTIN* as a reference. Data are mean ± SE of 3 biological replicates with 6 young leaves from 3 shoots per replicate. Different letters indicate significant difference using Tukey's HSD test at *P* < 0.05 after ANOVA. **G)** RT-qPCR analysis of the relative expression levels of *MdH1.1*, *MdMYB73*, *Ma1*, *MdCIbHLH1*, and *MdPH5* genes in the calli of WT “Orin” apple and 2 *MdH1.1* antisense lines (MdH1.1-RNAi-2 and MdH1.1-RNAi-9). The relative expression level of each gene was obtained using the ddCT method, with *ACTIN* as a reference. Data are mean ± SE of 3 biological replicates with calli grown in 1 petri dish as 1 replicate. Different letters indicate significant difference using Tukey's HSD test at *P* < 0.05 after ANOVA. The experiment was repeated 3 times with similar results.

We used dual luciferase (Luc) assays to confirm the activation of the *MdMYB73*, *MdCIbHLH1*, and *MdPH5* expression by MdH1.1. Three recombinant plasmids (*MdMYB73_pro_::Luc*, *MdCIbHLH1_pro_::Luc*, and *MdPH5_pro_::Luc*) containing *MdMYB73*, *MdCIbHLH1*, and *MdPH5* promoters fused to the reporter gene *Luciferase*, respectively, were combined with *35S_pro_::MdH1.1* or *35S_pro_* and co-infiltrated into *Nicotiana benthamiana* leaves (Fig. 7A; Supplementary Fig. S14A). Co-expression of *35S_pro_::MdH1.1* with *MdMYB73_pro_::Luc*, *MdCIbHLH1_pro_::Luc*, and *MdPH5_pro_::Luc* led to significantly higher luminescence intensities than those detected in the co-expression of *35S_pro_/MdMYB73_pro_::Luc*, *35S_pro_/MdCIbHLH1_pro_::Luc*, and *35S_pro_/MdPH5_pro_::Luc*, respectively, with no signal found in the negative controls (Fig. 7B and C; Supplementary Figs. S14B to S14E). These results indicate that MdH1.1 activates the *MdMYB73*, *MdCIbHLH1*, and *MdPH5* promoters. To further verify the activation of gene expression by MdH1.1, we transformed apple calli with *P_MdMYB73_::uidA*, *P_MdCIbHLH1_::uidA*, or *P_MdPH5_::udiA*, in combination with *35S::MdH1.1* and conducted GUS assays on the transgenic calli (Fig. 7D). The transgenic calli containing *P_MdMYB73_::uidA*, *P_MdCIbHLH1_::uidA*, or *P_MdPH5_::udiA* plus *35S::MdH1.1* exhibited significantly higher GUS activity than those harboring *P_MdMYB73_::uidA*, *P_MdCIbHLH1_::uidA*, or *P_MdPH5_::udiA* individually (Fig. 7E; Supplementary Fig. S14F and G). These results indicate that MdH1.1 activates *GUS* transcription driven by the promoters of *MdMYB73*, *MdCIbHLH1*, and *MdPH5*. In contrast to MdH1.1, MdH1.2 does not activate the *MdMYB73* promoter as detected by transient Luc assays (Supplementary Fig. S15).

To assess the role of MdH1.1 in activating downstream gene expression in apple, we transformed “Royal Gala” plants with an RNAi construct of *MdH1.1* and obtained 2 RNAi lines with ∼30% to 40% WT transcript level. RT-qPCR analysis showed that the expression of *MdMYB73*, *Ma1*, *MdCIbHLH1*, and *MdPH5* was significantly decreased in the leaves of the RNAi lines (Fig. 7F). In addition, transgenic calli of *MdH1.1* RNAi lines (2 and 9) also yielded similar results (Fig. 7G). Taken together, these results demonstrate that MdH1.1 binds to the promoter of *MdMYB73*, *MdCIbHLH1*, and *MdPH5* to activate their expression.

As MdCIbHLH1 was previously shown to interact with MdMYB73 to enhance the transactivation activity of MdMYB73 on downstream target genes such as *Ma1* (Hu et al. 2017), we determined if MdCIbHLH1 also binds to the promoter of *MdMYB73* to activate its expression. The ChIP-PCR, Y1H and Luc assays indicate that MdCIbHLH1 binds to the promoter of *MdMYB73*, activating its transcription (Supplementary Fig. S16). We hypothesized that MdH1.1 and MdCIbHLH1 operate additively in transcriptionally activating *MdMYB73*. To test this hypothesis, we performed Y1H and Luc assays. The *MdMYB73* promoter was fused to the pHis2.1 vector and *MdH1.1* and *MdCIbHLH1* to the activation domain pGADT7. When the fused pHis2.1-MdMYB73-T4 (Fig. 6F) was co-expressed with the combination of pGADT7-MdH1.1 and pGADT7-MdCIbHLH1, the transformed yeast cells exhibited better growth than those in either pGADT7-MdH1.1 + pHis2.1-MdMYB73-T4 or pGADT7-MdCIbHLH1 + pHis2.1-MdMYB73-T4 on an SD/-Leu-Trp-His/3-AT^200^ plate (Supplementary Fig. S17A). Similarly, Luc assays show that a combination of injections with MdMYB73_pro_::Luc associated with 35S_pro_::MdH1.1 and 35S_pro_::MdCIbHLH1 in *N. benthamiana* leaves resulted in a higher intensity of luminescence than either 35S_pro_::MdH1.1 + MdMYB73_pro_::Luc or 35S_pro_::MdCIbHLH1 + MdMYB73_pro_::Luc alone (Supplementary Fig. S17B and C). These results suggest that MdH1.1 and MdCIbHLH1 work together in transcriptionally activating *MdMYB73*.

Considering that MdCIbHLH1 interacts with MdMYB73 to enhance the transactivation activity of MdMYB73 on *Ma1* (Hu et al. 2017) and acts as a transcriptional activator for *MdMYB73*, we explored the possibility of a regulatory pathway by MdH1.1 via its interaction with MdCIbHLH1 to affect the transactivation activity of MdCIbHLH1 and subsequent transcription of *MdMYB73*, an alternative to its direct transcriptional regulation of *MdMYB73*. Y2H and bimolecular fluorescence complementation (BiFC) assays indicate that there is no interaction between MdH1.1 and MdCIbHLH1 proteins (Supplementary Fig. S18). In addition, we tested whether MdH1.1 interacts with a previously characterized BTB-TAZ domain protein BT2 that interacts with both MdCIbHLH1 and MdMYB73 in regulating *Ma1* expression and vacuolar acidification in response to nitrate (Zhang et al. 2020a, 2020b), but no interaction between MdH1.1 and MdBT2 proteins was detected either (Supplementary Fig. S18). Exclusion of these 2 potential indirect pathways for the action of MdH1.1 further strengthens the conclusion that MdH1.1 directly activates the transcription of *MdMYB73* by specifically binding to the WHD cis-elements in its promoter.

### MdMYB73 binds to the promoter of *MdH1.1* to enhance its expression

**Figure 8.**
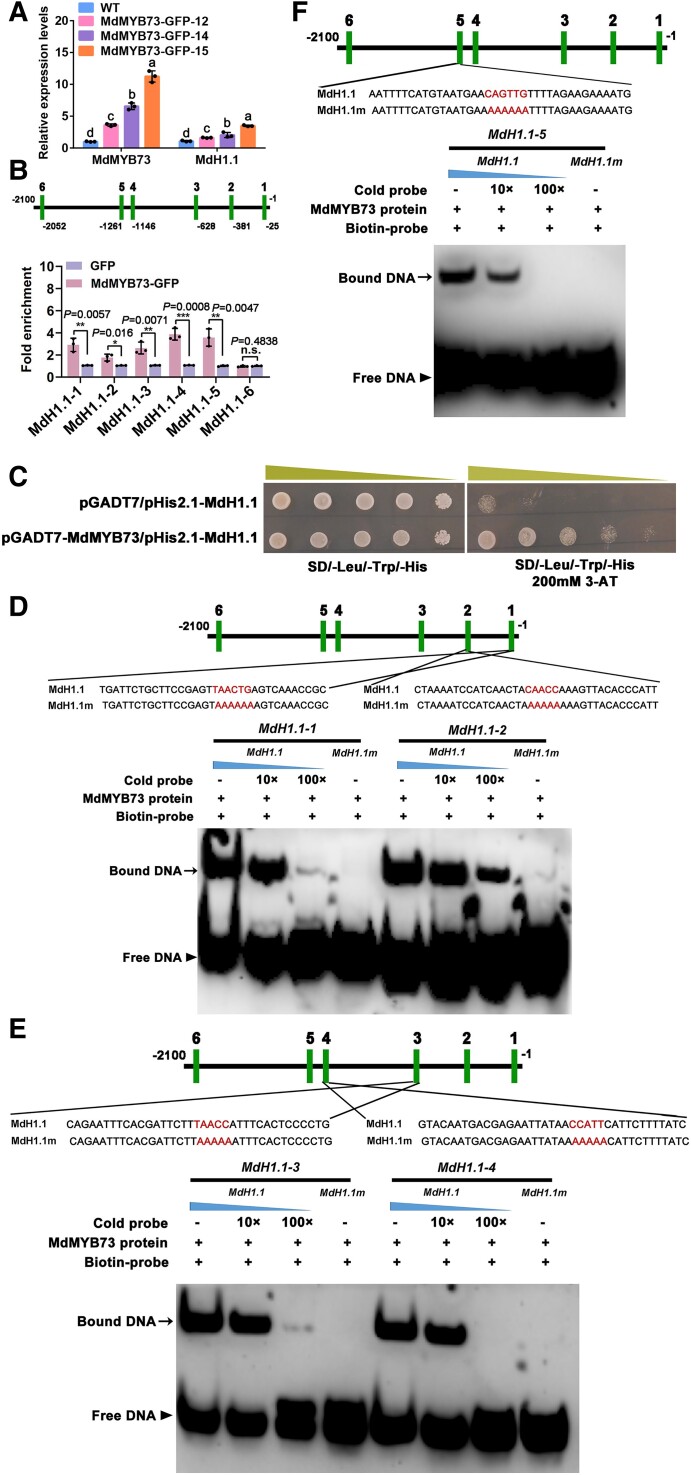
MdMYB73 specifically binds to the promoter of *MdH1.1.***A)** RT-qPCR analysis of the relative expression levels of *MdMYB73* and *MdH1.1* genes in the apple calli of WT and 3 independent *35S::MdMYB73-GFP* overexpression lines (MdMYB73-GFP-12, MdMYB73-GFP-14, and MdMYB73-GFP-15). The relative expression level of each gene was obtained using the ddCT method, with *ACTIN* as a reference. Data are mean ± SE of 3 biological replicates with calli grown in 1 petri dish as 1 replicate. Different letters indicate significant difference using Tukey's HSD test at *P* < 0.05 after ANOVA. The experiment was repeated 3 times with similar results. **B)** The relative enrichments of the *MdH1.1* promoter fragments. The MdMYB73-DNA complex was co-immunoprecipitated from *35S::MdMYB73-GFP* transgenic apple calli using a GFP antibody. *35S::GFP* was used as a negative control. The 6 bars marked as 1 to 6 represent the positions of the putative MYB-binding elements in the *MdH1.1* promoter. Data are mean ± SE of 5 biological replicates with calli grown in 1 petri dish as a replicate. Statistical difference was determined using Student's *t*-test against the GFP empty vector control at an enrichment value designated as 1. n.s. denotes none significance. *, **, and *** denote significant difference at *P* < 0.05, 0.01, and 0.001, respectively. **C)** Y1H assays showing that MdMYB73 binds to the *MdH1.1* promoter containing the MYB-binding motifs. The basal concentration of 3-AT was 200 mm. The empty vector with the *MdH1.1* promoter (pGADT7/pHis2.1-MdH1.1) was used as negative control. The yeast cells were spotted with serial dilutions (1/1, 1/10, 1/100, 1/1,000, and 1/10,000) onto the yeast medium. **D)** to **F)** EMSAs of the interaction between MdMYB73 and labeled DNA probes for the 5 binding elements (1 to 5) in the *MdH1.1* promoter. For every EMSA, Lane 1 shows the labeled DNA probe and the MdMYB73 protein without a competitor. Increasing amounts (10× and 100×) of the unlabeled DNA fragment (MdH1.1-1, 2, 3, 4, or 5) were added in Lanes 2 and 3 as cold competitors. Lane 4 shows the corresponding labeled mutant DNA probe (MdH1.1-1, 2, 3, 4, or 5 m).

Analysis of the promoter of *MdH1.1* revealed the presence of 6 putative MYB-binding cis-elements. By using 3 *35S::MdMYB73-GFP* transgenic apple callus lines obtained earlier (Hu et al. 2017), we found that *MdH1.1* expression in these transgenic apple callus lines was significantly upregulated relative to the WT control (Fig. 8A). This led us to hypothesize that MdMYB73 directly binds to the promoter of *MdH1.1* to enhance its expression. To verify this hypothesis, we performed ChIP-PCR assays on *35S::MdMYB73-GFP* and *35S::GFP* transgenic apple calli. We found that 5 binding regions in the *MdH1.1* promoter (MdH1.1-1, -2, -3, -4, -5) were enriched by ChIP in the *35S::MdMYB73-GFP* transgenic calli, but not in the 6th region (MdH1.1-6) compared with the *35S::GFP* control (Fig. 8B).

We also used Y1H assay to verify the binding of MdMYB73 to the *MdH1.1* promoter. The *MdH1.1* promoter was fused to the pHis2.1 vector, and the *MdMYB73* gene was fused to the activation domain pGADT7. When the fused pHis2.1-MdH1.1 was co-expressed with pGADT7-MdMYB73, significant growth of transformed yeast cells was observed on an SD/-Leu-Trp-His/3-AT^200^ plate, but no growth for the negative control where the pHis2.1-MdH1.1 and the pGADT7 empty vector were co-expressed (Fig. 8C).

We further performed EMSAs by using prokaryote-expressed and purified MdMYB73-His fusion proteins. A specific DNA-MdMYB73 protein complex was detected when a WHD-binding oligonucleotide was used as a labeled probe (Fig. 8D to F). The formation of this complex was reduced with an increasing amount of the unlabeled MYB competitor probe with the same sequence, and the complex was not detected with a mutated version of the probe (Fig. 8D to F). The specificity of this interaction further supports that the specific binding of MdMYB73 to the *MdH1.1* promoter requires the MYB recognition sequence.

**Figure 9.**
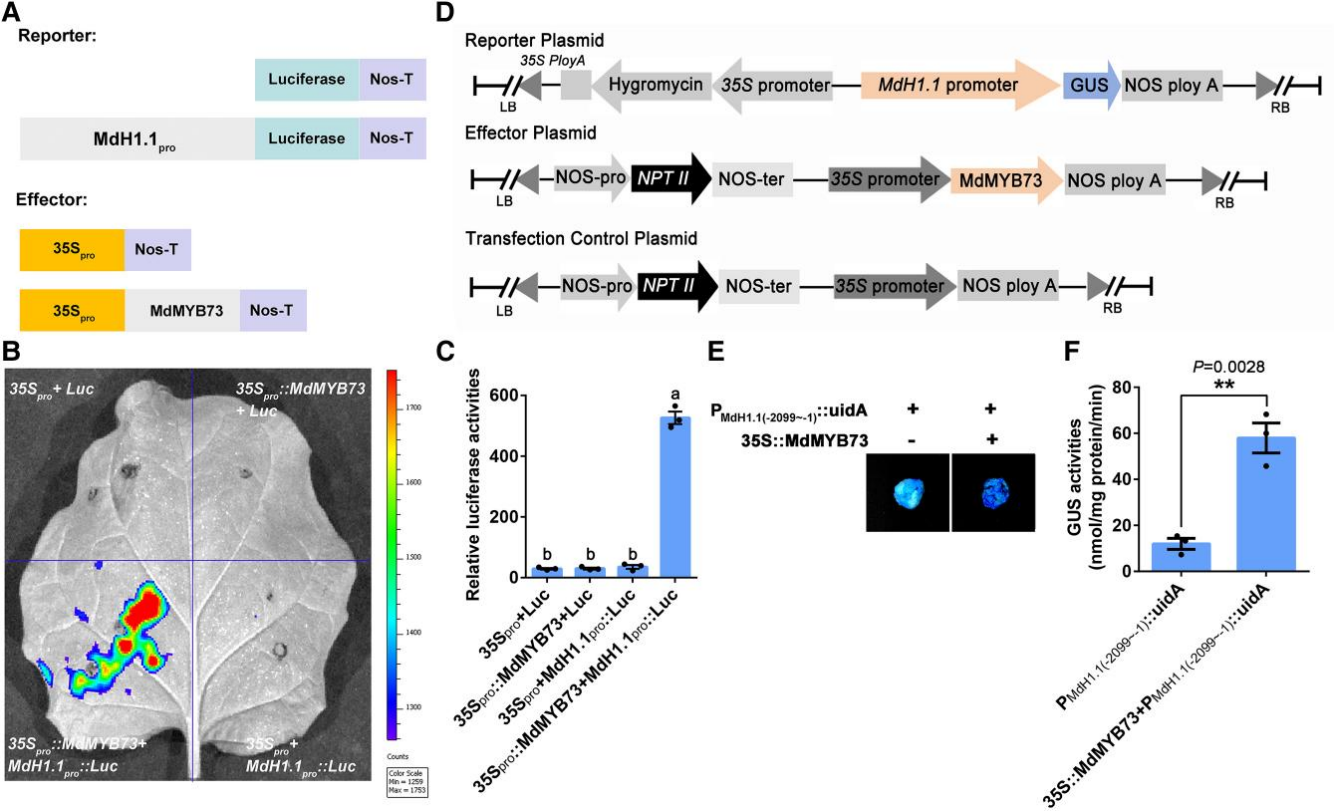
MdMYB73 activates the transcription of *MdH1.1* to enhance its expression. **A)** Schematic representation of the Luc reporter vector containing the promoter of *MdH1.1* and the effector vector containing MdMYB73. **B)** Transient expression assays showing that MdMYB73 activates the expression of *MdH1.1_pro_::Luc*. Representative images of *N. benthamiana* leaves 72 h after infiltration were shown. **C)** Quantitative analysis of luminescence intensity as indicated in **B)**. The value for luminescence intensity in *35S_pro_* + Luc samples was taken as a negative control. Data are mean ± SE of 3 biological replicates with 3 leaves per replicate. Different letters indicate significant difference using Tukey's HSD test at *P* < 0.05 after ANOVA. **D)** Schematic diagrams of the effector (*35S::MdMYB73*) and the reporter vector (*MdH1.1* promoter-GUS) for transient expression analysis. **E)** The effector and reporter constructs in the binary vectors were introduced into apple calli for GUS activity assays. *P_MdH1.1(−2099∼−1)_::uidA* transgenic apple calli, with or without the *35S::MdMYB73* effector, were grown at 25 °C in the dark and were stained to visualize GUS activity. **F)** GUS activity in the transgenic apple calli shown in **E)**. Data are mean ± SE of 3 biological replicates with calli grown in 1 petri dish as a replicate. Statistical difference was determined using Student's *t*-test at **P < 0.01.

Luc assays were used to verify the activation of the *MdH1.1* expression by MdMYB73. The recombinant plasmid *MdH1.1_pro_::Luc* containing the *MdH1.1* promoter fused to the reporter gene, *Luciferase*, was combined with *35S_pro_::MdMYB73* and infiltrated into *N. benthamiana* leaves (Fig. 9A). Weak luminescence signals were detected in the co-expression region of *35S_pro_/MdH1.1_pro_::Luc*, but not in the negative controls (Fig. 9B and C). In contrast, co-expression of *35S_pro_::MdMYB73* with *MdH1.1_pro_::Luc* exhibited significantly stronger luminescence intensity (Fig. 9B and C). These results indicate that MdMYB73 positively regulates *MdH1.1* expression.

Subsequently, we used GUS assays to further confirm the activation of *MdH1.1* by MdMYB73. The recombinant plasmid *P_MdH1.1_::uidA* was combined with *35S::MdMYB73* and transformed into apple calli (Fig. 9D). GUS staining showed that the transgenic calli containing *P_MdH1.1_::uidA* with *35S::MdMYB73* exhibited much higher GUS activities than those containing only *P_MdH1.1_::uidA* (Fig. 9E and F). These results indicate that MdMYB73 enhances the GUS transcription driven by the *MdH1.1* promoter.

### The relationship between MdH1.1 and MdMYB73 in sorbitol-modulated malate accumulation

**Figure 10.**
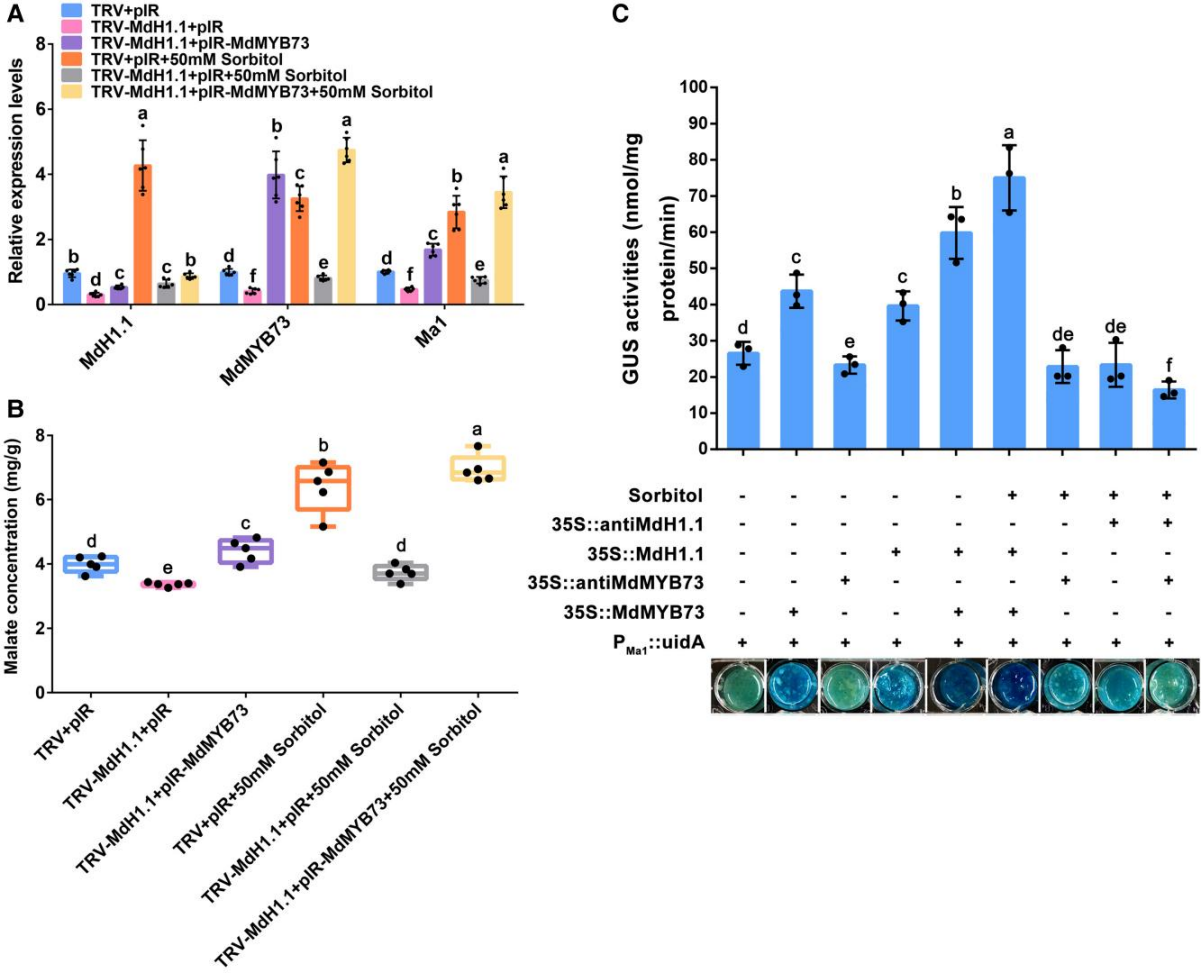
Repression of *MdH1.1* alone or in combination with overexpression of *MdMYB73* via the viral vector-based transient transformation and GUS assays with or without 50 mm sorbitol on malate accumulation in apple fruit and calli. **A)** RT-qPCR analysis of the relative expression levels of *MdH1.1*, *MdMYB73*, and *Ma1* genes in transient suppression of *MdH1.1* alone or in combination with transient overexpression of *MdMYB73* via the viral vector-based transformation with or without 50 mm sorbitol in WT apple fruit. The antisense cDNA fragment of *MdH1.1* was inserted into the TRV vector for suppression. The CDS of the *MdMYB73* gene was cloned into the pIR vector for overexpression. The empty vector TRV or pIR was used as control. The relative expression level of each gene was obtained using the ddCT method, with *ACTIN* as a reference. Data are mean ± SE of 5 biological replicates with 3 fruits per replicate. **B)** Fruit malate concentrations for the treatments described in **A)**. Data are obtained from 5 biological replicates with 3 fruits per replicate. The boxes represent interquartile ranges, with the middle lines as medians and the whiskers as the maximum and minimum values. **C)** GUS assays of transient overexpression or suppression of *MdH1.1* or *MdMYB73* genes in *P_Ma1_::uidA* transgenic apple calli in response to sorbitol feeding. The *P_Ma1_::uidA* reporter construct in the binary vector was introduced into apple calli. The *P_Ma1_::uidA* transgenic apple calli transiently overexpressing or suppressing *MdH1.1* or *MdMYB73* genes were treated with H_2_O or 50 mm sorbitol, respectively, for 12 h at 25 °C in the dark, and stained to visualize GUS activity. The *P_Ma1_::uidA* transgenic apple calli without sorbitol treatment were used as the control. *35S::MdH1.1* and *35S::MdMYB73* represent *MdH1.1* and *MdMYB73* overexpression, respectively; while *35S::antiMdH1.1* and *35S::antiMdMYB73* denote *MdH1.1* and *MdMYB73* suppression, respectively. The bar graph at the top shows the GUS activities corresponding to the representative GUS staining images at the bottom. Data are mean ± SE of 3 biological replicates with calli grown in 1 petri dish as a replicate. In **A)** to **C)**, different letters indicate significant differences between groups using Tukey's HSD test at *P* < 0.05 after ANOVA.

To determine the relationship between MdH1.1 and MdMYB73 in sorbitol-modulated malate accumulation, we used virus-based gene overexpression and virus-induced gene silencing via pRI vector and TRV vector, respectively. TRV-MdH1.1 was infiltrated into fruit alone or in combination with pIR-MdMYB73 in the presence or absence of 50 mm sorbitol, with the empty TRV and pIR vectors as controls. Inclusion of 50 mm sorbitol in the infiltration significantly increased the transcript levels of *MdH1.1*, *MdMYB73*, and *Ma1* and fruit malate concentrations (Fig. 10A and B). This stimulation effect was blocked by infiltration of TRV-MdH1.1, but infiltration of pIR-MdMYB73 restored the sorbitol-stimulated transcripts of *MdMYB73* and *Ma1* and malate accumulation (Fig. 10A and B). These data show that MdMYB73 works downstream of MdH1.1.

We also examined the function of MdH1.1 and MdMYB73 in apple calli in detail. The full-length sense open reading frames and antisense cDNA fragments of *MdH1.1* and *MdMYB73* were inserted into binary expression vectors downstream of *35S* promoters, independently, and then were transformed into apple calli separately or in combination. We obtained 7 types of transgenic apple calli, *MdH1.1-OVX*, *MdH1.1-RNAi*, *MdMYB73-OVX*, *MdMYB73-RNAi*, *MdH1.1-OVX* + *MdMYB73-OVX*, *MdH1.1-RNAi* + *MdMYB73-OVX*, and *MdH1.1-RNAi* + *MdMYB73-RNAi*. *MdH1.1* and *MdMYB73* were successfully overexpressed or suppressed in the corresponding calli compared with the WT control (Supplementary Fig. S19A). The expression of *MdMYB73* was increased or decreased by overexpression or RNAi suppression of *MdH1.1*, respectively. Similarly, the expression of *MdH1.1* was upregulated or downregulated by the overexpression or RNAi suppression of *MdMYB73*, respectively (Supplementary Fig. S19A). The transcript levels of *Ma1*, *MdCIbHLH1*, and *MdPH5* also showed corresponding changes to those of *MdH1.1* and *MdMYB73* (Supplementary Fig. S19A). As a result, the malate concentration in the transgenic calli was altered in a manner consistent with the expression of *MdH1.1* and *MdMYB73* (Supplementary Fig. S19B). By using the ratiometric fluorescent pH indicator 2′,7′-bis-(2-carboxyethyl)-5-(6)-carboxyfluorescein (BCECF), we found that the vacuolar pH of these calli showed a response just opposite to that of the malate concentration (Supplementary Fig. S19C and D). These data show that MdH1.1 works together with MdMYB73 in regulating malate accumulation and vacuolar acidification.

To assess the role of *MdCIbHLH1* and *MdPH5* in sorbitol-modulated malate accumulation, we suppressed their expression in fruit by using a TRV vector. Infiltration of TRV-MdCIbHLH1 and TRV-MdPH5 significantly decreased the expression of *MdCIbHLH1* and *MdPH5*, and fruit malate levels (Supplementary Fig. S20).

### MdH1.1, MdMYB73, and Ma1 operate in the same transcriptional regulatory pathway for sorbitol-modulated malate accumulation

Considering that *MdH1.1*, *MdMYB73*, and *Ma1* are essential for sorbitol-modulated malate accumulation (Figs. 4 and 5L and M), and MdH1.1 and MdMYB73 form a regulatory loop targeting *Ma1*, all 3 genes must operate in the same transcriptional regulatory pathway. To verify this, we combined the *P_Ma1_::uidA* construct with the overexpression vector *35S::MdH1.1* or *35S::MdMYB73*, and/or the antisense vector *35S::antiMdH1.1* or *35S::antiMdMYB73*, and genetically transformed them into apple calli. Subsequently, these transgenic calli were treated with 50 mm sorbitol and subjected to the GUS staining assay, with untreated transgenic calli as control. We found that the transgenic calli containing *P_Ma1_::uidA* plus *35S::MdH1.1* or *35S::MdMYB73* showed a significantly higher GUS activity than those harboring *P_Ma1_::uidA* alone (Fig. 10C). The GUS activity in *P_Ma1_::uidA* calli was enhanced by the overexpression of both *35S::MdH1.1* and *35S::MdMYB73*, and this enhancement of GUS activity was further improved by the presence of 50 mm sorbitol (Fig. 10C). By contrast, the GUS activity in the transgenic calli containing *P_Ma1_::uidA* plus *35S::antiMdMYB73* was lower compared with those harboring *P_Ma1_::uidA* alone (Fig. 10C), and the sorbitol treatment partially restored the GUS activity. These results indicate that MdH1.1-MdMYB73-Ma1 operate in the same transcriptional regulatory pathway.

## Discussion

It was shown previously that a low malate level resulting from either genetic control of malate accumulation in apple or a decrease in mitochondrial malate synthesis by antisense repression of the fumarase gene in tomato leads to more starch accumulation during fruit development and higher soluble solids content at harvest (Berüter 2004; Centeno et al. 2011). Here, we demonstrate another aspect of the malate-carbohydrate relationship: A sugar, specifically sorbitol in this case, acts as a signal modulating malate accumulation in apple. In response to sorbitol, a linker histone, MdH1.1, in addition to being an architectural protein for chromatin structure, functions as a transcription factor to activate the expression of *MdMYB73*, *MdCIbHLH1*, and *MdPH5* by directly binding to their promoters; MdMYB73, in turn, enhances the expression of *MdH1.1* by directly binding to its promoter. This positive feedback loop regulates the expression of *Ma1*, a key tonoplast malate transporter that mediates the diffusion of malate into the vacuole for accumulation (Hu et al. 2017; Li et al. 2020a).

The first indication that malate accumulation is linked to sorbitol level came from earlier findings that both mature fruit and leaves of the antisense *A6PR* lines have lower malate levels compared with the WT (Teo et al. 2006; Ma 2012). We confirmed that the antisense *A6PR* fruits have lower malate levels throughout fruit development, corresponding to their lower sorbitol levels (Fig. 1; Supplementary Fig. S1). By feeding leaves with equimolar concentrations of sorbitol, mannitol, fructose, glucose, and sucrose, we found that only sorbitol elicits malate accumulation, and its effect cannot be replicated with any of the other sugars tested (Fig. 4A to D; Supplementary Fig. S7). The most convincing evidence supporting sorbitol as a signal came from the findings that *pMdH1.1::GUS*, *pMdMYB73::GUS*, and *pMa1::GUS* transgenic calli treated with 50 mm sorbitol exhibited significantly greater GUS activity (Fig. 4E to J; Supplementary Fig. S9), and the sorbitol-induced malate accumulation was blocked by RNAi suppression of either *MdH1.1* or *MdMYB73*, 2 transcription factors that regulate *Ma1* expression (Fig. 5L and M). So, sorbitol serves as a signal modulating malate accumulation in apple.

In addition to being an architectural protein for chromatin structure, MdH1.1 has a winged-helix DNA-binding domain and functions as a transcription factor that specifically responds to sorbitol (Figs. 4A, E, and F and 5J and K; Supplementary Fig. S5). Several lines of evidence support its key role in sorbitol-modulated malate accumulation. First, *MdH1.1* expression is decreased in both fruit and leaves of the antisense *A6PR* lines (Fig. 3C), but is stimulated by exogenous sorbitol feeding (Fig. 4A, E, and F). Second, it binds to the WHD cis-elements in the promoter of *MYB73*, a transcriptional activator for *Ma1*, *MdVHA-A*, and *MdVHP1* (Hu et al. 2017), as demonstrated via ChIP-PCR, EMSA, and Y1H assays of the interaction (Fig. 6). Third, both Luc and GUS activity assays show transcriptional activation of *MYB73* by MdH1.1 (Fig. 7A to E). Fourth, transcriptional activation of *MYB73* by MdH1.1 in response to sorbitol leads to higher *Ma1* expression and higher malate levels (Figs. 5L and M and 10A and B). Finally, MdH1.1 activates the expression of *MdCIbHLH1*, a transcription factor that activates the expression of *MdMYB73* and interacts with MdMYB73 in regulating *Ma1* expression, and *MdPH5*, a proton-pumping P-ATPase for vacuolar acidification, to enhance the sorbitol-modulated malate accumulation (Supplementary Figs. S13, S14, S16, and S19). The winged-helix DNA-binding protein superfamily includes many transcription factors with diverse functions such as heat shock factors (HSFs), early region 2 promoter-binding factor (E2F) transcription factors, and linker histones. HSFs are involved in regulating plant responses to heat stress and other abiotic and biotic stresses (Charng et al. 2007; Andrási et al. 2021), whereas E2F transcription factors are key regulators for plant cell proliferation, differentiation, and photomorphogenesis (De Veylder et al. 2002; Ramirez-Parra et al. 2004; Sozzani et al. 2006; Lopez-Juez et al. 2008). Of the linker histones characterized to date, MdH1.1 is the only one that has been demonstrated to act as a transcription factor directly regulating the expression of 3 genes (*MdMYB73*, *MdCIbHLH1*, and *MdPH5*) involved in malic acid accumulation. It would be interesting to test if this is a conserved function of linker histone H1 across plant species considering its involvement in malate accumulation in both apple and Arabidopsis (Fig. 5I and M) and to identify its target genes other than those characterized here. Specific binding of MdH1.1 to the WHD cis-elements in the promoters of the 3 genes (Fig. 6; Supplementary Fig. S13) in addition to complementation of the Arabidopsis *3h1* mutant as a linker histone (Fig. 5A-I) suggests that MdH1.1 has the capability of sequence specific binding as well as nonsequence specific binding to DNAs, but how this is accomplished deserves further research. It is also interesting that MdH1.2, which differs from MdH1.1 in only a small number of amino acids in the globular domain/WHD domain, does not complement the Arabidopsis *3h1* mutant or bind to the promoter of *MdMYB73* (Fig. 5A-I; Supplementary Figs. S12 and S15). Exploring these differences might allow us to identify the key amino acids in the globular domain/WHD domain of H1s in the future.

**Figure 11.**
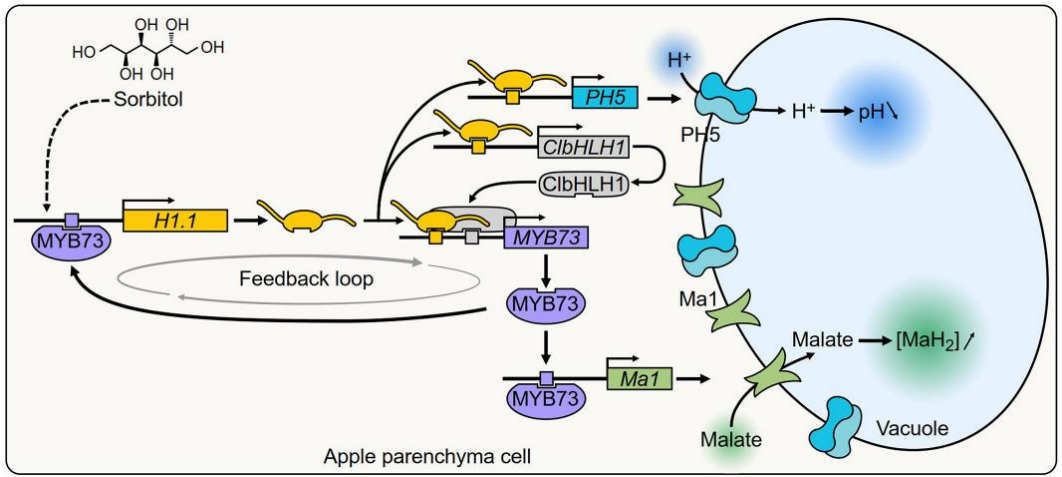
A working model for sorbitol-modulated malate accumulation in apple via linker histone H1.1. Apple linker histone H1.1 functions as a transcription factor, with its gene expression responding specifically to sorbitol, activating the expression of the MYB transcription factor gene *MYB73*, the cold-induced basic/helix-loop-helix transcription factor gene *CIbHLH1*, and the P-ATPase gene *PH5*. MYB73, in turn, enhances the expression of *H1.1*, augmenting the sorbitol signal. This positive feedback loop promotes the expression of the *Ma1* gene encoding aluminum-activated malate transporter ALMT9 (Ma1), which mediates the transport of malate into the vacuole across the tonoplast (Li et al. 2020a). In addition, the regulatory loop may also synergize Ma1-mediated malate transport with vacuolar acidification via the interaction of CIbHLH1 with MYB73 and its transcriptional activation of *MYB73*, enhancing the expression of the genes encoding vacuolar ATPase subunit A and vacuolar pyrophosphatase 1 as well as *Ma1* (Hu et al. 2017). Note: There are multiple binding sites for MYB73 and H1.1 in the promoters of *H1.1* and *MYB73*, respectively, but only 1 binding site is shown for each transcription factor in this model for simplicity.

*MdMYB73* is not only a target gene of MdH1.1, but its protein also transcriptionally regulates *MdH1.1*, in return. This reciprocal regulation between MdH1.1 and MdMYB73 forms a positive feedback loop for sorbitol modulation of vacuolar malate accumulation, a unique feature of the regulatory system. The initial clue for the existence of such a regulatory loop came from the finding that transgenic apple calli overexpressing *MdMYB73* had significantly higher expression of *MdH1.1* (Fig. 8A). Subsequent ChIP-PCR assay, EMSAs, Y1H assay, Luc assay and GUS activity measurements confirmed the binding of the MdMYB73 to the promoter of *MdH1.1* and transcriptional activation of its expression (Figs. 8 and 9). It has been shown that the expression of *At*MYB23 and *MdMYB10*/*MdWRKY10* is enhanced by binding to their own promoters to define cell fate in the root epidermis of Arabidopsis (Kang et al. 2009) and anthocyanin synthesis in the flesh of apple (Espley et al. 2009; Wang et al. 2024), respectively. In pepper (*Capsicum annuum*), CabZIP63 binds to the promoter of *CaWRKY40* as well as its own, with positive feedback transcriptional regulation of *CabZIP63* by CaWRKY40 in an unidentified manner, regulating the defense response to *Ralstonia solanacearum* inoculation or high temperature and high humidity (Shen et al. 2016). The operation of the MdH1.1-MdMYB73 regulatory loop for malate accumulation allows for amplification of the sorbitol signal as demonstrated by higher and lower expression of *MdH1.1*, *MdCIbHLH1*, and *MdPH5* in the *MYB73*-OE and *MYB73*-RNAi calli, respectively (Supplementary Fig. S19). In addition, considering that MdMYB73 transcriptionally regulates *MdVHA-A* and *MdVHP1* as well as *Ma1* (Hu et al. 2017) and MdH1.1 activates the expression of *MdCIbHLH1* and *MdPH5* as well as *MdMYB73*, the MdH1.1-MdMYB73 feedback loop may enable better coordination between vacuolar acidification by proton pumps and vacuolar malate transport mediated via *Ma1*. Based on the findings presented here and elsewhere (Hu et al. 2017; Li et al. 2020a), we propose a working model for sorbitol-modulated malate accumulation in apple (Fig. 11). Sorbitol is somehow sensed, upregulating *MdH1.1* expression. MdH1.1 binds to the promoters of *MdMYB73*, *MdCIbHLH1*, and *MdPH5* to activate their expression, and MdMYB73 enhances *MdH1.1* expression, in return, to augment the sorbitol signal. The MdH1.1-MdMYB73 regulatory loop coordinates the transcriptional regulation of *Ma1* with those involved in vacuolar acidification to enhance malate accumulation in the vacuole.

ALMT9 mediates malate transport across the tonoplast in Arabidopsis (Kovermann et al. 2007), tomato (Ye et al. 2017) and apple (Li et al. 2020a). By targeting the transcriptional regulation of *MdALMT9* (*Ma1*), sorbitol signaling links vacuolar malate accumulation to plant carbohydrate/energy status. In addition to altering the storage of carbon and energy in the form of malate in the vacuole, the modulation of vacuolar malate transport can potentially have several functions in both leaves and fruit. First, as plant glycolysis is largely regulated by a feedback mechanism via phosphoenolpyruvate (Plaxton 1996), transport of malate into the vacuole may alter phosphoenolpyruvate concentration, together with feedforward regulation of glycolysis by G6P (Plaxton and Podestá 2006), allowing glycolysis to be adjusted to plant carbohydrate status. Second, sugar-deprived sycamore cells show a decrease in cytosolic pH upon sugar addition (Gout et al. 2011). This suggests that high carbohydrate supply may acidify the cytosol, and transport of malate into the vacuole may help maintain cytosolic pH homeostasis (Smith and Raven 1979; Kenyon et al. 1985; Hurth et al. 2005; Arias et al. 2013). Finally, malate circulation between chloroplasts and mitochondria via the cytosol is critical for controlling the generation of reactive oxygen species by mitochondria (Zhao et al. 2020), so transport of malate into the vacuole may mitigate the level of reactive oxygen species under high carbohydrate status. While doing so in the fruit, it also enables fruit acidity, an important component of fruit taste and flavor, to respond to carbohydrate status. This may explain why apples produced from trees with a high source to sink ratio have higher acidity as well as higher soluble solids (Serra et al. 2016; Anthony et al. 2019), rendering better and more intense taste and flavor.

How sorbitol is sensed and the signal transduced to *MdH1.1* remains unknown. In glucose signaling, hexokinase 1 (HXK1) senses glucose to initiate the signaling cascade for regulating plant development and responses to the changing environment (Jang et al. 1997; Moore et al. 2003; Hu et al. 2016c; Sun et al. 2018). In sucrose signaling, sucrose nonfermenting-1-related kinase 1 (SnRK1) senses sucrose as an indicator of plant energy status, modulating plant developmental processes, assimilate partitioning, and stress tolerance (Chiou and Bush 1998; Yang et al. 2004; Jia et al. 2013; Liu et al. 2017). Sorbitol signaling could be initiated by SDH in a manner analogous to HXK1 in glucose sensing, and the transcripts of apple *SDHs* respond to sorbitol (Archbold 1999; Zhou et al. 2006). However, although most SDHs have the highest affinity to sorbitol, they also use other polyols as substrates (Ohta et al. 2005) so the issue of substrate specificity must be dealt with for sorbitol-specific signaling. Another possibility is that sorbitol signaling shares SnRK1 with sucrose signaling, as has been demonstrated in the regulation of the transcript levels of *SDH1* and *A6PR* via phosphorylation of bZIP39 by SnRK1 in response to sorbitol (Meng et al. 2023). It was shown recently that sorbitol modulates apple flower development and pollen tube growth by regulating the expression of *STP13a* and other genes via MYB39L (Meng et al. 2018a; Li et al. 2020b) as well as resistance of apple leaves to *A. alternata* by controlling the expression of *NLR16* via WRKY79 (Meng et al. 2018b). So, sorbitol signaling appears to play a broad role in many processes in apple. The characterization of the MdH1.1-MYB73 regulatory loop for sorbitol-modulated malate accumulation, together with MYB39L, WRKY79, bZIP39, and SnRK1 identified earlier, may eventually lead us to uncover the entire molecular network of sorbitol signaling in the future.

Why apple opted for sorbitol as a signal during evolution is unclear, but at least 2 scenarios can be envisioned. One is based on sorbitol being an energy-rich sugar (alcohol). As sorbitol is further reduced from glucose, it contains more energy on an equimolar carbon basis (Loescher 1987), and as such it may have evolved to be an indicator of plant carbohydrate/energy status in sorbitol-synthesizing species. This is consistent with sorbitol signaling in apple flower development and pollen tube growth reported earlier (Meng et al. 2018a; Li et al. 2020b). As a key intermediate metabolite in the glycolysis and TCA cycle, malate concentration in the cytosol and other subcellular compartments is tightly regulated by intracellular transport, with its accumulation in the vacuole serving as a carbon/energy store and a buffer for cytosolic pH homeostasis (Sakano 2001; Hurth et al. 2005; Fernie and Martinoia 2009; Selinski and Scheibe 2019). In this case, sorbitol signaling would enable coordination of malate accumulation with plant sugar status. The other scenario has to do with sorbitol being a compatible solute, as other sugar alcohols are (Williamson et al. 2002; Zhifang and Loescher 2003). Sorbitol accumulates under drought, cold, and salinity stress in apple (Wang and Stutte 1992; Kanayama et al. 2006) and other sorbitol-synthesizing species (Ranney et al. 1991; Lo Bianco et al. 2000; Al Hassan et al. 2016), which is associated with upregulation of *A6PR* expression (Kanayama et al. 2006; Liang et al. 2012) and downregulation of SDH activity (Lo Bianco et al. 2000). Malate is also a compatible solute that accumulates in response to cold, drought and salinity stress. Both winter rye (*Secale sereal*) and alpine plant *Ranunculus glacialis* accumulate high levels of malate during cold acclimation (Crecelius et al. 2003; Streb et al. 2003). A reduction in malate accumulation in Arabidopsis *tdt* knockout mutants lowers their salinity tolerance (Emmerlich et al. 2003), whereas overexpression of cytosolic malate dehydrogenase in apple increases malate accumulation and tolerance to salinity and cold stress (Wang et al. 2016). So, sorbitol signaling may have evolved to coordinate the accumulation of malate with sorbitol for the plants to cope with abiotic stresses in a more effective manner. Indeed, MYB73, one of the key transcription factors involved in sorbitol-modulated malate accumulation, also plays an important role in cold tolerance of both Arabidopsis and apple by acting like an inducer of C-repeat binding factor expression (Fowler and Thomashow 2002; Zhang et al. 2017). The cold-induced transcription factor, MdCIbHLH1 regulates apple cold hardiness (Feng et al. 2012; Wang et al. 2021) as well as malate accumulation (Supplementary Fig. S19; Hu et al. 2017; Zhang et al. 2020a). The 2 scenarios proposed here are not mutually exclusive to each other as sorbitol can serve as both an energy store and a compatible solute at the same time.

It is currently not known if sorbitol signaling operates in species where sorbitol is not a major end-product of photosynthesis and phloem transport sugar. However, recent work demonstrated that sorbitol is more widespread than originally thought. All the 42 angiosperm species representative of monocots and eudicots examined so far including Arabidopsis, grape, tomato, rice (*Oryza sativa*), and maize (*Zea mays*) have at least 1 sorbitol dehydrogenase gene (Jia et al. 2015), with both Arabidopsis and tomato SDHs showing the highest affinity to sorbitol among the polyols tested (Ohta et al. 2005; Aguayo et al. 2013). This suggests the presence of sorbitol in all these species. Indeed, sorbitol has been detected in Arabidopsis (Nosarzewski et al. 2012), grape (Conde et al. 2015), and tomato (Tari et al. 2010; Almaghamsi et al. 2021), and abiotic stress such as drought, salinity, and low temperature enhances its accumulation (Almaghamsi et al. 2021). In these species, sorbitol can be synthesized via A6PR and S6P phosphatase as in pome and stone fruits of Rosaceae and Plantago species. Two *A6PR* genes were recently identified in Arabidopsis and their expression was upregulated by cold and salt stress (Rojas et al. 2019). In tomato, activities of both A6PR and S6P phosphatase and *A6PR* transcripts were detected and responded to water stress (Almaghamsi et al. 2021). Alternatively, sorbitol can be synthesized via the reduction of fructose in the reverse reaction of SDH. While SDH primarily converts sorbitol to fructose in apple, pear, and peach due to its much higher affinity to sorbitol over fructose (Negm and Loescher 1979; Yamaguchi et al. 1994; Oura et al. 2000; Hartman et al. 2014), the SDHs in grape, tomato, and maize can reduce fructose to sorbitol owing to their smaller Km values for fructose relative to those in apple, pear, peach and plum (Doehlert 1987; Ohta et al. 2005; Conde et al. 2015). In grape, water stress enhances the accumulation of both sorbitol and mannitol, and this corresponds to upregulation of SDH and mannitol dehydrogenase activities for fructose reduction and the expression of a plasma membrane polyol transporter, *VvPLT1* (Conde et al. 2015). In the developing endosperm of maize kernel, sorbitol is synthesized in response to high availability of fructose, and SDH transcript level and enzyme activity is upregulated by sucrose (Doehlert 1987; de Sousa et al. 2008). Similarly, in germinating seeds of both soybean (Kuo et al. 1990) and Arabidopsis (Nosarzewski et al. 2012), sorbitol synthesis coincides with high fructose availability derived from β-oxidation of fatty acids. The function of sorbitol remains unclear in these cases, but in light of our findings reported here, it is possible that the elevated sorbitol level signals a high sugar status for malate accumulation, as well as a high reduction status for generating NAD^+^ (de Sousa et al. 2008). Exploring the extent to which sorbitol-modulated malate accumulation occurs in the species traditionally deemed as nonsorbitol synthesizing species may shed light on the function and evolution of sorbitol signaling in plants.

## Materials and methods

### Plant materials and growth conditions

Untransformed WT “Greensleeves” apple (of *Ma1Ma1* genotype at the *Ma* locus) (*M. domestica* Borkh) and 2 transgenic lines with antisense suppression of *A6PR* (A4 and A10) were used in this study. The transcript levels of *A6PR* in the mature leaves of A4 and A10 were about 10% of that in WT control, with lower sorbitol and higher sucrose levels in A4 and A10 than in WT control (Cheng et al. 2005; Meng et al. 2018b; Li et al. 2020b). Four-year-old trees of WT, A4, and A10, grafted onto M.26 rootstock, were grown in 20-L containers outside at Cornell Experimental Orchards for analysis of developmental changes of fruit sugars and malate, and RNA-seq and RT-qPCR of gene expression. They were arranged in a completely randomized design with 5 single-tree replicates per genotype. In addition, another set of 3-year-old WT, A4 and A10 trees propagated via tissue culture and grown in 7.6-L containers were replicated 6 times per genotype with 4 trees per replicate in a completely randomized design for analysis of leaf metabolites and gene expression and leaf sugar feeding experiments. All the trees received standard horticultural management and disease and insect control throughout the growing season. The fruiting trees of WT, A4, and A10 were moved into a bee-proof screenhouse for the entire bloom period to prevent escape of transgenic pollen.

Two RNAi-*MdH1.1* transgenic lines along with the WT of “Royal Gala” grafted onto G.11 rootstock were grown in 7.6-L containers outside at Cornell Experimental Orchards. They were replicated 5 times with 2 tree per replicate in a completely randomized design for gene expression analysis.

Calli of WT “Orin” apple (of *Ma1ma1* genotype at the *Ma* locus) were grown on MS medium plus 1.5 mg L^−1^ 6-BA and 0.5 mg L^−1^ IAA at 25 °C in the dark. They were subcultured every 2 weeks for 3 times before being used for genetic transformation and in other assays.

The *Arabidopsis thaliana* plants used in all experiments were in the Col-0 background. The *H1* triple (T-DNA) mutant, *h1.1h1.2h1.3*, abbreviated as *3h1*, was described before (Rutowicz et al. 2019). Seeds were surface sterilized and rinsed in sterile water before transferring onto germination medium (0.5× MS medium, 0.8% (w/v) agar). They were stratified for 2–4 d at 4 °C and transferred into a plant growth chamber with a long-day photoperiod (16 h/8 h) and a day/night temperature of 22 °C/18 °C at a photon flux density of ∼120 μmol m^−2^ s^−1^. Lateral root production and root hair density were evaluated under continuous light (∼100 μmol m^−2^ s^−1^) as described (Rutowicz et al. 2019). Stomata patterning on the adaxial epidermal peel of 14-day-old cotyledons were characterized as described (Kutter et al. 2007; Rutowicz et al. 2019). Flowering time was expressed as the number of rosette leaves a plant has developed when inflorescence was about 0.5 cm long.

### Feeding leaves and fruit with exogenous sugars

Fully expanded leaves of WT, A4, and A10 lines were fed with 50 mm mannitol, sorbitol, fructose, glucose or sucrose via the transpiration stream as described (Zhou et al. 2006). The leaves were cut from shoots around 7:30 AM with a razor blade first, and then immediately re-cut under water before being brought to the lab. They were randomly assigned to each sugar treatment, with petiole immersed in sugar solution of a 1.5 mL centrifuge tube. Each sugar treatment was replicated 3 times, with 9 leaves per replicate. These centrifuge tubes were inserted into Styrofoam in an angle that keeps the leaves flat and randomly arranged in a fume hood (2 m × 1.2 m × 1.5 m) under fluorescent lights at photosynthetically active radiation of about 100 μmol photons m^−2^ s^−1^ at room temperature. Leaf samples (3 leaves per replicate) were collected at 0, 3, and 6 h after initiation of sugar feeding, frozen in liquid nitrogen and stored at −80 °C for use.

For injection of exogenous sugars into fruit, a gauge needle with 0.45 mm caliber was used to poke a hole (1–2 mm deep) perpendicular to the fruit surface, and then 250 µL solution of 50 mm mannitol, sorbitol, fructose, glucose, or sucrose was injected into the hole via a 1-mL BD needleless syringe. The injection was done slowly with some pressure, and typically 3 injections distant to each other were made on the equator per fruit. In addition, the same volume of 1% (w/v) acid fuchsin, a water-soluble dye, was injected into a few fruits to delineate the contour of the distribution of the injection for guiding tissue sampling later. After injection, the fruit were left at room temperature (23 °C) for 24 h under dark conditions, and then placed at 16 °C for 7 d. The fruit were checked periodically, and the infiltrated cortex tissue was collected based on the distribution of the water-soluble dye, frozen in liquid nitrogen and stored at −80 °C for RNA extraction and other analyses.

### Sampling of fruit and leaves and analysis of soluble sugars and malate concentrations with gas chromatography-mass spectrometry (GS-MS)

Fruit samples were taken from noon to 2:00 PM at 15, 30, 60, 90, and 120 DAB, corresponding to 5 key developmental stages: active cell division, end of cell division, early rapid cell expansion, late rapid cell expansion, and fruit maturity. Four to six well-exposed fruits on the south side of the canopy were collected from each single-tree replicate, immediately cut into pieces after removing the core and frozen in liquid nitrogen, and then stored at −80 °C. Four recently fully expanded leaves were taken from actively growing shoots of 2 trees per replicate at noon under full sunlight, frozen in liquid nitrogen and then stored at −80 °C.

Soluble sugars and malate concentrations of fruit and leaves of WT, A4, and A10, and “Orin” calli were analyzed by GC-MS. Soluble sugars and malate were extracted from 0.1 g samples in 75% (v/v) methanol, with ribitol added as internal standard (Wang et al. 2010). After fractionation of the nonpolar metabolites into chloroform, 1 to 5 μL of the aqueous phase was taken for drying under vacuum without heat, and derivatized with methoxyamine hydrochloride and N-methyl-N-trimethylsilyl-trifluoroacetamide sequentially for separation and analysis with 7890A GC/5975C MS (Agilent Technology) on a DB-5MS capillary column (20 m × 0.18 mm × 0.18 μm) with a 5-m Duraguard column (Agilent Technology) as previously described (Wang et al. 2010).

### Measurements of fruit titratable acidity and soluble solids at harvest

Fruit titratable acidity and soluble solids were measured at harvest on the pooled juice sample from 6 fruits taken for each single-tree replicate, with an autotitrator (Metrohm 848 Titrino Plus and Metrohm 869 Compact Sample Changer, Herisau, Switzerland) and a PAL-1 digital refractometer (ATAGO, USA), respectively.

### RNA extraction and reverse transcription quantitative PCR

Total RNA was extracted from fruit, leaves, and calli tissues using the modified CTAB method as described (Gasic et al. 2004). After digestion with DNase I (Thermo Fisher Scientific), 2 µg of total RNA was reverse transcribed to cDNA using the iScript cDNA Synthesis Kit (Bio-Rad). RT-qPCR analysis was performed with iQ SYBR Green Supermix in an iCycler iQ5 system (Bio-Rad) following the manufacturer's instructions. Each reaction was replicated 3 times per biological replicate, with *ACTIN* as an internal reference gene. The relative expression of each gene was calculated using the 2^−ΔΔCT^ method (Udvardi et al. 2008). Sequences of the primers used for RT-qPCR are listed in the Supplementary Table S1.

### RNA-seq analysis

Total RNA was extracted from fruit or leaves with the modified CTAB method (Gasic et al. 2004). mRNA was isolated from 5 μg of total RNA using NEBNext Poly(A) mRNA Magnetic Isolation Module kit (New England Biolabs, Ipswich, MA, USA) after treatment with DNase I (RNase free) (Thermo Fisher Scientific, Waltham, MA, USA) for each sample. A total of 30 fruit cDNA libraries (5 replicates each for WT and A4 fruit at 15, 60, and 120 DAB), 15 leaf cDNA libraries (5 replicates each for the fully expanded leaves of WT, A4, and A10), and 10 leaf cDNA libraries (5 replicates each for water control and 50 mm sorbitol feeding of A10 leaves at 3 h) were constructed as described (Zhong et al. 2011). The libraries were sequenced at Cornell University CLC Genomics and Epigenomics Core facility in 50 bp single-end sequencing on an Illumina HiSeq 4000 sequencer (Illumina, Inc., San Diego, CA, USA).

The total RNA-seq reads were processed by removing adaptor sequences, potential rRNA contaminating reads identified in database Silva with Bowtie (allowing up to 3 mismatches per read) (Langmead et al. 2009), polyA/T, and virus sequences. The cleaned reads were aligned to the doubled haploid apple genome using Tophat, allowing 1 mismatch per read (Trapnell et al. 2009). Following alignments, raw counts of mapped reads for each apple gene model were derived and then normalized to the reads per kilobase of exon model per million mapped reads. DEGs between A4 and WT and between water control and sorbitol feeding of A10 leaves were identified via an R package DESeq (Anders and Huber 2010) using cut-off criteria of fold change > 1.5 and *P*-value < 0.05 for A4/WT fruits and Sorbitol/H_2_O for A10 leaves.

### Phylogenetic analysis

Full-length amino acid sequences of 15 Arabidopsis histone H1 globular (GH1)-domain proteins were obtained from the Arabidopsis Information Resource. Two apple H1 protein sequences were retrieved from the apple genome GDDH13 v1.1 database and confirmed by sequencing the cloned cDNAs. Arabidopsis GH1-domain proteins including 3 canonical H1 variants (AtH1.1, AtH1.2, and AtH1.3) were aligned with the apple H1 proteins (MdH1.1 and MdH1.2) using ClustalW 2.10. Phylogenetic analysis of MdH1.1, MdH1.2, and 15 Arabidopsis GH1-domain proteins was performed using MEGA software version 11.0 by the neighbor-joining method with 1000 bootstrap iterations.

### Plasmid construction and genetic transformation

Sense full-length sequence of *MdH1.1* was amplified via PCR and cloned into pGWB451 vector with a C-terminal GFP tag through Gateway BP and LR reactions (BP Clonase cat. no. 11789013 and LR Clonase cat. no. 11,791,019; Invitrogen). Sense full-length sequence of *MdMYB73* was amplified via PCR, and the product was inserted into the pCXSN::GFP vector under the control of the *35S* promoter. The antisense sequences of *MdH1.1* and *MdMYB73* were amplified via PCR and cloned into pGWB-RNAi vector via Gateway BP and LR reactions as described (Meng et al. 2018b). These constructs were transformed into *Agrobacterium* strain LBA4404, and positive clones were screened via colony PCR. Genetic transformation of “Orin” apple calli was performed as described (Hu et al. 2016b). All the PCR primers are listed in Supplementary Table S1.

The full-length coding sequences of *MdH1.1*, *MdH1.2*, and *AtH1.1* were recombined into the pGWB540 vector driven by the promoter of *RIBOSOMAL PROTEIN SUBUNIT 5A* (*RPS5a*) of Arabidopsis for their overexpression fused with GFP in Arabidopsis *3h1* mutant, with the empty pGWB540 vector harboring the *RPS5a* promoter-driven GFP expression as control. The constructed expression vectors were transformed into *Agrobacterium* strain GV3101 and the resultant strains were used for the transformation of Arabidopsis by the floral-dip method (Clough and Bent 1998).

A 174-bp cDNA fragment of *MdH1.1* was cloned via BP reaction into pDONR221 vector and recombined via LR reaction into the destination vector pGWRNAi (Meng et al. 2018b). The pGWRNAi-*MdH1.1* was transformed into “Royal Gala” apple (of *Ma1ma1* genotype at the *Ma* locus) as described previously (Meng et al. 2023; Li et al. 2024) to generate stably transformed RNAi-*MdH1.1* lines.

### Chromatin analyses

Freshly harvested roots of 1–2 cm long from 15-day-old Arabidopsis plants were fixed in the Cannoy Solution (methanol:acetic acid = 3:1 v/v) and kept until use. The roots were washed with distilled water twice at 5 min each. The root tips were cut and kept in the solution of 2% (w/v) cellulase + 1% (w/v) pectinase at 37 ℃ for 50–100 min. They were washed with distilled water twice at 5 min each. The root tips were fixed again with the same fixative solution. Squashes were made in precooled slides and flame dried. The heterochromatin analyses were carried out as described (Rutowicz et al. 2019).

### Subcellular localization of MdH1.1

Protoplasts isolated from *35S::GFP* and *35S::MdH1.1-GFP* transgenic apple calli were prepared as described (Hu et al. 2017). They were then stained with the DNA-speciﬁc dye 4′, 6-diami-dino-2-phenylindole (DAPI), which highlights the nucleus. The GFP signals were detected at 500 to 530 nm after excitation at 488 nm, with the gain value set at 700, on a Zeiss 810 laser scanning microscope. A total of 20–30 apple protoplasts were imaged for each construct.

### Trans-acting activity assay and Y2H assay

The CDSs of *MdMYB73* and *MdH1.1* were inserted into the pGBKT7 (BD) vector. The resulting vectors MdMYB73-BD and MdH1.1-BD, and negative (BD) controls were transformed into yeast strain AH109 as described (Bai et al. 2019). The positive transformants were selected on plates containing SD/-Trp/-Ade/-His medium. The trans-activity was assessed by the blue color formed after adding x-α-gal to the medium.

The CDSs of *MdCIbHLH1* and *MdBT2* were ligated into pGBT9 vector, and *MdH1.1* was constructed into pGAD424 vector, respectively. The paired vectors were transformed into the Y2H Gold yeast strain by PEG/LiAc transformation and grown on SD -Leu/-Trp medium. After 3–6 d of incubation, the clones were transferred onto SD -Ade/-His/-Trp/-Leu medium and incubated at 30 °C for 3–4 d.

### BiFC assays

The CDSs of *MdCIbHLH1* and *MdBT2* were cloned into the pSPYNE vector, and the CDSs of *MdH1.1* and *MdBT2* were cloned into the pSPYCE vector, respectively. The plasmids were introduced into *Agrobacterium tumefaciens* LBA4404. The various combinations were injected into 3-week-old *N. benthamiana* leaves. The GFP signals were detected at 500 to 530 nm after excitation at 488 nm, with the gain value set at 700, on a Zeiss 810 laser scanning microscope.

### Promoter analysis and ChIP-PCR assay

The promoter sequences of *MdH1.1*, *MdMYB73, MdCIbHLH1*, and *MdPH5* were obtained from the Genome Database for Rosaceae (https://www.rosaceae.org/). The analysis of putative WHD-binding sites, MYB-binding sites, and bHLH-binding sites (G-box) were conducted via the PlantCARE (plant cis-acting regulatory elements) website (http://bioinformatics.psb.ugent.be/webtools/plantcare/html/).

ChIP-PCR analyses were conducted on the *35S::MdH1.1-GFP* (*pGWB451-MdH1.1*), *35S::MdMYB73-GFP* (*pCXSN::MdMYB73-GFP*), *35S::MdH1.2-GFP* (*pFCHG20-MdH1.2*), *35S::MdCIbHLH1-GFP* (*pFCHG20-MdCIbHLH1*), and *35S::GFP* transgenic apple calli as previously described (Hu et al. 2019), using the primers listed in Supplementary Table S1.

### Electrophoretic mobility shift assay

EMSAs were performed as described (Hu et al. 2019). *MdH1.1* and *MdMYB73* were cloned into the expression vector PET-32a-c. The MdH1.1-His and MdMYB73-His recombinant proteins were expressed in *Escherichia coli* strain BL21 and purified using glutathione sepharose beads (Thermo Scientific, San Jose, CA, USA). The oligonucleotide probes were labeled with an EMSA probe biotin labeling Kit (Beyotime) according to the manufacturer's instructions. The MdH1.1-His and MdMYB73-His recombinant proteins were incubated with 10× binding buffer, 1 μg/μL poly (dI-dC), and 400 fmol biotin-labeled double-stranded binding consensus oligonucleotides in a total volume of 20 μL using a LightShift Chemiluminescent EMSA Kit (Thermo). The binding reaction was performed at room temperature for 20 min. The DNA-protein complexes were separated on 6.5% (w/v) nondenaturing polyacrylamide gels, electrotransferred, and detected following the manufacturer's instructions. The binding specificity was also examined by competition with 10 and 100-fold excess of the unlabeled probe and a mutated probe for each binding site. The sequences of the oligonucleotide probes for EMSAs are listed in Supplementary Table S1.

### Y1H assay

The Yeast One-Hybrid System-Matchmaker Gold Kit (catalog No. 630491; Clontech) was used for Y1H assays. The promoter fragments of *MdMYB73*, *MdH1.1*, *MdCIbHLH1*, and *MdPH5* generated by PCR were cloned into the pHis2.1 vector using the primers listed in Supplementary Table S1. The vectors were transformed into yeast strain Y1H Gold after linearization. Positive clones were selected through PCR and used to determine the minimal inhibitory concentration of 3-amino-1,2,4-triazole (3-AT). The CDS of *MdH1.1*, *MdMYB73*, *MdH1.2*, and *MdCIbHLH1* was cloned into the pGADT7 vector to generate pGADT7-MdH1.1, pGADT7-MdMYB73, pGADT7-MdH1.2, and pGADT7-CIbHLH1, which were then transformed into yeast strain Y1H Gold competent cells carrying the promoters of pHis2.1-MdMYB73, pHis2.1-MdH1.1, pHis2.1-MdCIbHLH1, or pHis2.1-MdPH5. Co-transformed positive clones were spotted in a series of dilutions (1:1, 1:10, 1:100, 1:1,000, and 1:10,000) and cultured on the SD/-Leu-Trp-His plate with or without 200 mm 3-AT at 30 °C for 3 to 4 d to test for possible interactions.

### GUS analysis

The promoters of *MdH1.1*, *MdMYB73*, *Ma1*, *MdCIbHLH1*, and *MdPH5* were ampliﬁed and inserted into the reporter gene PBI121-uidA. The resulting recombinant vectors *P_MdH1.1_::uidA*, *P_MdMYB73_::uidA*, *P_Ma1_::uidA*, *P_MdCIbHLH1_::uidA*, and *P_MdPH5_::uidA* were then transformed into “Orin” apple calli via *A. tumefaciens* strain LB4404. Subsequently, *35S::MdH1.1* or *35S::MdMYB73-GFP* were co-transformed into the above-mentioned transgenic calli. In addition, the CDS of *MdH1.1* and *MdMYB73* was inserted into the pIR overexpression viral vector under the control of the 35S promoter for overexpression, whereas the fragments of *MdMYB73* and *MdH1.1* were inserted into the TRV viral vector for suppression. These viral vector plasmids were used for transient infection of the co-transformed calli. Histochemical staining was performed as described (Hu et al. 2017).

### Dual Luc assay

Transient expression assays in *N. benthamiana* leaves were performed as described (Hu et al. 2019). The promoters of *MdH1.1*, *MdMYB73*, *Ma1*, *MdCIbHLH1*, and *MdPH5* were amplified and cloned into pGreenII 0800-Luc vectors to generate the reporter constructs *MdH1.1_pro_::Luc*, *MdMYB73_pro_::Luc*, *Ma1_pro_::Luc*, *MdCIbHLH1_pro_::Luc*, and *MdPH5_pro_::Luc*, respectively. The effectors (35S_pro_::MdH1.1, 35S_pro_::MdH1.2, 35S_pro_::MdCIbHLH1, and 35S_pro_::MdMYB73) were constructed by cloning the CDS of *MdH1.1*, *MdH1.2*, *MdCIbHLH1*, and *MdMYB73* into the pGreenII 62-SK vector, respectively. A NightOWL II LB983 in vivo imaging system with Indigo software (BERTHOLD TECHNOLOGIES GmbH & Co. KG, Germany) was used to collect the Luc images and quantify luminescence intensity. Transformed leaves were sprayed with and soaked in 100 mm luciferin, after which they were placed in darkness for 6 min before luminescence examination. Luciferase activity in each sample was normalized to the internal control renilla luciferase activity. Luminescence was expressed on an arbitrary scale as relative light units.

### Protoplasts isolation and measurement of vacuolar pH

Protoplasts were isolated from “Orin” apple calli as described (Sun et al. 2017). The protoplasts were then used for vacuolar pH determination by using 2′,7′-Bis(2–carboxyethyl)-5(6)-carboxyfluorescein acetoxymethyl ester (BCECF-AM; Molecular Probes, Eugene, OR) as described (Hu et al. 2016b) with some modifications. When cells were incubated at low pH, BCECF could not be easily encapsulated into the vacuole, leading to its accumulation in the cytoplasm. To mitigate the problem, we increased the dye incubation time to 6 h and adjusted the osmotic potential of the solution to 200 mOsmol kg^−1^ with sorbitol upon exposure to hypotonic medium (10 mm EGTA, 0.8 m Mannitol, 1 m MgCl_2_, 0.3 m MES and 10 μM BCECF-AM). After incubation, the protoplasts were washed 2 times with W5 solution (5 m NaCl, 1 m CaCl_2_, 1 m KCl and 0.3 m MES). These protoplasts were then kept in WI solution (0.8 m Mannitol, 1 m MgCl_2_, and 0.3 m MES), and used for vacuolar pH determination. Imaging was performed on a Zeiss LSM880 microscope at excitation wavelengths of 488 and 458 nm with fluorescence emissions detected between 520 and 540 nm. The vacuolar pH was quantified by the ratio of emission values excited with 488 and 458 nm.

### Construction of viral vectors and transient expression in apple fruit

To construct antisense expression viral vectors, fragments specific to *MdH1.1*, *MdMYB73*, *MdCIbHLH1*, and *MdPH5* were amplified with PCR using apple fruit cDNA, and the products were cloned into the TRV vector in the antisense orientation under the control of the dual *35S* promoter, which were named TRV-MdH1.1, TRV-MdMYB73, TRV-MdCIbHLH1, and TRV-MdPH5, respectively. To generate overexpression viral vectors, full-length cDNA of MdH1.1 and MdMYB73 were inserted into the pIR vector under the control of the *35S* promoter and were named pIR-MdH1.1 and pIR-MdMYB73.

The antisense expression viral vectors were transformed into *A. tumefaciens* strain GV3101 and the *Agrobacterium* was grown to an OD value of 0.6. Plasmids for overexpression were prepared to a concentration of 2,000 ng/mL. Fruit infiltration was performed as previously described with some modifications (Zhang et al. 2020a). A gauge needle with 0.45 mm caliber was used to poke a hole (3–4 mm deep) perpendicular to the fruit surface, and then 250 μL solution of agrobacterium (for antisense suppression) or plasmids (for overexpression) was injected into the hole via a 1-mL BD needleless syringe. The injection was done slowly with some pressure, and typically 3 to 4 injections distant to each other were made on the equator per fruit. In addition, the same volume of the water-soluble dye acid fuchsin was injected into a few fruits to determine the contour of the distribution of the injection as a guide for tissue sampling later. After injection, the fruit were left at room temperature (23 °C) for 24 h under dark condition, and then placed at 16 °C for 7 to 14 d. The fruit were checked periodically to remove any that has agrobacterium outgrowth. Fruit cortex tissues were collected based on the distribution of the water-soluble dye, frozen in liquid nitrogen, and stored at −80 °C for RNA extraction and other analyses.

### Statistical analyses

Statistical analyses of the data were conducted using Student's *t*-test for experiments involving 2 groups, whereas analysis of variance (ANOVA) followed by Tukey's honestly significant difference (HSD) test was used for experiments involving more than 2 groups.

### Accession numbers

Sequence data from this article can be found in the APPLE GENOME AND EPIGENOME (https://iris.angers.inra.fr/gddh13/) under accession numbers MdH1.1 (MD12G1243700), MdH1.2 (MD04G1226600), MdMYB73 (MD08G1107400), MdtDT (MD05G1358600), MdALMT9/Ma1 (MD16G1045200), MdCIbHLH1 (MD14G1148600), MdPH1 (MD15G1317200), MdPH5 (MD17G1155800), and MdBT2 (MD06G1161300).

## Supplementary Material

koae328_Supplementary_Data

## Data Availability

The data supporting this article will be shared on reasonable request to the corresponding author.

## Supplementary data

The following materials are available in the online version of this article.

**Supplementary Figure S1.** Concentrations of sorbitol, sucrose, fructose, glucose, galactose, and malate in fully expanded leaves of the wild-type (WT) control and *A6PR* antisense lines (A4 and A10) of “Greensleeves” apple.

**Supplementary Figure S2.** Genome-wide RNA sequencing (RNA-seq) analysis of fruit and leaves of the wild-type (WT) and *A6PR* antisense lines (A4 and A10) of “Greensleeves” apple.

**Supplementary Figure S3.** RNA-seq analysis of fully expanded leaves of *A6PR* antisense line A10 in response to feeding with 50 mm sorbitol or H_2_O (control).

**Supplementary Figure S4.** Phylogenetic analysis of apple and Arabidopsis histone H1 globular (GH1)-domain proteins.

**Supplementary Figure S5.** Conserved domain analysis of apple H1.1, H1.2 and Arabidopsis H1.1 and predicted structures.

**Supplementary Figure S6.** Functional testing of MdH1.1, MdMYB73, MdWRKY53, and MdWRKY53L for their involvement in sorbitol-mediated malate accumulation.

**Supplementary Figure S7.** The concentrations of mannitol, fructose, sucrose, and sorbitol in fully expanded leaves of the WT control and *A6PR* antisense lines (A4 and A10) in response to sugar feeding.

**Supplementary Figure S8.** The fruits of WT and *A6PR* antisense lines A4 and A10 injected with various sugars at a concentration of 50 mm.

**Supplementary Figure S9.** The expression of *MdH1.1*, *MdMYB73*, or *Ma1* is not induced by mannitol, galactose or myo-inositol.

**Supplementary Figure S10.** The expression levels of *AtH1.1*, *MdH1.1*, *MdH1.2*, and *AtALMT9* in Arabidopsis seedlings of wild-type (WT), H1 triple mutant *3h1*, and *3h1* complemented with GFP, AtH1.1-GFP, MdH1.1-GFP or MdH1.2-GFP.

**Supplementary Figure S11.** EMSAs show no interaction between MdH1.1 and biotin-labeled DNA probes for the *MdMYB73* promoter without a WHD binding cis-element or the *MdtDT* promoter containing a putative WHD binding *cis*-element but MdH1.1 does not bind to.

**Supplementary Figure S12.** MdH1.2 does not bind to the promoter of *MdMYB73*.

**Supplementary Figure S13.** MdH1.1 binds to the promoters of *MdCIbHLH1* and *MdPH5*.

**Supplementary Figure S14.** MdH1.1 activates the transcription of *MdCIbHLH1* and *MdPH5*.

**Supplementary Figure S15.** MdH1.2 does not activate the transcription of *MdMYB73*.

**Supplementary Figure S16.** MdCIbHLH1 binds to the promoter of *MdMYB73*, activating its transcription.

**Supplementary Figure S17.** Yeast one-hybrid (Y1H) and dual luciferase (Luc) assays showing that MdH1.1 together with MdCIbHLH1 enhance the transcription activity of *MdMYB73*.

**Supplementary Figure S18.** No interaction detected between MdH1.1 and MdCIbHLH1 or MdBT2 proteins.

**Supplementary Figure S19.** MdH1.1 works together with MdMYB73 to modulate malate accumulation and vacuolar acidification.

**Supplementary Figure S20.** MdCIbHLH1 and MdPH5 play a role in the accumulation of malate in apple fruit.

**Supplementary Table S1.** List of PCR primers used in this study.

**Supplementary Data Set 1.** Annotation of all unigenes detected via RNA-seq in the fruit of *A6PR* antisense line A4 vs. the wild-type (WT) collected at 15, 60 and 120 days after bloom (DAB).

**Supplementary Data Set 2.** Annotation of all unigenes detected via RNA-seq in the fully expanded leaves of the *A6PR* antisense lines (A4 and A10) vs. the wild-type (WT).

**Supplementary Data Set 3.** Annotation of all unigenes detected via RNA-seq in the fully expanded leaves of *A6PR* antisense line A10 in response to 50 mm sorbitol feeding vs. water control.

**Supplementary File 1.** Text file of alignment used for phylogenetic analysis.

**Supplementary File 2.** Newick file of tree for phylogenetic analysis.

## Dive Curated Terms

The following phenotypic, genotypic, and functional terms are of significance to the work described in this paper:

EMSA Gramene: electrophoretic mobility shift assay
EMSA Araport: electrophoretic mobility shift assay

LUC Gramene: luciferase
LUC Araport: luciferase

MdH1.1 Gramene: MD12G1243700
MdH1.1 Araport: MD12G1243700

MdMYB73 Gramene: MD08G1107400
MdMYB73 Araport: MD08G1107400

MdtDT Gramene: MD05G1358600
MdtDT Araport: MD05G1358600

MdALMT9 Gramene: MD16G1045200
MdALMT9 Araport: MD16G1045200

MdH1.2 Gramene: MD04G1226600
MdH1.2 Araport: MD04G1226600

MdCIbHLH1 Gramene: MD14G1148600
MdCIbHLH1 Araport: MD14G1148600

MdPH1 Gramene: MD15G1317200
MdPH1 Araport: MD15G1317200

MdPH5 Gramene: MD17G1155800
MdPH5 Araport: MD17G1155800

MdBT2 Gramene: MD06G1161300
MdBT2 Araport: MD06G1161300
