# Supplementary material for: A linker histone acts as a transcription factor to orchestrate malic acid accumulation in apple in response to sorbitol
Source: Plant Cell. 2024 Dec 20;37(1):koae328. doi: 10.1093/plcell/koae328 (PMC11773815; doi:10.1093/plcell/koae328)
Supplement: koae328_Supplementary_Data [file koae328_supplementary_data.zip › TPC2024-RA-00586R2_Supplementary Data_011425_Final.pdf]

The Plant Cell Supplementary Data Figures S1-S20 and Supplementary Table S1

The Title:

A Linker Histone Acts as a Transcription Factor to Orchestrate Malic Acid Accumulation in Apple in Response to Sorbitol

The full names of all the authors:

Da-Gang Hu[a,b], Mengxia Zhang[a], Chunlong Li[a], Ting-Ting Zhao[b], Lian-Da Du[b], Quan-Sun[b], Chu-Kun Wang[a,b], Dong Meng[a], Cui-Hui Sun[a,b], Zhangjun Fei[c], Abhaya M. Dandekar[d], Lailiang Cheng[a,1]

The names and address of the institution:

[a]Section of Horticulture, School of Integrative Plant Science, Cornell University, Ithaca, New York 14853, USA

[b]National Research Center for Apple Engineering and Technology; Shandong Collaborative Innovation Center for Fruit & Vegetable Quality and Efficient Production; College of Horticulture Science and Engineering, Shandong Agricultural University, Tai'an, Shandong 271018, China

[c]Boyce Thompson Institute, Ithaca, NY 14853, USA

[d]Department of Plant Sciences, University of California at Davis, Davis, California 95616, USA

[1]Corresponding author: Lailiang Cheng (lc89@cornell.edu)

Short Title: Sorbitol modulates malate accumulation

One-sentence Summary: Sorbitol modulates malic acid accumulation in apple by enhancing the expression of a linker histone that acts as a transcription factor for promoting the expression of a tonoplast malate transporter.

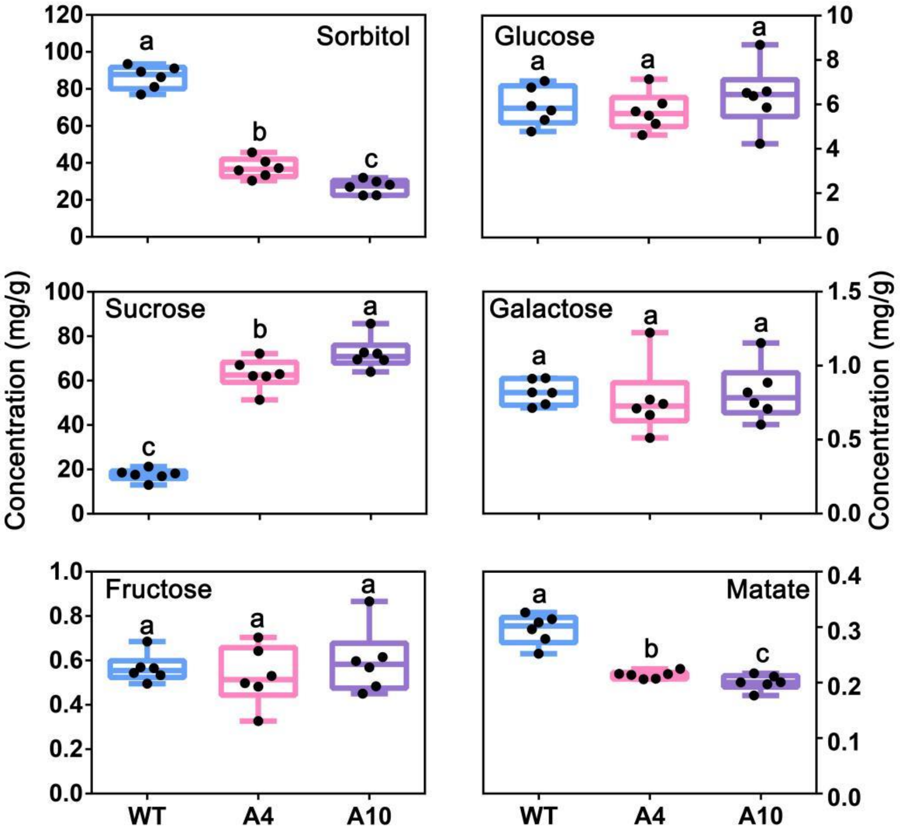

Supplementary Figure S1. Concentrations of sorbitol, sucrose, fructose, glucose, galactose, and malate in fully expanded leaves of the wild-type (WT) control and *A6PR* antisense lines (A4 and A10) of 'Greensleeves' apple.

Data are obtained from 6 biological replicates with 4 leaves pooled from two trees per replicate. The boxes represent interquartile ranges, with the middle lines as medians and the whiskers as the maximum and minimum values. Different letters (a, b, c) indicate significant difference using Tukey's HSD test at $P < 0.05$ after one-way ANOVA.

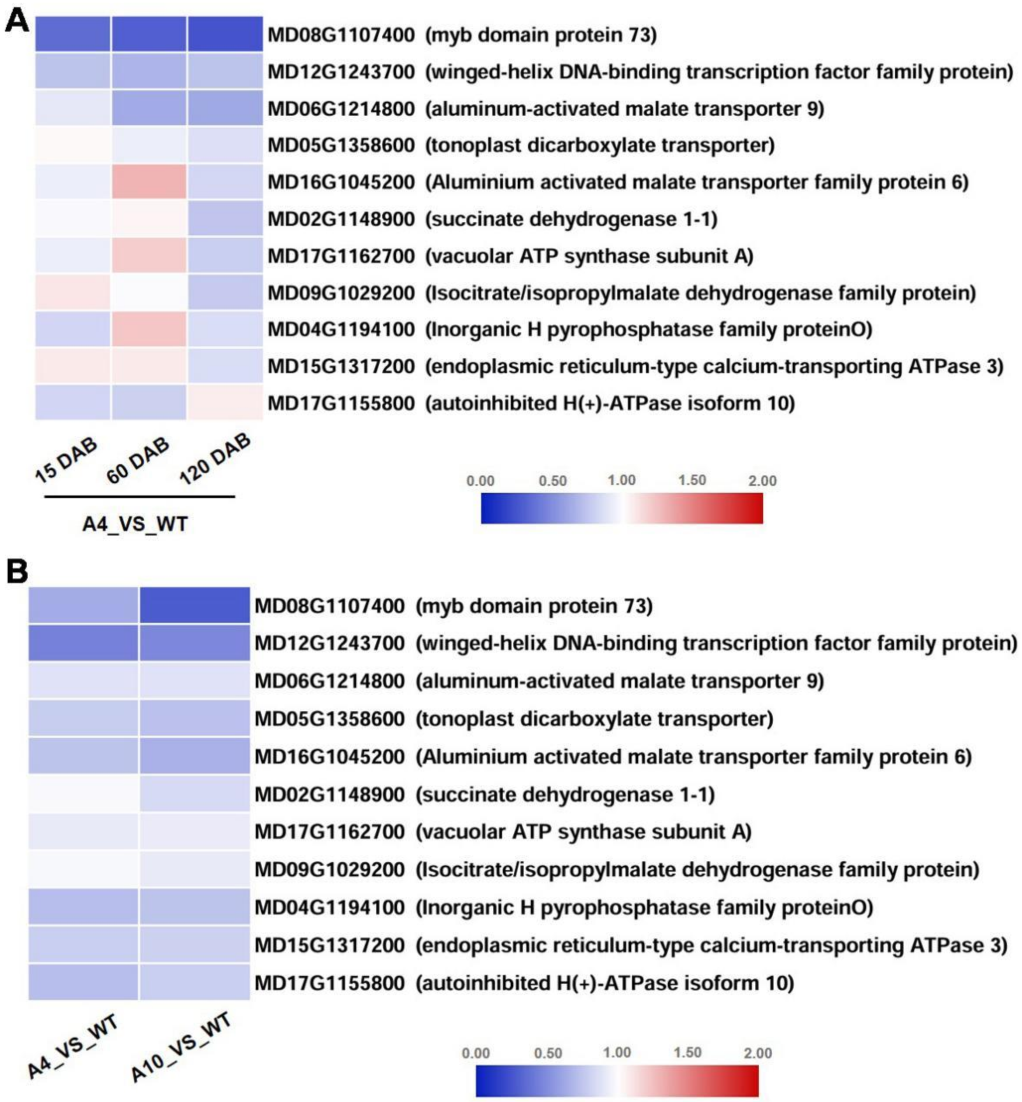

Supplementary Figure S2. Genome-wide RNA sequencing (RNA-seq) analysis of fruit and leaves of the wild-type (WT) and *A6PR* antisense lines (A4 and A10) of 'Greensleeves' apple.

(A) Heatmap showing the expression ratios of genes associated with malate accumulation in A4 vs. WT fruits collected at 15, 60 and 120 days after bloom (DAB). Means of 5 biological replicates with 4 to 6 fruits per replicate are shown, with complete data given in Supplementary Dataset S1. The scale bar is for the expression ratio of A4_vs_WT fruits.

(B) Heatmap showing the expression ratios of genes associated with malate accumulation in the leaves of A4 vs. WT, and A10 vs. WT. Means of five biological replicates with 15 leaves per replicate (Wu et al., 2015) are shown, with complete data given in Supplementary Dataset S2. The scale bar is for the expression ratio of A4_vs_WT or A10_vs_WT leaves.

Note: *MD16G1045200* is *MdALMT9/Ma1* in the ‘Golden Delicious’ doubled haploid reference genome, GDDH13, corresponding to *MDP0000252114* named initially in Bai et al. (2012), although it is annotated as a homolog of Arabidopsis *ALMT6* here.

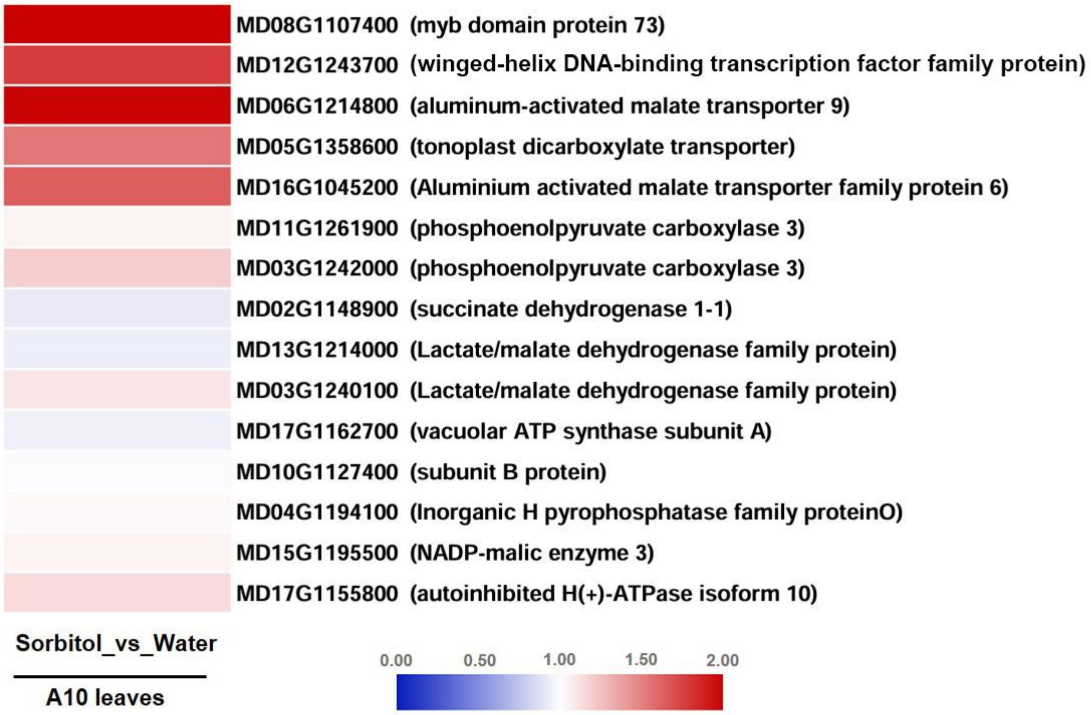

Supplementary Figure S3. RNA-seq analysis of fully expanded leaves of *A6PR* antisense line A10 in response to feeding with 50 mM sorbitol or H_2O (control). Heatmap showing the expression ratios of genes associated with malate accumulation. Means of five biological replicates with three leaves per replicate are shown, with complete data given in Supplementary Dataset S3. The scale bar is for the expression ratio of Sorbitol_vs_Water in A10 leaves.

Note: *MD16G1045200* is *MdALMT9/Ma1* in the 'Golden Delicious' doubled haploid reference genome, GDDH13, corresponding to *MDP0000252114* named initially in Bai et al. (2012), although it is annotated as a homolog of Arabidopsis *ALMT6* here.

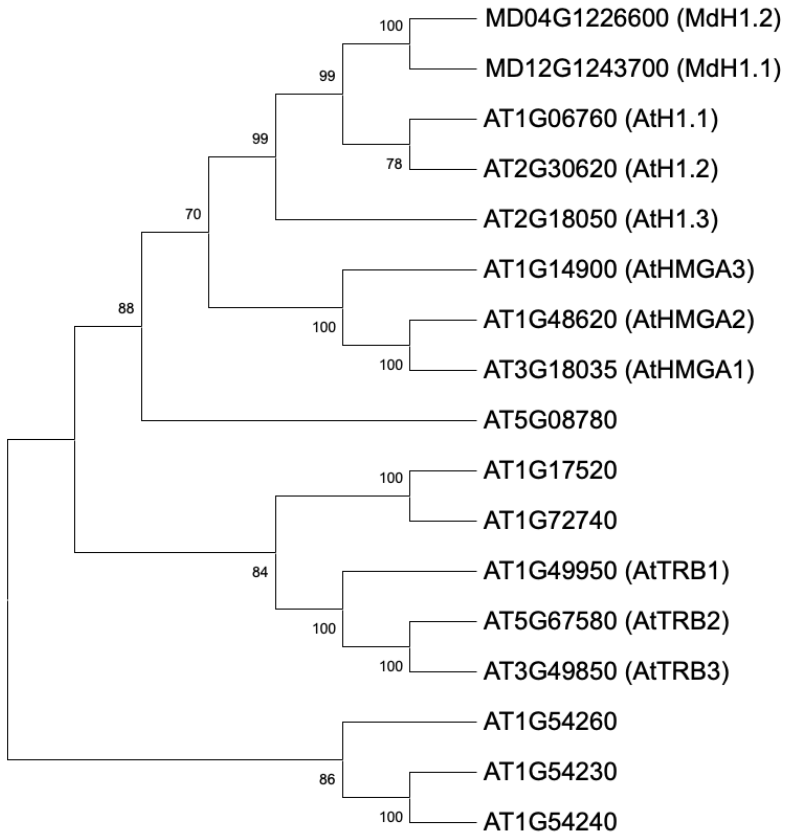

Supplementary Figure S4. Phylogenetic analysis of apple and Arabidopsis histone H1 globular (GH1)-domain proteins. MdH1.1, MdH1.2, and 15 Arabidopsis GH1-domain proteins including 3 canonical H1 variants (AtH1.1, AtH1.2 and AtH1.3) were aligned using ClustalW. The phylogenetic tree was constructed using the MEGA 11.0 program by the neighbor-joining method with 1000 bootstrap iterations. HMGA: High Mobility Group A; TRB: Telomere Repeat Binding Factor (Kotliński et al., 2017).

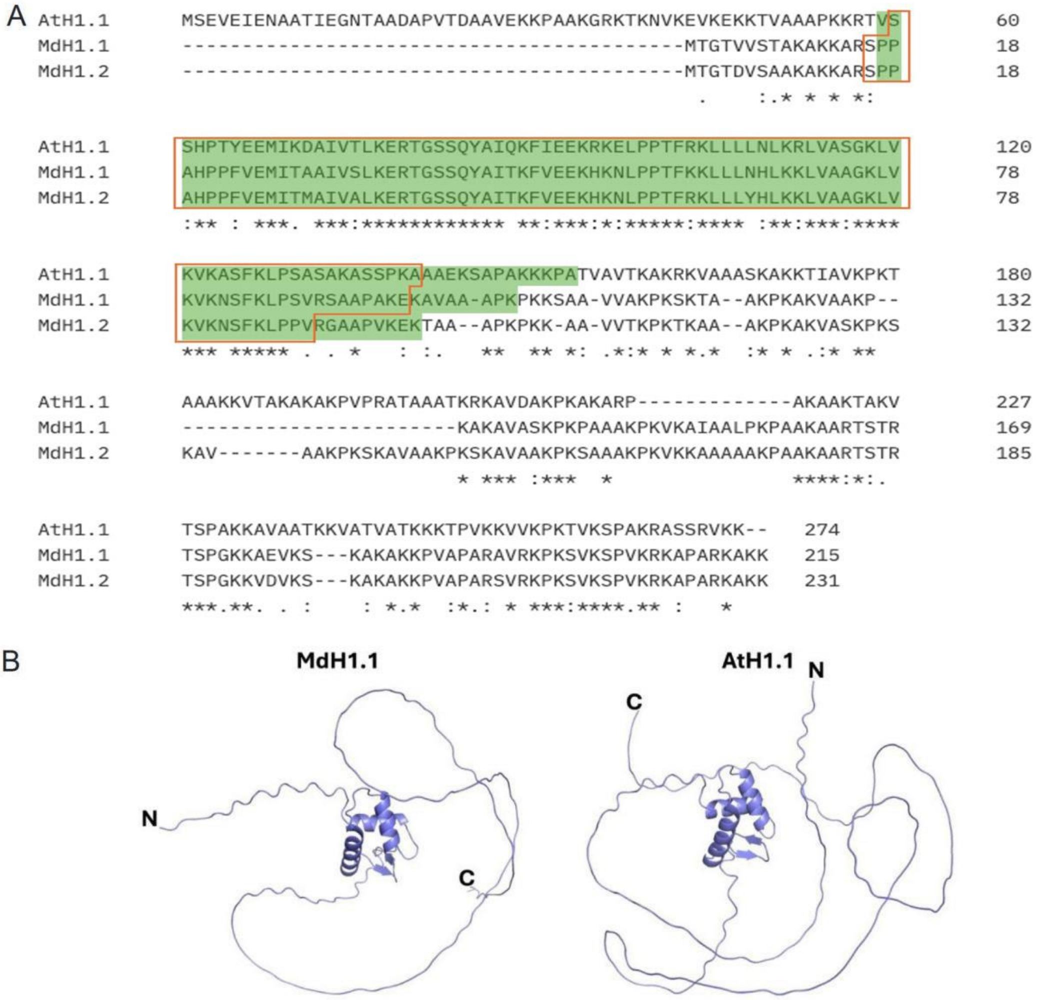

Supplementary Figure S5. Conserved domain analysis of apple H1.1, H1.2 and Arabidopsis H1.1 and predicted structures.

(A) AtH1.1, MdH1.1 and MdH1.2 conserved domain prediction based on InterPro Scan results (https://www.ebi.ac.uk/interpro/). Green block indicates the linker histone H1/H5 domain (IPR005818), with amino acids 59-153 for AtH1.1, 17-104 for MdH1.1 and 17-98 for MdH1.2. Orange frame denotes the conserved winged-helix DNA binding domain (IPR036390) of the winged-helix DNA binding protein superfamily, with amino acids 60-140 for AtH1.1, 16-97 for MdH1.1 and 16-89 for MdH1.2. The symbols * means the same amino acid residue for AtH1.1, MdH1.1 and MdH1.2; the symbols : and . indicate that two of the three amino acid residues for AtH1.1, MdH1.1 and MdH1.2 are identical.

(B) MdH1.1 and AtH1.1 protein structures predicted by Alphafold2 (https://neurosnap.ai/service/AlphaFold2) (Jumper et al., 2021). The graphs were generated by PyMOL (Version 3.0.3, Schrödinger, LLC.). Both proteins have a

conserved H1 globular domain containing three α-helixes and two β-sheets. MdH1.1 has a much shorter N-terminus than AtH1.1.

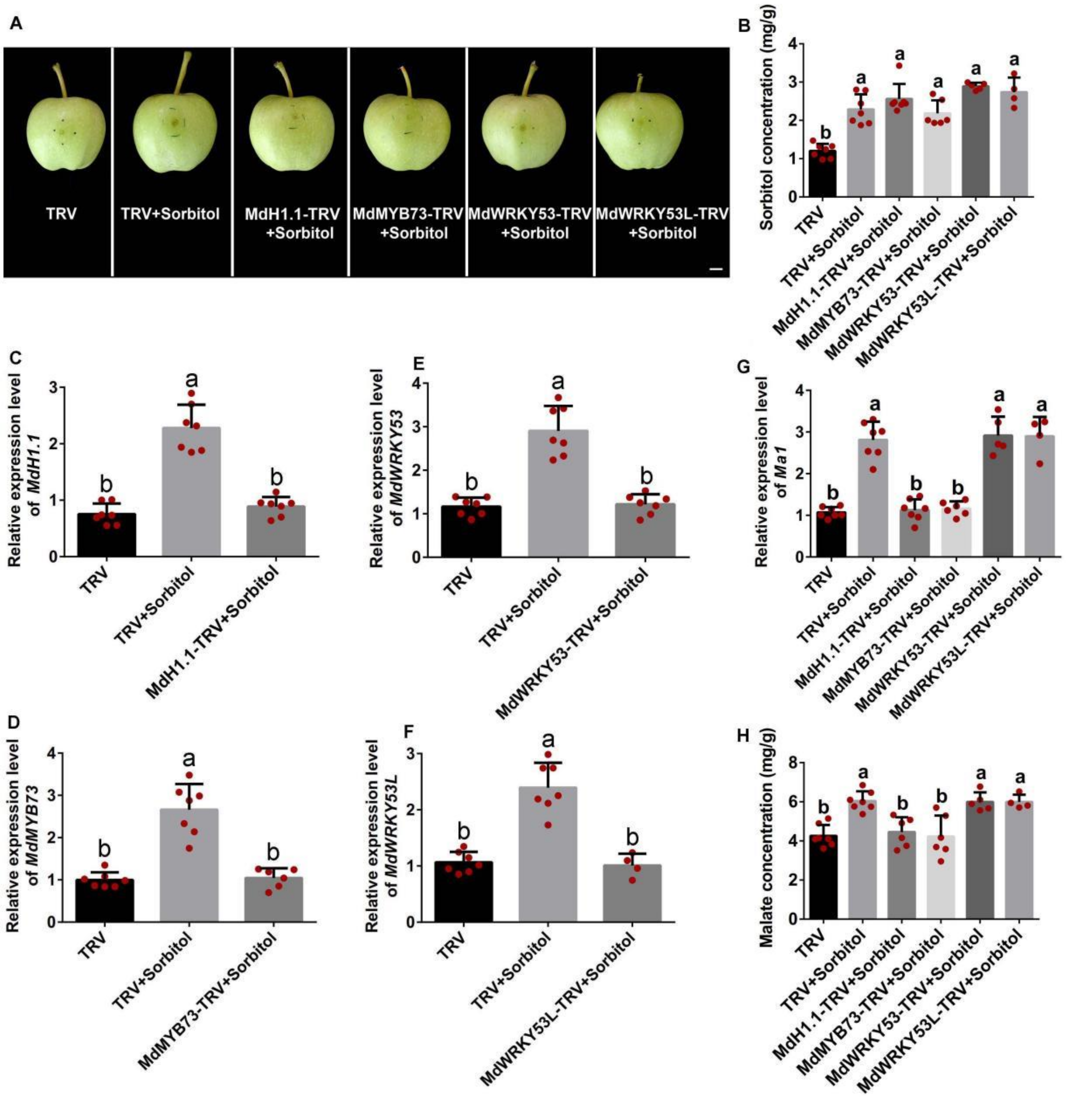

Supplementary Figure S6. Functional testing of MdH1.1, MdMYB73, MdWRKY53, and MdWRKY53L for their involvement in sorbitol-mediated malate accumulation.

(A) Transient suppression of *MdH1.1*, *MdMYB73*, *MdWRKY53*, and *MdWRKY53L* by the viral vector-based agro-infiltration in combination with or without 50 mM sorbitol in apple fruit. The antisense cDNA fragments of *MdH1.1*, *MdMYB73*, *MdWRKY53*, and *MdWRKY53L* were inserted into the TRV vector for suppression, respectively, with the empty vector as control. All images are of the same scale. Bar = 1 cm.

(B) Sorbitol concentrations of the agro-infiltrated fruit as indicated in **(A)**.

(C-G) RT-qPCR analysis of the expression levels of *MdH1.1* **(C)**, *MdMYB73* **(D)**, *MdWRKY53* **(E)**, and *MdWRKY53L* **(F)** and *Ma1* **(G)** in the cortex tissues around the injection site as indicated in **(A)**, respectively. The relative expression level of each gene was obtained via RT-qPCR using the ddCT method, with *ACTIN* as a reference.

(H) Malate concentrations of the agro-infiltrated fruit as indicated in **(A)**.

In **(B)** to **(H)**, data are mean ± SE of 4 to 7 biological replicates with 3 fruits per replicate.

Different letters indicate significant difference at $P < 0.05$ using Tukey's HSD test after ANOVA.

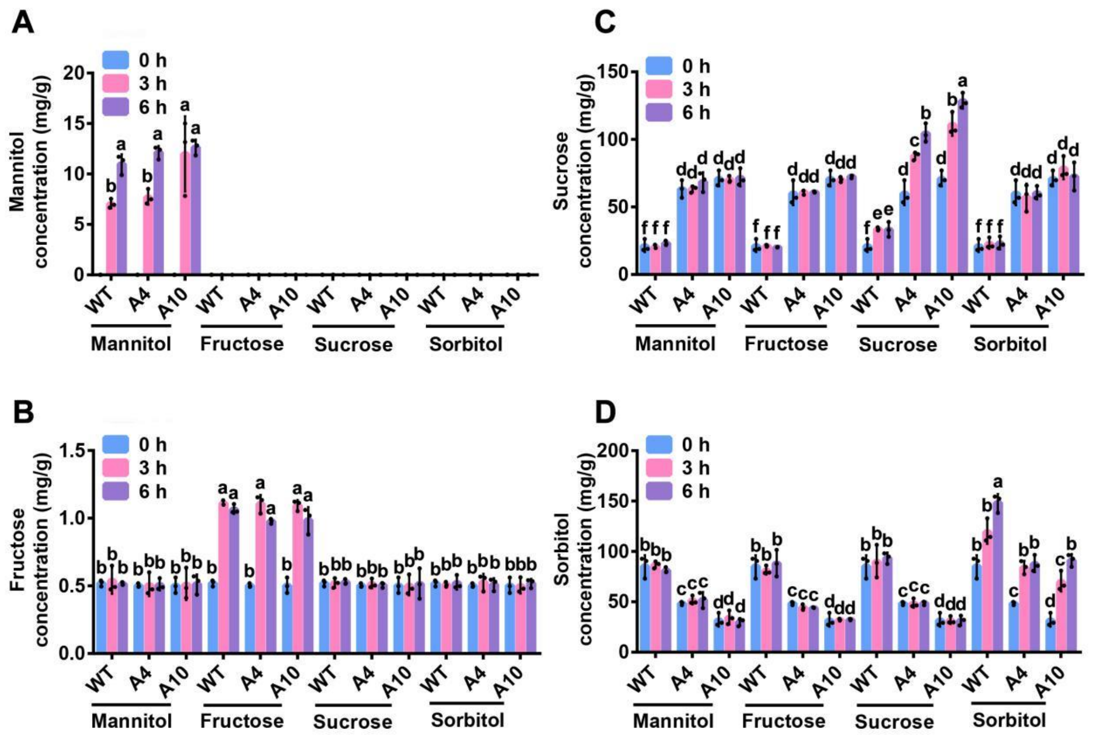

Supplementary Figure S7. The concentrations of mannitol, fructose, sucrose, and sorbitol in fully expanded leaves of the WT control and *A6PR* antisense lines (A4 and A10) in response to sugar feeding. Leaves were fed with 50 mM of mannitol, fructose, sucrose, or sorbitol via the leaf petioles. Concentrations of mannitol **(A)**, fructose **(B)**, sucrose **(C)**, and sorbitol **(D)** in the leaves were measured with GC/MS. Data are mean ± SE of 3 biological replicates with 3 leaves pooled from 3 shoots per replicate. Different letters indicate significant difference at $P < 0.05$ using Tukey's HSD test after ANOVA.

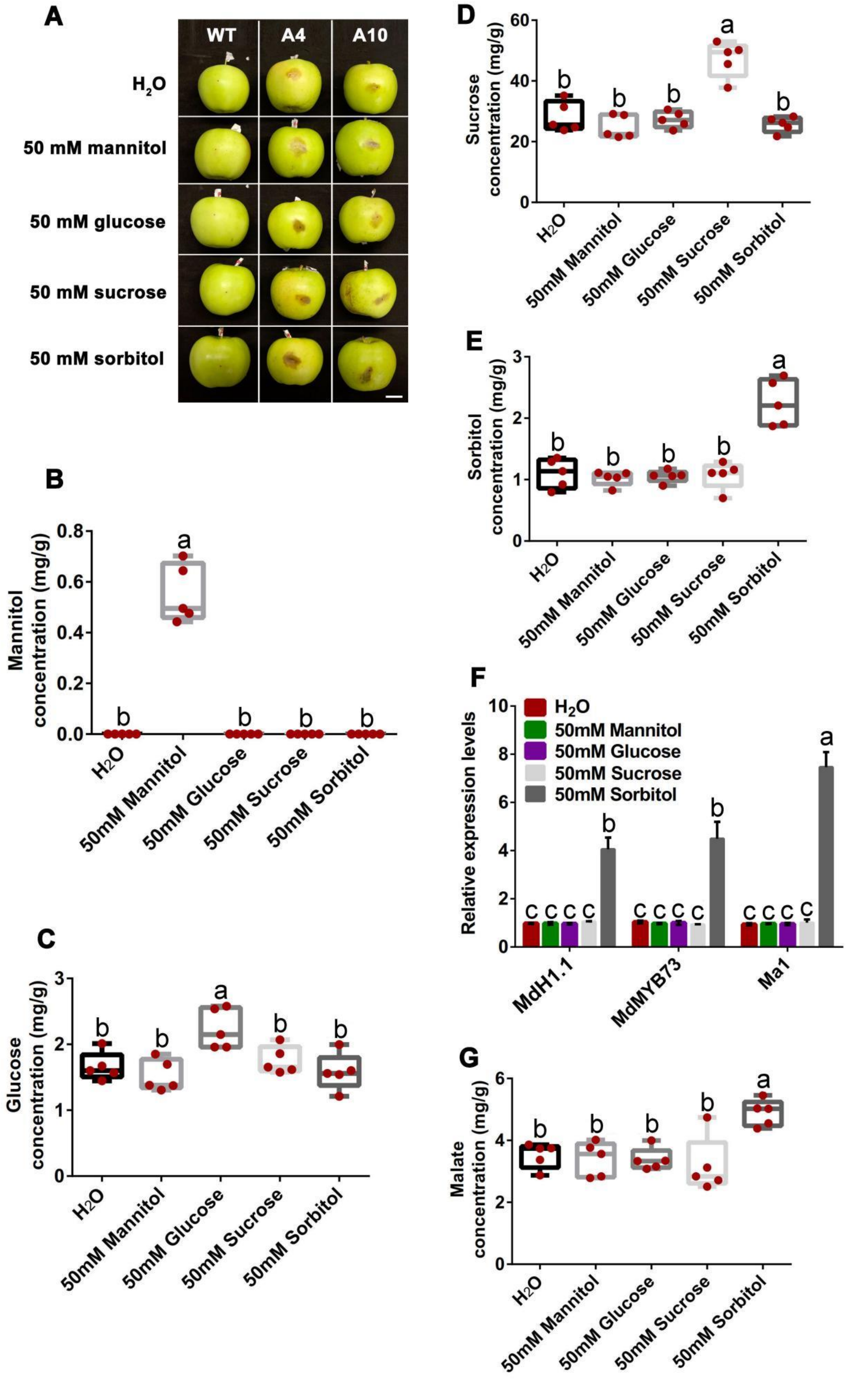

Supplementary Figure S8. The fruits of WT and *A6PR* antisense lines A4 and A10

injected with various sugars at a concentration of 50 mM.

(A) The phenotypes of the fruits of WT and two antisense lines A4 and A10 after injection with 250 µL of 50 mM mannitol, glucose, sucrose, or sorbitol, or with H_2O. All images are of the same scale. Bar = 2 cm.

(B-E) Mannitol **(B)**, Glucose **(C)**, Sucrose **(D)**, and Sorbitol **(E)** concentrations of the WT fruit in response to sugar injection as indicated in **(A)**.

(F) The relative expression levels of *MdH1.1*, *MdMYB73*, and *Ma1* in the WT fruit in response to sugar injection as indicated in **(A)**. Data are mean ± SE 5 biological replicates with 3 fruits per replicate. Different letters indicate significant difference at $P < 0.05$ using Tukey's HSD test after ANOVA.

(G) Malate concentrations in the WT fruit in response to sugar injection as indicated in **(A)**.

In **(B)** to **(E)** and **(G)**, data are obtained from 5 biological replicates with 3 fruits per replicate. The boxes represent interquartile ranges, with the middle lines as medians and the whiskers as the maximum and minimum values. Different letters indicate significant difference at $P < 0.05$ using Tukey's HSD test after ANOVA.

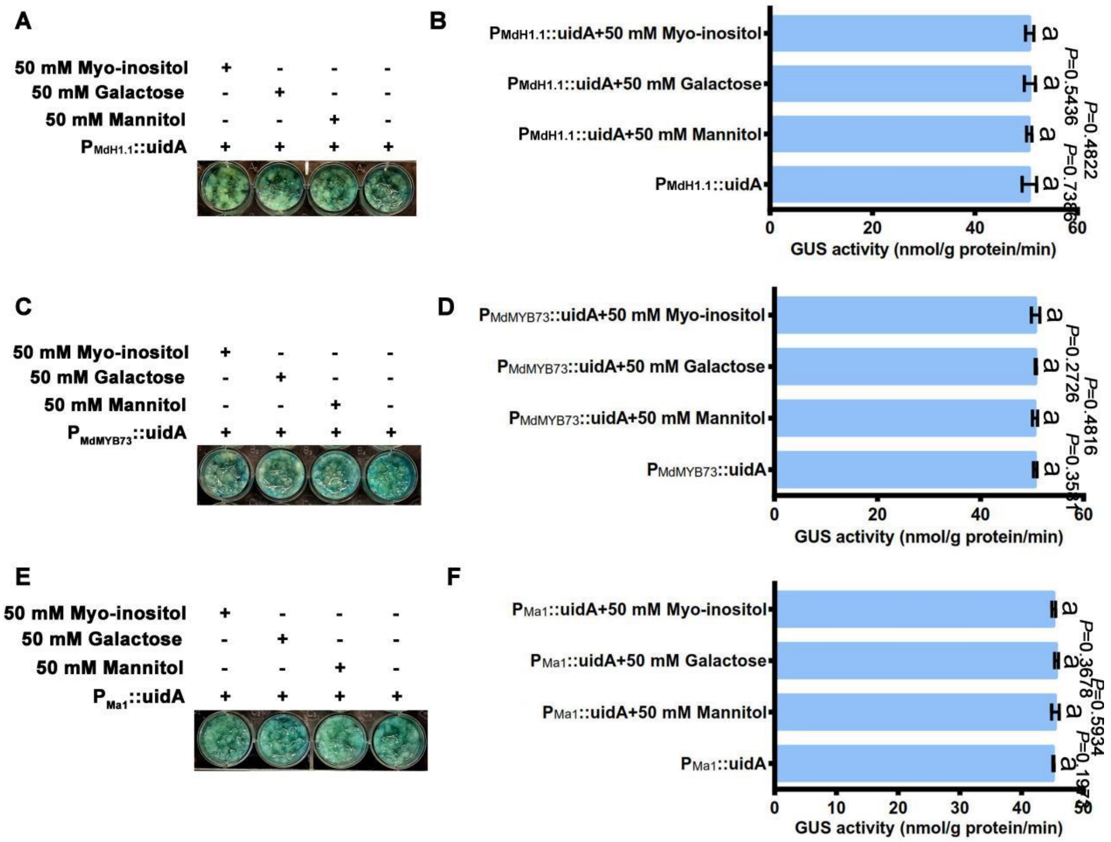

Supplementary Figure S9. The expression of *MdH1.1*, *MdMYB73*, or *Ma1* is not induced by mannitol, galactose or myo-inositol.

(A) and **(B)** GUS assays of $P_{MdH1.1}$::*uidA* transgenic apple calli treated with mannitol, galactose, or myo-inositol at a concentration of 50 mM. The $P_{MdH1.1}$::*uidA* reporter construct in the binary vector was introduced into apple calli. The $P_{MdH1.1}$::*uidA* transgenic apple calli were treated with 50 mM mannitol, 50 mM galactose, or 50 mM myo-inositol for 12 hours at 25°C in the dark, and were stained to visualize GUS activity. The $P_{MdH1.1}$::*uidA* transgenic apple calli without sugar treatment was used as the control.

(C) and **(D)** GUS assays of $P_{MdMYB73}$::*uidA* transgenic apple calli treated with mannitol, galactose, or myo-inositol at a concentration of 50 mM. The $P_{MdMYB73}$::*uidA* reporter construct in the binary vector was introduced into apple calli. The $P_{MdMYB73}$::*uidA* transgenic apple calli were treated with 50 mM mannitol, 50 mM galactose, or 50 mM myo-inositol for 12 hours at 25°C in the dark, and were stained to visualize GUS activity.

(E) and **(F)** GUS assays of P_{Ma1}::*uidA* transgenic apple calli treated with mannitol, galactose, or myo-inositol at a concentration of 50 mM. The P_{Ma1}::*uidA* reporter construct in the binary vector was introduced into apple calli. The P_{Ma1}::*uidA* transgenic apple calli were treated with 50 mM mannitol, 50 mM galactose, or 50 mM myo-inositol for 12 hours at 25°C in the dark, and were stained to visualize GUS activity.

In **(B)**, **(D)**, **(F)**, data are mean ± SE of 3 biological replicates with calli grown in one petri dish as a replicate. The same letter (a) indicates no significant difference was detected with ANOVA.

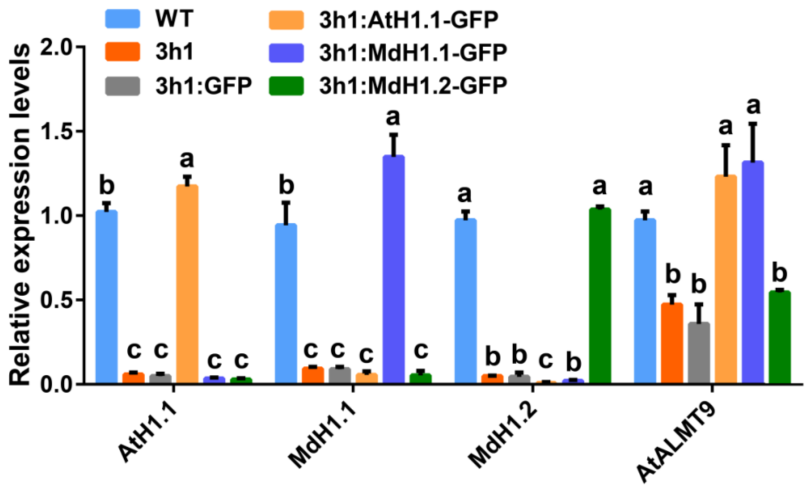

Supplementary Figure S10. The expression levels of *AtH1.1*, *MdH1.1*, *MdH1.2*, and *AtALMT9* in Arabidopsis seedlings of wild-type (WT), H1 triple mutant *3h1*, and *3h1* complemented with GFP, AtH1.1-GFP, MdH1.1-GFP or MdH1.2-GFP. *3h1*:GFP, *3h1*:AtH1.1-GFP, *3h1*:MdH1.1-GFP and *3h1*:MdH1.2-GFP represent complementation of Arabidopsis *3h1* with empty vector GFP, Arabidopsis AtH1.1-GFP, apple MdH1.1-GFP or MdH1.2-GFP driven by the *RPS5a* promoter. The relative expression level of each gene was obtained via RT-qPCR using the ddCT method, with *ACTIN* as a reference. Data are mean ± SE of 4 biological replicates with 3 plants per replicate. Different letters (a, b, and c) indicate significant differences between groups using Tukey's HSD test at $P < 0.05$ after ANOVA.

MdMYB73 Biotin Probe 1 (Promoter -184~-243 bp): CATGA-
CAGCTCACATTGTCTCAATATATCGGGGGAAGCCCGAAATG-
GTAAATGAACAAAA

MdMYB73 Biotin Probe 2 (Promoter -36~-95 bp): AAGTTAG-
CAAACCCGCCCACTTTACAAACACCAGTTTCGTGGAAG-
TACCCTTCTCCTCTC

MdtDT Biotin Probe 1 (Promoter -647~-706 bp): AGGGTTGC-
CAATTAAGGCAGCTCTCATACCAGGTGCGATTAAAATACAG-
TAATCACATAT

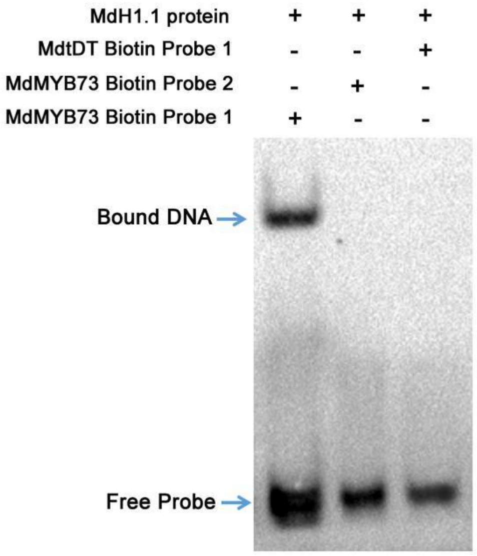

Supplementary Figure S11. EMSA assays show no interaction between MdH1.1 and biotin-labeled DNA probes for the *MdMYB73* promoter without a WHD binding *cis*-element or the *MdtDT* promoter containing a putative WHD binding *cis*-element but MdH1.1 does not bind to. The interaction between MdH1.1 protein and *MdMYB73* biotin probe 1 containing a WHD binding *cis*-element was used as a positive control.

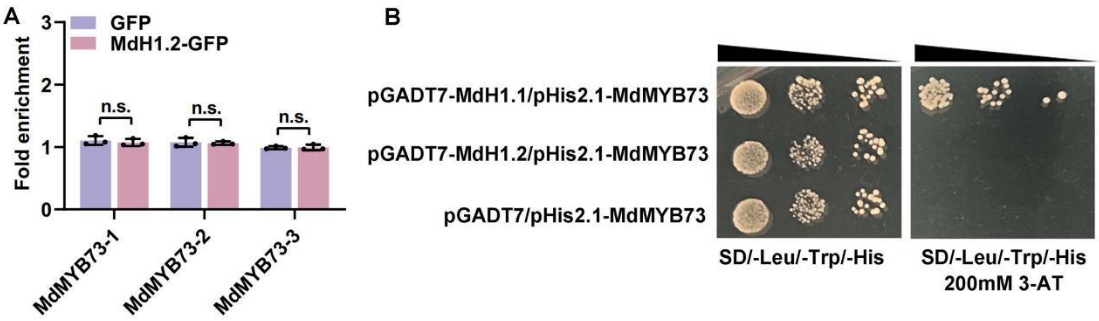

Supplementary Figure S12. MdH1.2 does not bind to the promoter of *MdMYB73*.

(A) ChIP-qPCR assays of the enrichment of the *MdMYB73* promoter fragments in *35S::MdH1.2-GFP* transgenic apple calli relative to *35S::GFP* transgenic apple calli. The MdH1.2-DNA complex was co-immunoprecipitated from *35S::GFP* and *35S::MdH1.2-GFP* transgenic apple calli using a GFP antibody. Data are mean ± SE of 5 biological replicates with calli grown in one petri dish as one replicate. Statistical difference was determined using Student's *t*-test at $P < 0.05$ against the GFP empty vector control at an enrichment value designated as 1. n.s. means non-significant.

(B) Y1H assays showing that MdH1.2 does not bind to the promoter fragments of *MdMYB73*, containing the WHD-binding motifs. The basal concentration of 3-AT at 200 mM was used to screen interacting fragments. pGADT7-MdH1.1 combined with the *MdMYB73* promoter (pGADT7-MdH1.1/pHis2.1-MdMYB73) was used as a positive control whereas the empty vector pGADT7 combined with the *MdMYB73* promoter (pGADT7/pHis2.1-MdMYB73) was used as a negative control. The yeast cells were spotted with serial dilutions (1/1, 1/10, 1/100) onto the yeast medium.

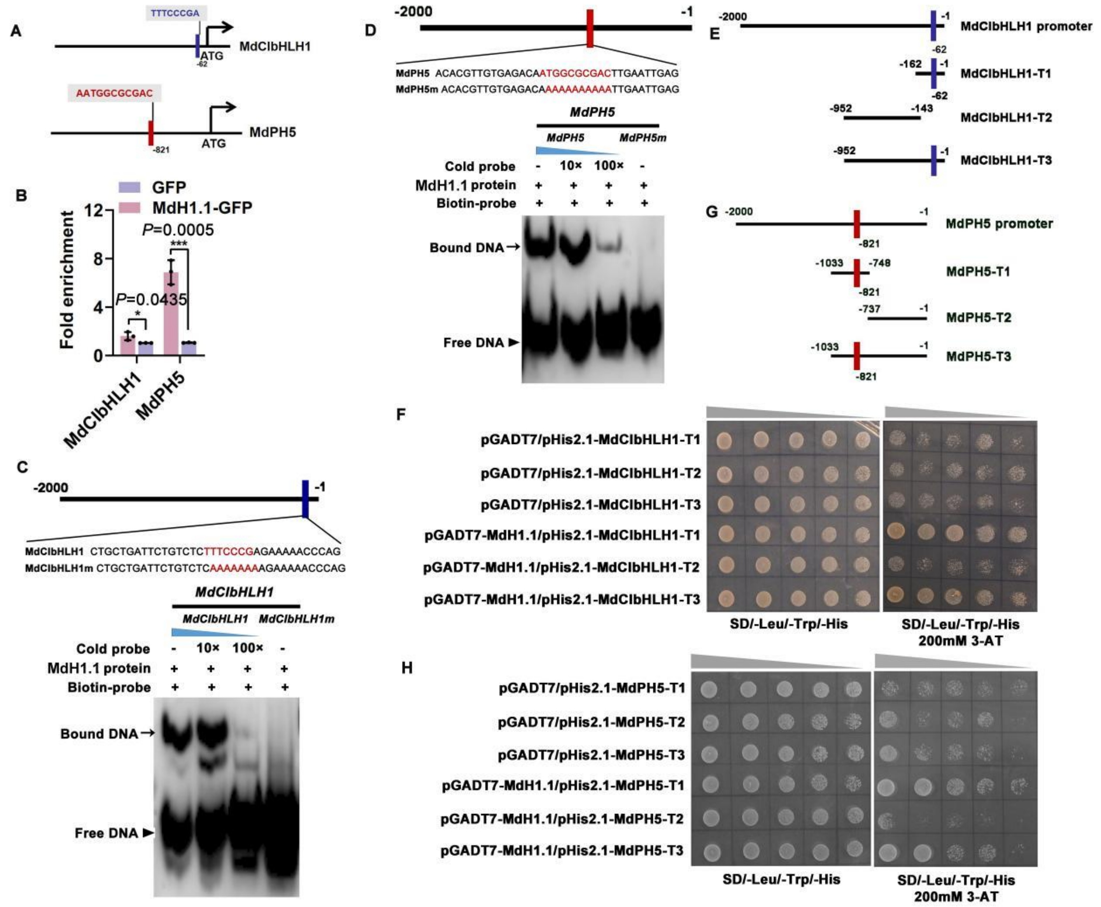

Supplementary Figure S13. MdH1.1 binds to the promoters of *MdCIbHLH1* and *MdPH5*.

(A) The putative WHD-binding element on the promoters of *MdCIbHLH1* and *MdPH5*. The upper diagram shows a WHD-binding element in the promoter of *MdCIbHLH1*. The bottom diagram indicates a WHD-binding element in the promoter of *MdPH5*. The blue and red rectangles denote the positions of the WHD-binding motif in the promoters of *MdCIbHLH1* and *MdPH5*, respectively.

(B) ChIP-qPCR assays of the enrichment of the promoters of *MdCIbHLH1* and *MdPH5* in *35S::MdH1.1-GFP* transgenic apple calli relative to *35S::GFP* transgenic apple calli. The MdH1.1-DNA complex was co-immunoprecipitated from *35S::GFP* and *35S::MdH1.1-GFP* transgenic apple calli using an anti-GFP antibody. Data are mean ± SE of 5 biological replicates with calli grown in one petri dish as a replicate. Statistical significance was determined using Student's *t*-test at $^{*}P < 0.05$ or $^{***}P < 0.001$ against the GFP empty vector control at an enrichment value designated as 1.

(C) Electrophoretic mobility shift assays (EMSAs) of the interaction between MdH1.1 and labeled DNA probes in the promoter of *MdCIbHLH1*. Lane 1 shows the labeled

DNA probe and the MdH1.1 protein without a competitor. Increasing amounts (10× and 100×) of the unlabeled DNA fragments (MdCIbHLH1 in lanes 2 and 3) were added as cold competitors. Lane 4 shows the labeled mutant DNA probe.

(D) EMSAs of the interaction between MdH1.1 and labeled DNA probes in the promoter of *MdPH5*. Lane 1 shows the labeled DNA probe and the MdH1.1 protein without a competitor. Increasing amounts (10× and 100×) of the unlabeled DNA fragments (MdPH5 in lanes 2 and 3) were added as cold competitors. Lane 4 shows the labeled mutant DNA probes.

(E) Schematic representation of the different truncations of the *MdCIbHLH1* promoter in yeast vectors. The *MdCIbHLH1* promoter was divided into three fragments according to the position of the putative WHD-binding element.

(F) Yeast one hybrid (Y1H) assays showing that MdH1.1 binds to the promoter fragments of *MdCIbHLH1* containing the WHD-binding motif. The basal concentration of 3-AT was 200 mM was used for screening interacted fragments. The empty vector combined with the different promoter fragments of *MdCIbHLH1* (pGADT7/pHis2.1-MdCIbHLH1-T1~T3) were used as negative controls. The yeast cells were spotted with serial dilutions (1/1, 1/10, 1/100, 1/1,000, 1/10,000) onto the yeast medium.

(G) Schematic representation of the different truncations of the *MdPH5* promoter in yeast vectors. The *MdPH5* promoter was divided into three deletions according to the position of the putative WHD-binding element.

(H) Y1H assays showing that MdH1.1 binds to the promoter fragments of *MdPH5*, containing the WHD-binding motif. The basal concentration of 3-AT was 200 mM was used for screening interacted fragments. The empty vector combined with the different promoter fragments of *MdPH5* (pGADT7/pHis2.1-MdPH5-T1~T3) were used as negative controls. The yeast cells were spotted with serial dilutions (1/1, 1/10, 1/100, 1/1,000, 1/10,000) onto the yeast medium.

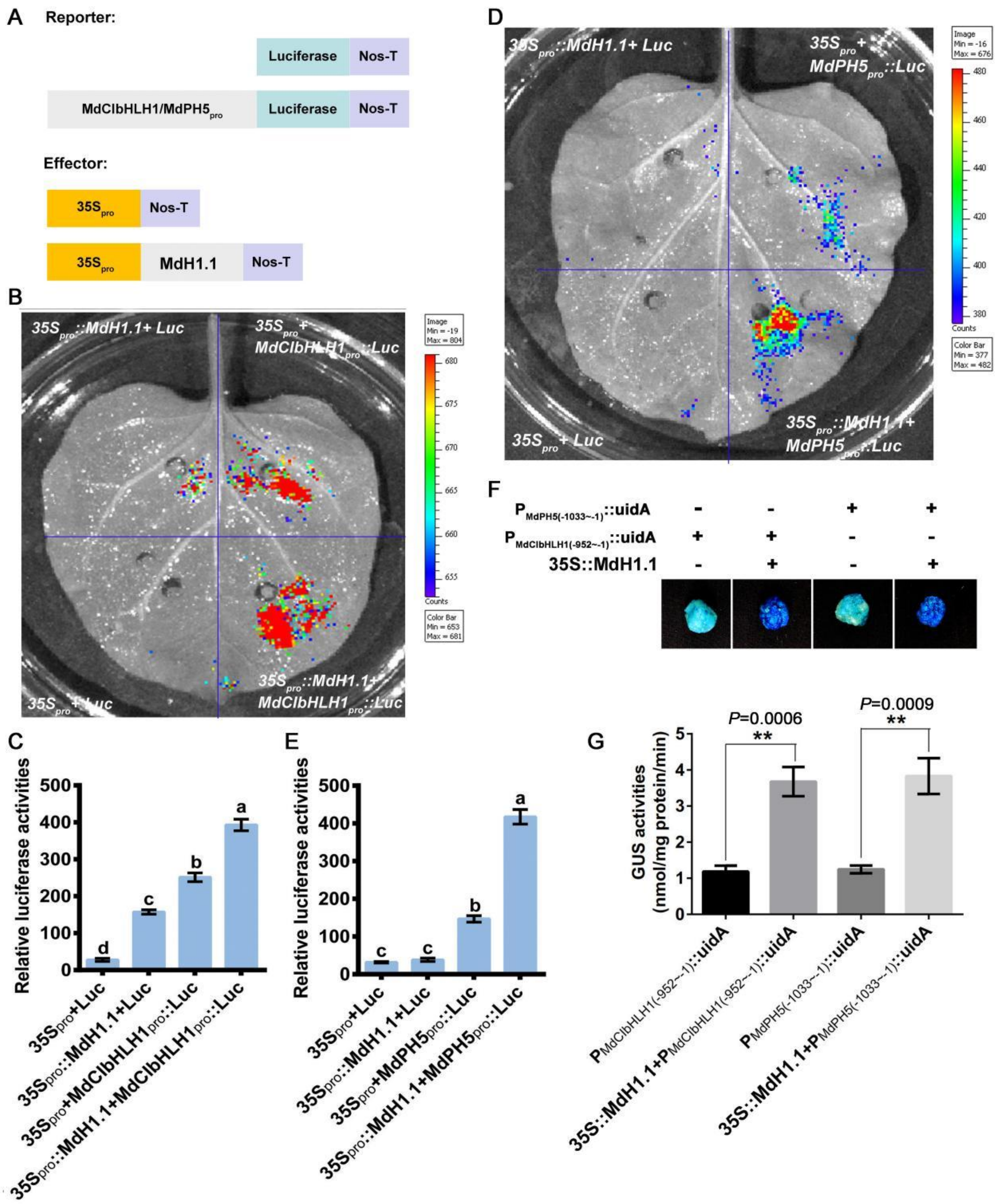

Supplementary Figure S14. MdH1.1 activates the transcription of *MdCIbHLH1* and *MdPH5*.

(A) Schematic representation of the Luc reporter vectors containing the promoters of *MdCIbHLH1* (-952~-1) and *MdPH5* (-1033~-1), and the effector vector containing MdH1.1.

(B) Transient expression assays showing that MdH1.1 activates the expression of *MdCIbHLH1*$_{pro}$*::Luc*. Representative images of *Nicotiana benthamiana* leaves 72 h after infiltration were shown.

(C) Quantitative analysis of luminescence intensity as indicated in **(B)**. The value for

luminescence intensity in *$35S_{pro}$+Luc* samples was taken as the negative control.

(D) Transient expression assays showing that MdH1.1 activates the expression of *$MdPH5_{pro}$::Luc*. Representative images of *N. benthamiana* leaves at 72 h after infiltration were shown.

(E) Quantitative analysis of luminescence intensity as indicated in **(D)**. The value for luminescence intensity in *$35S_{pro}$+Luc* samples was used as the negative control.

(F) The effector and reporter constructs in the binary vectors were introduced into apple calli for GUS activity assays. *$P_{MdClbHLH1(-952\sim-1)}$::uidA* and *$P_{MdPH5(-1033\sim-1)}$::udiA* transgenic apple calli with or without the *35S::MdH1.1* effector, were grown at 25°C in the dark and were stained to visualize GUS activity.

(G) GUS activities as indicated in **(F)** were measured. Data are mean ± SE of 3 biological replicates with calli grown in one petri dish as a replicate. Statistical significance was determined using Student's *t*-test at $^{**}P < 0.001$.

In **(C)** and **(E)**, data are mean ± SE of 3 biological replicates with 3 leaves per replicate. Different letters indicate significant differences between groups using Tukey's HSD test at $P < 0.05$ after ANOVA.

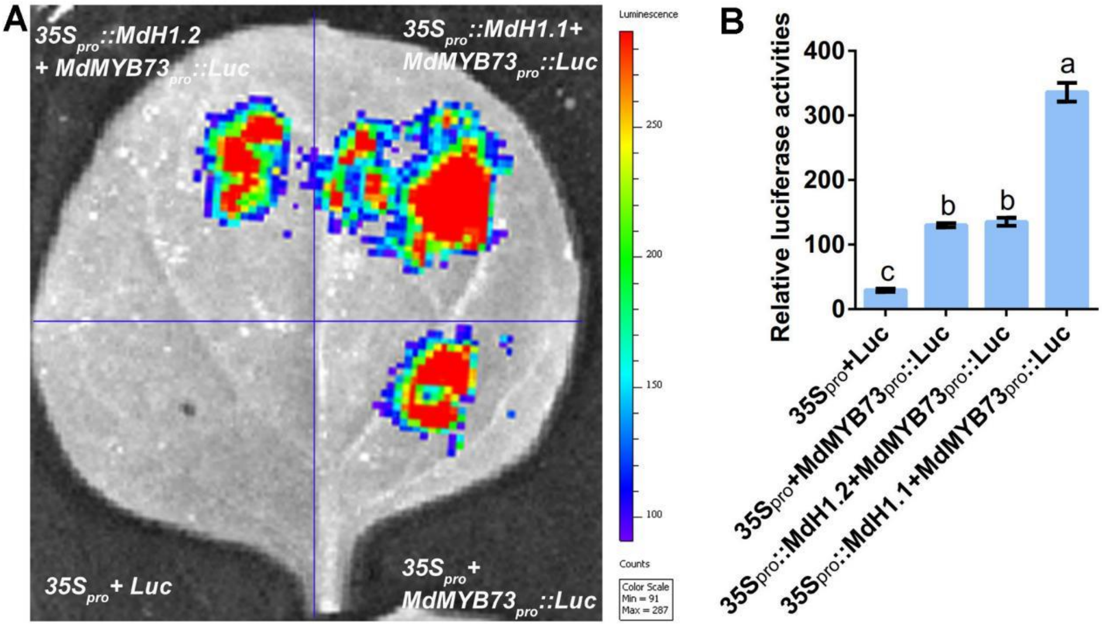

Supplementary Figure S15. MdH1.2 does not activate the transcription of *MdMYB73*.

(A) Transient expression assays showing that MdH1.2 does not activate the expression of *MdMYB73$_{pro}$::Luc*. The activation of MdH1.1 on *MdMYB73$_{pro}$::Luc* was used as the positive control. Representative images of *Nicotiana benthamiana* leaves at 72 h after infiltration were shown.

(B) Quantitative analysis of luminescence intensity as indicated in **(A)**. The value for luminescence intensity in *35S$_{pro}$+Luc* samples was taken as the negative control. Data are mean ± SE of 3 biological replicates with 3 leaves per replicate. Different letters indicate significant differences between groups using Tukey's HSD test at P < 0.05 after ANOVA.

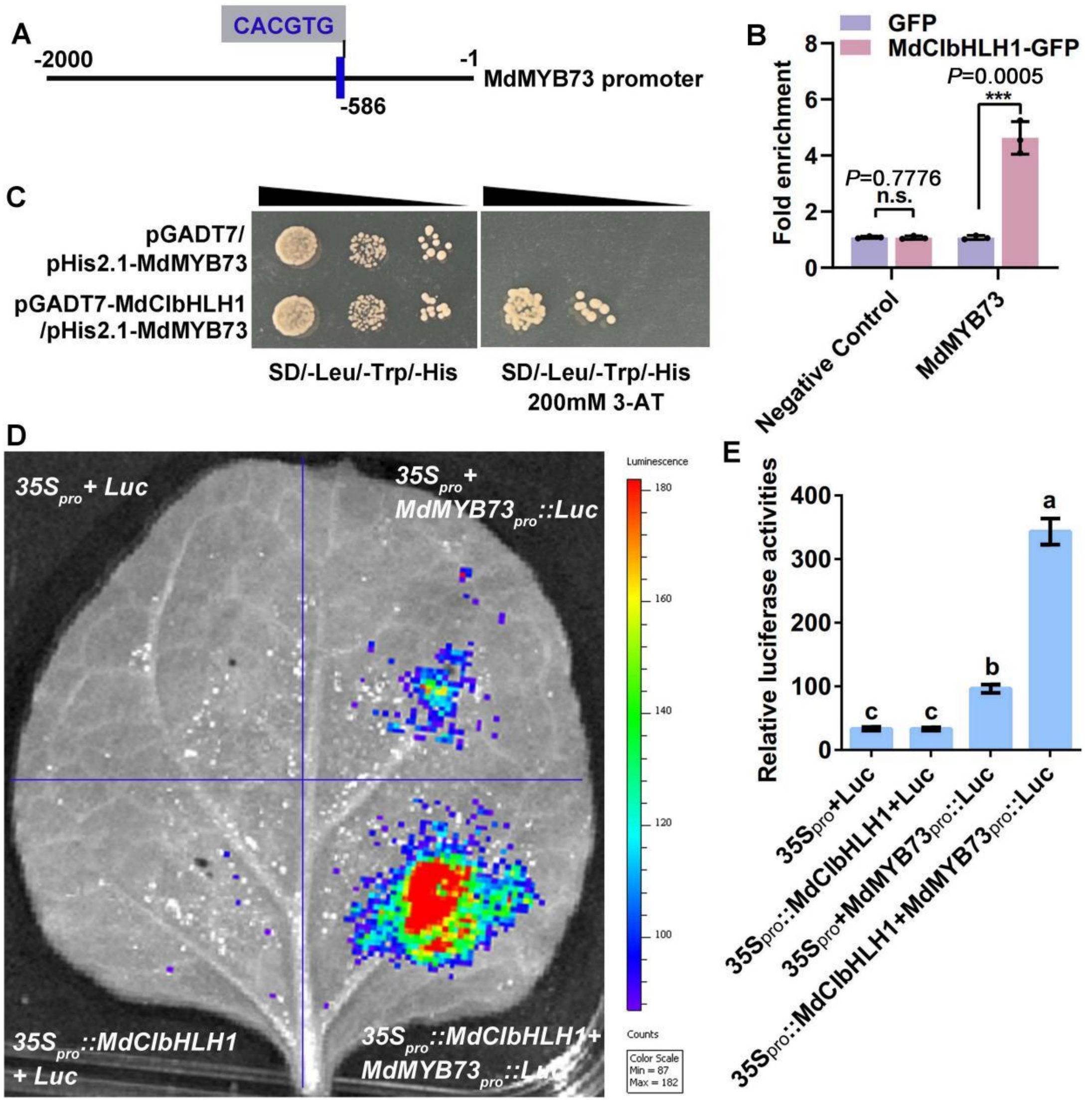

Supplementary Figure S16. MdClbHLH1 binds to the promoter of *MdMYB73*, activating its transcription.

(A) The putative bHLH-binding element (G-box) in the promoter of *MdMYB73*. The blue bar denotes the position of the bHLH-binding motif in the promoter of *MdMYB73*.

(B) ChIP-qPCR assays of the enrichment of the *MdMYB73* promoter in *35S::MdClbHLH1-GFP* transgenic apple calli relative to *35S::GFP* transgenic apple calli. The MdClbHLH1-DNA complex was co-immunoprecipitated from *35S::GFP* and *35S::MdClbHLH1-GFP* transgenic apple calli using a GFP antibody. The promoter region of *MdMYB1* without a WHD-binding *cis*-element was used as the negative control. Data are mean ± SE of 5 biological replicates with calli grown in one petri dish as one replicate. Statistical difference was determined using Student's *t*-test at $P < 0.001$ against the GFP empty vector control at an enrichment value designated as 1.

(C) Y1H assays showing that MdClbHLH1 binds to the *MdMYB73* promoter containing

the bHLH-binding motifs. The basal concentration of 3-AT was 200 mM. The empty vector and the *MdMYB73* promoter (pGADT7/pHis2.1-MdMYB73) were used as negative control. The yeast cells were spotted with serial dilutions (1/1, 1/10, 1/100) onto the yeast medium.

(D) Transient expression assays showing that MdCIbHLH1 activates the expression of $MdMYB73_{pro}$*::Luc*. Representative images of *Nicotiana benthamiana* leaves 72 h after infiltration were shown.

(E) Quantitative analysis of luminescence intensity as indicated in **(D)**. The value for luminescence intensity in $35S_{pro}$*+Luc* samples was taken as the negative control. Data are mean ± SE of 3 biological replicates with 3 leaves per replicate. Different letters indicate significant differences between groups using Tukey's HSD test at $P < 0.05$ after ANOVA.

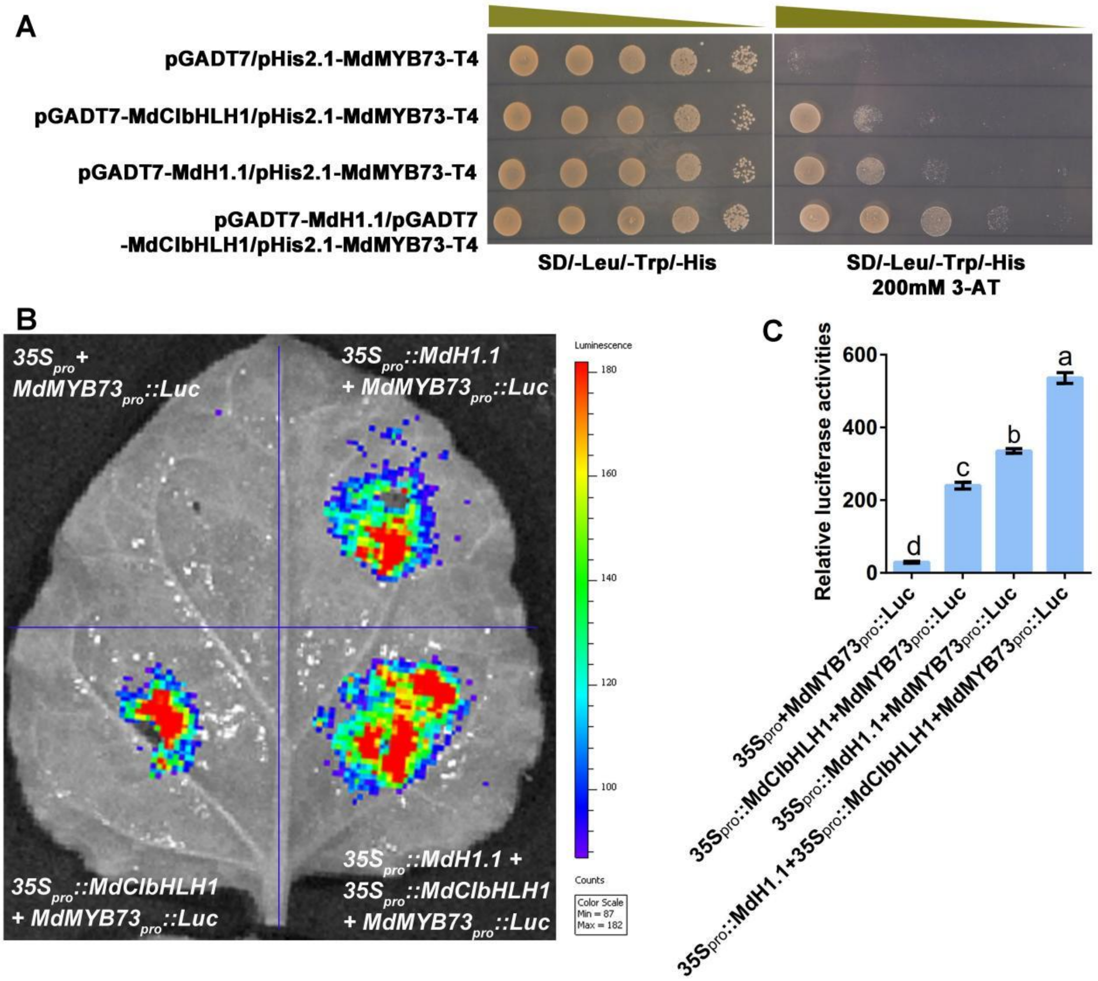

Supplementary Figure S17. Yeast one-hybrid (Y1H) and dual luciferase (Luc) assays showing that MdH1.1 together with MdClbHLH1 enhance the transcription activity of *MdMYB73*. The basal concentration of 3-AT at 200 mM was used for screening interactions. The empty vector combined with the promoter of *MdMYB73* (pGADT7/pHis2.1-MdMYB73-T4) was used as a negative control in **(A)**. The presence of both pGADT7-MdH1.1 and pGADT7-MdClbHLH1 in the pGADT7-MdH1.1/pGADT7-MdClbHLH1/pHis2.1-MdMYB73-T4 combination was confirmed with PCR. The yeast cells were spotted with serial dilutions (1/1, 1/10, 1/100, 1/1,000, 1/10,000) onto the yeast medium. Representative images of *Nicotiana benthamiana* leaves 72 h after infiltration were shown in **(B)**. **(C)** Quantitative analysis of luminescence intensity as indicated in **(B)**. Data are mean ± SE of 3 biological replicates with 3 leaves per replicate. Different letters indicate significant difference using Tukey's Honestly Significant Difference test at $P < 0.05$ after ANOVA.

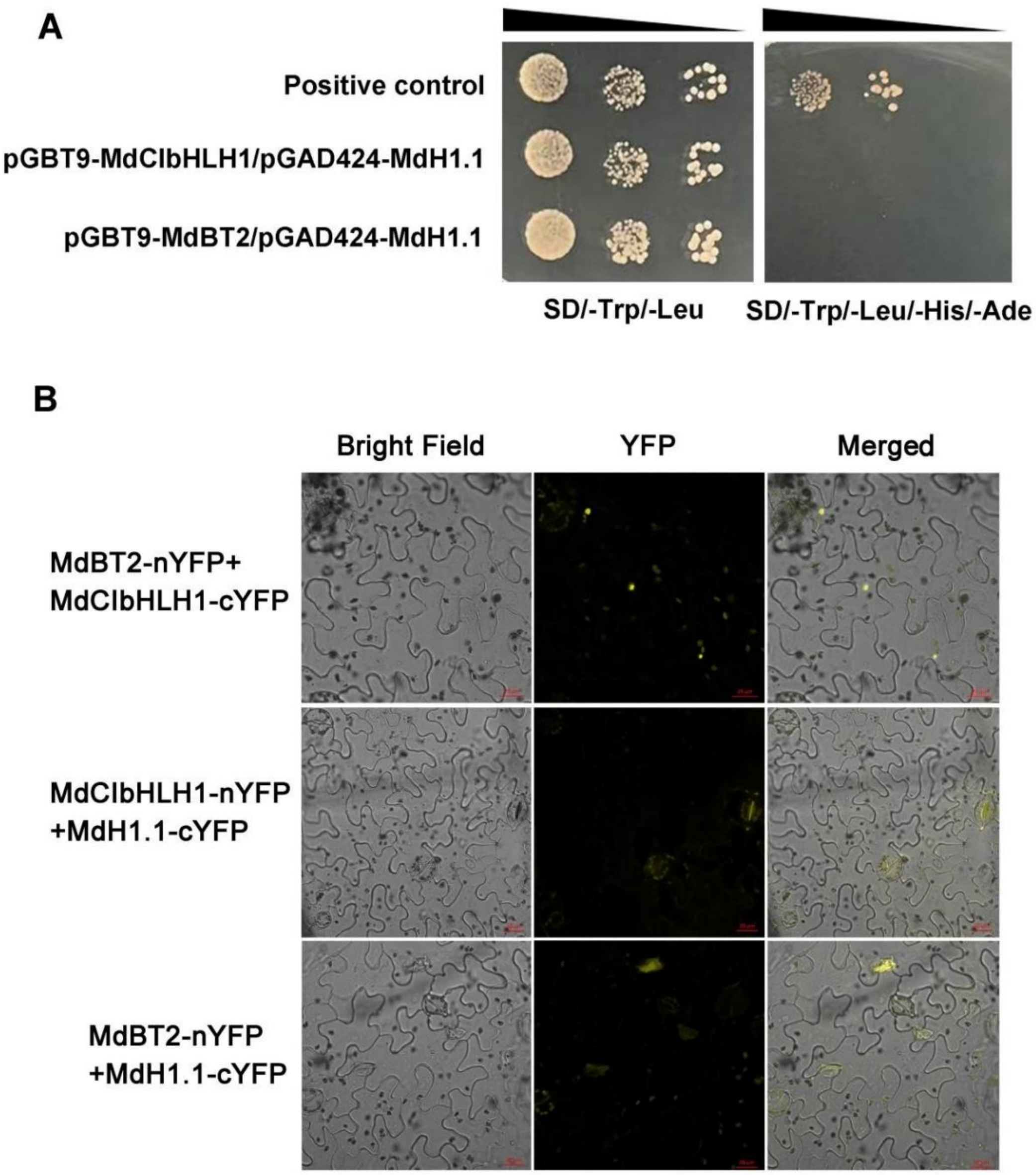

Supplementary Figure S18. No interaction detected between MdH1.1 and MdCIbHLH1 or MdBT2 proteins.

(A) Y2H assays show no interaction between MdH1.1 and MdCIbHLH1 or MdBT2 proteins. The interaction between pGBT9-MdCIbHLH1 and pGAD424-MdMYB73 characterized earlier (Hu et al., 2017) was used as a positive control. The yeast cells were spotted with serial dilutions (1/1, 1/10, 1/100) onto the yeast medium.

(B) BiFC assays showing no interaction between MdH1.1 and MdCIbHLH1 or MdBT2 proteins in *Nicotiana benthamiana* leaf cells. The interaction between MdBT2-nYFP and MdCIbHLH1-cYFP in the nucleus of tobacco leaf cells (Zhang et al., 2020a) was used as a positive control. Scale bar = 20 μm.

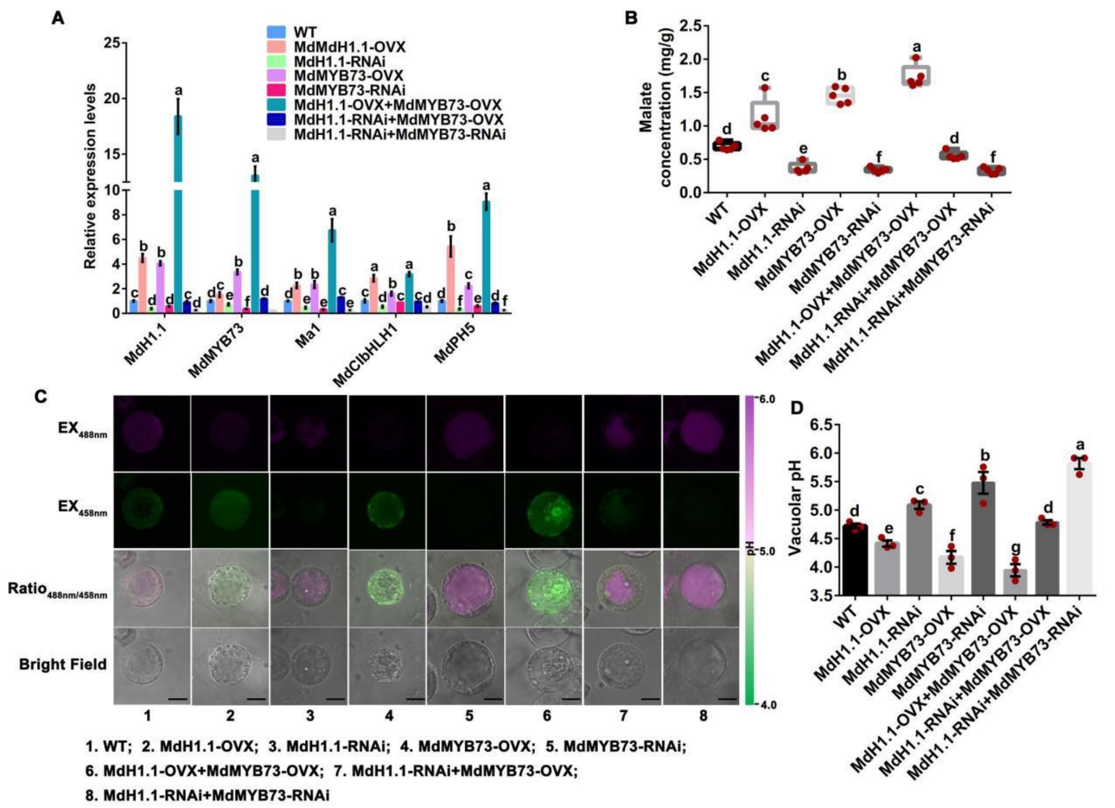

Supplementary Figure S19. MdH1.1 works together with MdMYB73 to modulate malate accumulation and vacuolar acidification.

(A) The relative expression levels of *MdH1.1*, *MdMYB73*, *Ma1*, *MdClbHLH1*, and *MdPH5* genes in the apple calli of wild-type (WT) and *MdH1.1*- and *MdMYB73*-related transgenic lines. OVX represents overexpression; RNAi means suppression; + means co-expression of two vectors in the apple calli. For each sample, the relative expression level of each gene was obtained via RT-qPCR using the ddCT method, with *ACTIN* as an internal reference.

(B) Malate concentrations in WT and transgenic apple calli as indicated in **(A)**. The boxes represent interquartile ranges, with the middle lines as medians and the whiskers as the maximum and minimum values.

(C) Emission intensities of protoplast vacuoles in WT and transgenic calli lines as indicated in **(A)** loaded with BCECF at excitation wavelengths of 488 nm (first column; purple) and 458 nm (second column; green). Ratio images indicate a decreased vacuolar pH in transgenic apple calli relative to the WT calli. The pseudo-color scale on the right indicates intensity of fluorescence, with yellow and purple representing minimum and maximum intensity, respectively. Scale bar = 10 μm.

(D) Quantification of the luminal pH in vacuoles of wild-type and transgenic calli lines as indicated in **(A)**. Data are mean ± SE of 3 replicates with 3 intact vacuoles per

replicate. Different letters indicate significant difference using Tukey’s HSD test at $P < 0.05$ after ANOVA.

In **(A)** and **(B)**, data are mean ± SE of 5 replicates with calli grown in one petri dish as a replicate. Different letters indicate significant difference using Tukey’s HSD test at $P < 0.05$ after ANOVA.

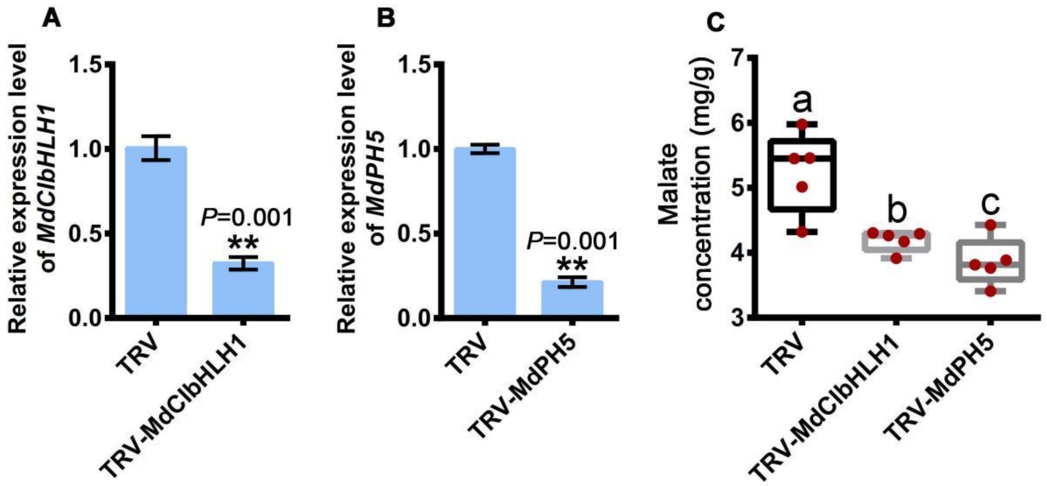

Supplementary Figure S20. MdCIbHLH1 and MdPH5 play a role in the accumulation of malate in apple fruit.

(A) and **(B)** The relative expression levels of *MdCIbHLH1* and *MdPH5* in the cortex tissues of wild-type fruit agro-infiltrated with antisense *MdCIbHLH1* or antisense *MdPH5* viral vectors. The antisense cDNA fragments of *MdH1.1* and *MdMYB73* were inserted into the TRV vector for suppression, respectively, with empty vector as control. For each sample, the relative expression level of each gene was obtained via RT-qPCR using the ddCT method, with *ACTIN* as an internal reference. Data are mean ± SE of 5 biological replicates with 3 fruits per replicate. Statistical significance was determined using Student's *t*-test. $^{**}P < 0.001$.

(C) Malate concentrations of the fruit cortex tissues agro-infiltrated with antisense *MdCIbHLH1* or antisense *MdPH5* viral vectors as indicated in **(A)** and **(B)**. Data are obtained from 5 biological replicates with 3 fruits per replicate. The boxes represent interquartile ranges, with the middle lines as medians and the whiskers as the maximum and minimum values. Different letters indicate significant difference at $P < 0.05$ using Tukey's HSD test after ANOVA.

Supplementary Table S1 List of PCR primers used in this study.

Name	Primer sequences	Note
MdH1.1-F1 (chip)	TCAACTCACAATTCCCGTT	ChIP-PCR primers
MdH1.1-R1 (chip)	GTTATTGACTCGGCGGTTT	
MdH1.1-F2 (chip)	TCCAACCTTAACACCCATC	
MdH1.1-R2 (chip)	GGTGTGTTTCTAAGTGGAT	
MdH1.1-F3 (chip)	GTTCAACTTCATCGTCTTTC	
MdH1.1-R3 (chip)	GTGTTGGATTGTTGTAGGC	
MdH1.1-F4 (chip)	CTAAACCGTGTGTATTGGAC	
MdH1.1-R4 (chip)	GTCCATTCATGTCTCCTTTC	
MdH1.1-F5 (chip)	TTAACCAGCACATATGTATG	
MdH1.1-R5 (chip)	ACCCTTGAATGATGATTGTG	
MdH1.1-F6 (chip)	GTTAGTTTCCATGATTCCA	
MdH1.1-R6 (chip)	ACACATTAGGCCTAAGAAC	
MdMYB73-F1 (chip)	GTCTCAATATATCGGGGGA	
MdMYB73-R1 (chip)	GTACATGCGCACCCCTTGT	
MdMYB73-F2 (chip)	ATCTGTGTGCGAAATACTT	
MdMYB73-R2 (chip)	CTCACAATAGGCTAGCAAT	
MdMYB73-F3 (chip)	CACAATATTATCCGTGTACG	
MdMYB73-R3 (chip)	ACATCTTAAGAGCGCGCGT	
MdPH5-F (chip)	CTCTTCCAACCCAGTTCTT	
MdPH5-R (chip)	CAGCATCATGTCTCAATTC	
MdCIbHLH1-F (chip)	CCTTTCGGTTTCTGCTGAT	
MdCIbHLH1-R (chip)	AAACACCACCGTTCAGCCT	
MdMYB73-T1F(probe-EMSA)	CTCAATATATCGGGGGAAGC CCGAAATGGTAAATGAAC	EMSA primers
MdMYB73-T1R(probe-EMSA)	GTTCATTTACCATTTCGGGC TTCCCCCGATATATTGAG	
MdMYB73-mT1F(probe-EMSA)	CTCAATATATCGGGGGAAAA AAAAAATGGTAAATGAAC	
MdMYB73-mT1R(probe-EMSA)	GTTCATTTACCATTTTTTTTTT CCCCCGATATATTGAG	
MdMYB73-T2F(probe-EMSA)	TTAATCTGATTATTATCTGTG TGCGAAATACTTTTTTT	
MdMYB73-T2R(probe-EMSA)	AAAAAAAGTATTTCGCACAC AGATAATAATCAGATTAA	
MdMYB73-mT2F(probe-EMSA)	TTAATCTGATTATTATCTGTA AAAAAAATACTTTTTTT	

MdMYB73-mT2R(probe-EMSA)	AAAAAAAGTATTTTTTTTACA GATAATAATCAGATTAA
MdMYB73-T3F(probe-EMSA)	TTTATAACCGGCATTACGCG CGCTCTTAAGATGT
MdMYB73-T3R(probe-EMSA)	ACATCTTAAGAGCGCGCGTA ATGCCGGTTATAAA
MdMYB73-mT3F(probe-EMSA)	TTTATAACCGGCATAAAAAAA AAACTTAAGATGT
MdMYB73-mT3R(probe-EMSA)	ACATCTTAAGTTTTTTTTTTAT GCCGGTTATAAA
MdCIbHLH1-F(probe-EMSA)	CTGCTGATTCTGTCTCTTTC CCGAGAAAAACCCAG
MdCIbHLH1-R(probe-EMSA)	CTGGGTTTTTCTCGGGAAAG AGACAGAATCAGCAG
MdCIbHLH1-mF(probe-EMSA)	CTGCTGATTCTGTCTCAAAA AAAAGAAAAACCCAG
MdCIbHLH1-mR(probe-EMSA)	CTGGGTTTTTCTTTTTTTTGA GACAGAATCAGCAG
MdPH5-F(probe-EMSA)	ACACGTTGTGAGACAATGGC GCGACTTGAATTGAG
MdPH5-R(probe-EMSA)	CTCAATTCAAGTCGCGCCAT TGTCTCACAACGTGT
MdPH5-mF(probe-EMSA)	ACACGTTGTGAGACAAAAAA AAAAATTGAATTGAG
MdPH5-mR(probe-EMSA)	CTCAATTCAATTTTTTTTTTT GTCTCACAACGTGT
MdH1.1-1F(probe-EMSA)	TGATTCTGCTTCCGAGTTAA CTGAGTCAAACCGC
MdH1.1-1R(probe-EMSA)	GCGGTTTGACTCAGTTAACT CGGAAGCAGAATCA
MdH1.1-m1F(probe-EMSA)	TGATTCTGCTTCCGAGTAAA AAAAGTCAAACCGC
MdH1.1-m1R(probe-EMSA)	GCGGTTTGACTTTTTTTACTC GGAAGCAGAATCA

MdH1.1-2F(probe-EMSA)	CTAAAATCCATCAACTACAA CCAAAGTTACACCCATT
MdH1.1-2R(probe-EMSA)	AATGGGTGTAACTTTGGTTG TAGTTGATGGATTTTAG
MdH1.1-m2F(probe-EMSA)	CTAAAATCCATCAACTAAAAA AAAAGTTACACCCATT
MdH1.1-m2R(probe-EMSA)	AATGGGTGTAACTTTTTTTTT AGTTGATGGATTTTAG
MdH1.1-3F(probe-EMSA)	CAGAATTTCACGATTCTTTAA CCATTTCACTCCCCTG
MdH1.1-3R(probe-EMSA)	CAGGGGAGTGAAATGGTTAA AGAATCGTGAAATTCTG
MdH1.1-m3F(probe-EMSA)	CAGAATTTCACGATTCTTAAA AAATTTCACTCCCCTG
MdH1.1-m3R(probe-EMSA)	CAGGGGAGTGAAATTTTTTA AGAATCGTGAAATTCTG
MdH1.1-4F(probe-EMSA)	GTACAATGACGAGAATTATA ACCATTCATTCTTTTATC
MdH1.1-4R(probe-EMSA)	GATAAAAGAATGAATGGTTA TAATTCTCGTCATTGTAC
MdH1.1-m4F(probe-EMSA)	GTACAATGACGAGAATTATA AAAAAACATTCTTTTATC
MdH1.1-m4R(probe-EMSA)	GATAAAAGAATGTTTTTTTAT AATTCTCGTCATTGTAC
MdH1.1-5F(probe-EMSA)	AATTTTCATGTAATGAACAGT TGTTTTAGAAGAAAATG
MdH1.1-5R(probe-EMSA)	CATTTTCTTCTAAAACAACTG TTCATTACATGAAAATT
MdH1.1-m5F(probe-EMSA)	AATTTTCATGTAATGAAAAAA AATTTTAGAAGAAAATG
MdH1.1-m5R(probe-EMSA)	CATTTTCTTCTAAAATTTTTTT TCATTACATGAAAATT
MdH1.1-6F(probe-EMSA)	AGGGAGCCAAGCCAACGGC CAAGGACAATTAGATGTG

MdH1.1-6R(probe-EMSA)	CACATCTAATTGTCCTTGGC<br>CGTTGGCTTGGCTCCCT	
MdH1.1-m6F(probe-EMSA)	AGGGAGCCAAGCAAAAAAC<br>CAAGGACAATTAGATGTG	
MdH1.1-m6R(probe-EMSA)	CACATCTAATTGTCCTTGGTT<br>TTTTGCTTGGCTCCCT	
pMdMYB73-1620F(+A)	ACAATATTATCCGTGTACGTA	
pMdMYB73-1620R(+A)	GGAAGATAGAGAGGAGAGGAG	
pMdMYB73-1427F(+A)	AACCCGTTGTGAGGCTAAGCC	
pMdMYB73-1427R(+A)	GGAAGATAGAGAGGAGAGGAG	
pMdMYB73-1000F(+A)	ACACAAAATCATCATTCACGTT	
pMdMYB73-1000R(+A)	GGAAGATAGAGAGGAGAGGAG	
pMdMYB73-1000TF(+A)	ACACAAAATCATCATTCACGTT	GUS vector primers
pMdMYB73-1000TR(+A)	TTCCCCCGATATATTGAGAC	
pMdMYB73-600F(+A)	ACCTTTCCCAAAACATGACAGC	
pMdMYB73-600R(+A)	GGAAGATAGAGAGGAGAGGAG	
pMdCIbHLH1-1000F(+A)	AAGGAAAGTACGTTAATAGCC	
pMdCIbHLH1-1000R(+A)	CCAAACACCACCGTTCAGCC	
pMdCIbHLH1-1000TF(+A)	AAGGAAAGTACGTTAATAGCC	
pMdCIbHLH1-1000TR(+A)	AGGGAGGAAGAAATGGAGGT	
pMdCIbHLH1-100F(+A)	AACCTCCATTTCTTCCTCCCT	
pMdCIbHLH1-100R(+A)	CCAAACACCACCGTTCAGCC	
pMdPH5-1000F(+A)	AAGCTTTGAAAGGTAGTTAAAC	
pMdPH5-1000R(+A)	AATCAAGTAGGTACAGAATCG	
pMdPH5-700F(+A)	AAGAGGAGGTGTCAAAGGAAAT	
pMdPH5-700R(+A)	AATCAAGTAGGTACAGAATC	

	G	
MdH1.1-F(62SK-EcoRI)	GAATTCTGACTGGAACTGTCGTCTCC	
MdH1.1-R(62SK-SalI)	GTCGACTTCGCCTTCCTCGCCGGA	
MdMYB73-F(62SK-EcoRI)	GAATTCTGGAAGCGATGAATATGTG	Luciferase (Luc) vector primers
MdMYB73-R(62SK-XhoII)	GTCGAGATTTAATCTATGAAGCTCCG	
pMdMYB73-600F(luc-SalI)	GTCGACCCTTTCCCAAAACATGACAGC	
pMdMYB73-600R(luc-BamHI)	GGATCCGGAAGATAGAGAGGAGAGGAG	
pMdMYB73-1620F(luc-SalI)	GTCGACCAATATTATCCGTGTACGTA	
pMdMYB73-1620R(luc-BamHI)	GGATCCGGAAGATAGAGAGGAGAGGAG	
pMdMYB73-1427F(luc-SalI)	GTCGACACCCGTTGTGAGGCTAAGCC	
pMdMYB73-1427R(luc-BamHI)	GGATCCGGAAGATAGAGAGGAGAGGAG	
pMdCIbHLH1-1000F(luc-SalI)	GTCGACAGGAAAGTACGTTAATAGCC	
pMdCIbHLH1-1000R(luc-BamHI)	GGATCCCCAAACACCACCGTTCAGCC	
pMdPH5-1000F(luc-SalI)	GTCGACAGCTTTGAAAGGTAGTTAAAC	
pMdPH5-1000R(luc-BamHI)	GGATCCAATCAAGTAGGTACAGAATCG	
18S-F	TGACCGAATGAGCAAGGAAATTACT	
18S-R	TACTCAGCTTTGGCAATCCACATC	
MdH1.1-F (qPCR)	TGCTCCGGCGAAAGAGAAG	

MdH1.1-R (qPCR)	TTGGGCTTAGAAGCTACCGC	
MdMYB73-F(qPCR)	AGTAGAGAGCAGATATCCGAC	
MdMYB73-R(qPCR)	ATCATGATCATGCACTGCTAC	
MdPH1-F(qPCR)	AAGAATCCCACACAAGAAGC	qPCR primers
MdPH1-R(qPCR)	AAATGTTTGAAGATTGGATC	
MdPH5-F(qPCR)	ATAGCGTCTCCTTCCTTACT	
MdPH5-R(qPCR)	AGTGAGCACTTGGGGAAGA	
MdCIbHLH1-F(qPCR)	AGGACGAGATGGGTTCTTC	
MdCIbHLH1-R(qPCR)	ATCTGAATGGCCCTGGATG	
Ma1-F(qPCR)	CAGGATTTTGTGCTAGTTACGCC	
Ma1-R(qPCR)	CAGCAATAAGCAGCAATCGATA	
MdWRKY53-F (qPCR)	AACTATTACTTTGAGCTGCC	
MdWRKY53-R (qPCR)	ACGAATTGTTGGCTGAGATG	
MdWRKY53L-F (qPCR)	ATCGTCTCAGCCAAAAATTC	
MdWRKY53L-R (qPCR)	AGTCAAACGGGAAATAGGAG	
MdCIbHLH1-F(TRV-EcoRI)	GAATTCACCTCCATTTCTTCCTCCCT (163bp)	
MdCIbHLH1-R(TRV-SacI)	GAGCTCCCAAACACCACCGTTCAGCC	
MdPH5-F(TRV-EcoRI)	GAATTCAGAGGAGGTGTCAAAGGAAAT (177bp)	
MdPH5-R(TRV-SacI)	GAGCTCAGTACCCGAGTGAGCACTTG	
MdH1.1-RNAiF(TRV-EcoRI)	GAATTCAGAGTCTTTAAGAGACTGCC	VIGS primers
MdH1.1-RNAiR(TRV-BamHI)	GGATCCAAACCGATTTGTTTTTTTCAT	
MdWRKY53-F (TRV-EcoRI)	GAATTCACGAATTGTTGGCTGAGATG	

MdWRKY53-R (TRV-BamHI)	GGATCCCAGCATCTCCATTCTCTTT	
MdWRKY53L-F (TRV-EcoRI)	GAATTCAGTCAAACGGGAAATAGGAG	
MdWRKY53L-R (TRV-BamHI)	GGATCCATCAATCATTGCTATCTCCG	
MdH1.1-F(+A-62)	AGTTAGTTTCCATGATTCCA	Full length ORF of MdH1.1
MdH1.1-R(+A-62)	GTTATTGACTCGGCGGTTT	
MdH1.1-RNAiF(EcoRI)	GAATTCAGAGTCTTTAAGAGACTGCC	RNAi of MdH1.1
MdH1.1-RNAiR(BamHI)	GGATCCAAACCGATTTGTTTTTTTCAT	
MdH1.1-RNAi-F	GGGGACAAGTTTGTACAAAAAAGCAGGCT<u>CCTGCTGCCAAGGCG</u>	Gateway primer for MdH1.1 RNAi
MdH1.1-RNAi-R	GGGGACCACTTTGTACAAGAAAGCTGGGT<u>TCACTTCTTCGCCTTCCTCG</u>	
