# Supplementary material for: A linker histone acts as a transcription factor to orchestrate malic acid accumulation in apple in response to sorbitol
Source: Plant Cell. 2024 Dec 20;37(1):koae328. doi: 10.1093/plcell/koae328 (PMC11773815; doi:10.1093/plcell/koae328)
Supplement: koae328_Supplementary_Data [file tpc.00586.2024-s11.pdf › tpc.00586.2024-s11.pdf]

THE PLANT CELL

A Linker Histone Acts as a Transcription Factor to Orchestrate Malic Acid Accumulation in Apple in Response to Sorbitol

Da-Gang Hu, Mengxia Zhang, Chunlong Li, Tingting Zhao, Lian-Da Du, Quan Sun, Chu-Kun Wang, Dong Meng, Cui-hui Sun, Zhangjun Fei, Abhaya Dandekar, and Lailiang Cheng

Review Timeline:

Submission Date:	18-Jun-2024
Editorial Decision:	22-Aug-2024
Revision Received:	01-Oct-2024
Editorial Decision:	02-Nov-2024
Revision Received:	13-Nov-2024
Accepted:	15-Nov-2024

Prof. Lailiang Cheng
Cornell University
School of Integrative Plant Science, Horticulture Section
Ithaca, New York 14853

Dear Dr. Cheng:

We have received reviews of your manuscript entitled "A Linker Histone Acts as a Transcription Factor to Orchestrate Malic Acid Accumulation in Apple in Response to Sorbitol." Thank you for submitting your best work to The Plant Cell. The editorial board agrees that the work you describe is substantive, falls within the scope of the journal, and may become acceptable for publication, pending revision and potential re-review.

We think both referees have provided constructive reviews aimed at strengthening the analyses and improving the presentation of the manuscript. Both reviewers appreciate the novel insights into the regulation of malic acid accumulation. However, Reviewer 2 raises major concerns about one of the main conclusions of the paper, that MdH1.1 acts as a transcription factor with sequence-specific binding. We particularly ask you to address their comments as thoroughly as possible with appropriate revisions including additional experiments to support your claims. However we should stress that we are reluctant to see manuscripts undergoing multiple rounds of revision and would be unlikely to offer you more than one chance to satisfy the reviewers.

A note from the Editor-in-Chief: We are trying to make a concerted effort to change green/red comparisons to green/magenta to make our figures understandable to those with color vision deficiencies. We noticed that a small number of your figures utilize red/green contrasts, so as you prepare the next version of the figures, please check the figures for red/green color use. Magenta is a good substitute for red, and a light blue substitutes for green. I mainly noticed this in your bar and line plots - you might consider different also softer (less bold) colors. You might want to check over the Supplemental Figures for similar comparisons. Please note that color changes do not need to be highlighted or tracked in the revised manuscript, but could be noted in the cover letter or response document.

Please contact us if there are ambiguous comments or if you wish to discuss the revision.

Given the nature of the comments, we are offering you 60 days from when we have issued this decision to complete the revision. If a revision is not returned within this time frame, and if you have not been granted an extension, we will withdraw the manuscript, which will leave you free to submit the work elsewhere. If you need an extension, we encourage you to contact us at any point before the 60 days have passed.

When you are ready to submit the revised version, please upload a highlighted copy that indicates all changes made in response to the editor and reviewer recommendations. Include an itemized list of all changes made in response to each of the reviewer's suggestions in the "Response to Reviewers" section; please note that reviewers do not have access to your cover letter, nor was this decision letter shared with them.

Thank you for the privilege of reviewing your work. We look forward to receiving your revised manuscript.

On behalf of the editorial board,

Reviewing Editor, Board of Reviewing Editors
Robert Schmitz, Senior Editor
Blake Meyers, Editor-in-Chief

The Plant Cell

Please note the following:

-The Plant Cell now requires authors to complete and submit an author revisions checklist upon submission of a revised manuscript. The aim of the checklist is to aid authors in preparing a high-quality manuscript, facilitate the review and assessment of revised manuscripts, and help to ensure that journal standards are maintained across the board. If your manuscript is accepted, the completed checklist will be published as supplemental material attached to the article online. Please download a copy of the checklist (pdf fillable form) at this link, for submission with your revised manuscript: https://tpc.msubmit.net/html/Author_Revisions_Checklist.pdf.

-Supplemental materials should be restricted to large datasets and tables, presentation of replicates, and validation of reagents, methods, or genotypes. Any data that are used to support the major claims must be in the main manuscript.

Supplemental figure legends must indicate what figure in the main manuscript is supported by the supplemental data presented. Please justify how each of the supplemental figures meet the criteria.

-Sampling methods and nature of "biological replicates" should be described precisely (i.e. different plants, parts of plants, pooled tissue, independent pools of tissue, sampled at different times, etc), along with a clear description of and rationale for any statistical analyses conducted. The reader should know exactly what was sampled; what forms the basis of the calculation of any means and statistical parameters reported. This is also necessary to ensure that proper statistical analysis was conducted.

-Want to add this revision deadline to your calendar? Click below!

--- Reviewer comments:

Reviewer #1 (Comments for the Author):

Malate is the major organic acid accumulated in fleshy fruits including apple, and vacuolar accumulation of malate determines fruit acidity and perception of sweetness. An earlier study has shown that in apple malate accumulation is dependent on sorbitol. Antisense suppression of aldose-6-phosphate reductase (A6PR), the key enzyme in sorbitol biosynthesis reduces sorbitol and malate accumulation as well as expression of genes involved in vacuolar transport of malate. Although transcription factors and key enzymes involved in vacuolar malate transport is well documented, the molecular mechanism underlying sorbitol-dependent malate accumulation in apple is not known. In this study, the authors identified a linker histone H1 variant, MdH1.1 that functions as a transcription factor and forms a positive feedback loop with a previously characterized MYB factor, MYB73, to regulate sorbitol-dependent malate accumulation in apple. The authors provided adequate evidence to support the claims made. This manuscript provides novel and interesting information on regulation of sorbitol-induced malate accumulation in apple. However, manuscript requires revision.

There are a number of mistakes/typos throughout the manuscript. Additionally, I have some queries about some of the experiments. The authors should address these issues in the manuscript.

line 182/185/186 Cite the figure as Figure 2 (A-G is not required, and is not there in the Figure).

line 225/226 Figure 3 A-F numbering missing in the figure and is not required. correct it in the text.

line 235 Supplemental Figure 2: This figure is not related to sorbitol treatment of leaf. It shows the sorbitol and malate concentration in fruits.

line 330/333/344/346 check and correct the Figure numbers in the Figure match with the text

line 388 it should be Supp. Figure 11B to 11H.

line 392 MdMYB73, MdClbHLH1, and MdPH5 promoters

line 399 should be Supp. Figures 12B to 12E

line 406-407 should be Supp. Figures 12F and 12G

line 410 is it Royal Gala transgenic plants or callus?

line 422 activation domain pGADT7

line 454 CANNTG-containing oligonucleotide? check and correct.

line 455 should be (Figures 10D to 10F)

line 514 should be (Supplemental Figures 14C and 14D).

line 518 should be Supplemental Figures 15

line 588 is the figure number correct?

Figures:

Figure 6B: correct the legend to "50mM glucose".
correct it to match the text. reorganize the Figure 7L and 7M. correct the legend. Include a legend for corrected Figure 7M.
Figure 7J: should be 7I.
Figure 7K: should be 7J.
Figure 7L: should be 7K
Figure 7M: should be 7L
Figure 7I: should be 7M
Figure 8A: which one is "the typical binding" motif core sequence?
Figure 8A legend: The grey rectangles represent the WHD-binding motif. What does the "green rectangle" represent?
Figure 8F: what is the difference between MYB73-T1 and MYB73-T3 (other than the fragment is longer?)
Figure 9B-C: it seems to me the binding motifs have no additive effects on activation? why did the authors use three different fragments for LUC assay. A single assay is sufficient? correct me if I am wrong?
Supp. Fig. 11: why did the authors use a longer and shorter version of the promoter with the binding motif?
Supp. Fig. 13: MdH1.1 and MdClbHLH1 both cloned in GADT7 (-Leu selection). How do the authors select for both plasmids in yeast, can be either one, the cells still can grow on selection medium? Is this data quantitative?
Figure 10A: I am really surprised to see a nominal (~10-20%) increase (H1.1 expression in MYB73-OE, line #12, 14) is statistically significant.
Yeast one hybrid assay: A number of Y1H assays were performed here using different promoter/promoter fragments and transcription factors. It is really surprising to see that 200mM 3AT was invariably used in all assays to eliminate the background activity (threshold is same for all promoters!).

Reviewer #2 (Comments for the Author):

This is a comprehensive study conducted in apple to dissect the regulation of malate levels through sorbitol.

Starting out from the observation that antisense expression of ALDOSE-6-PHOSPHATE REDUCTASE in apple decreases sorbitol and malate levels, the authors set out to identify misregulated genes in these antisense lines and identified a set of candidate genes involved in malate transport and vacuolar acidification.
They then tested, which genes would respond positively and specifically to sorbitol, and identified a set of potential transcription factors, one of them being MdH1.1, a homologue of the Arabidopsis H1 protein. Promoter-GUS fusion constructs confirm the sorbitol-induced expression. The authors then confirm that MdH1.1 complements the Arabidopsis 3h1 mutant and would therefore be a linker H1 histone.
They then find that MdH1.1 and MdMYB73 are respectively required for upregulation of the each other upon sorbitol. They further find that MdH1.1 binds to the MYB73 promoter in Y1H and gel shift assays and mutating the WHD-binding cis elements abolishes binding. Therefore MdH1.1 binds to the MYB73 promoter to activate its gene acting synergistically with another factor namely MdClbHLH1. Reversely, they find that MdMYB73 controls the MdH1.1 gene establishing a positive feedback loop.

This is a thorough study with many independent experimental approaches to prove a specific point. The authors identify key factors involved in malate accumulation in response to sorbitol in apple. I have, however, questions concerning one of the main conclusions of the paper that MdH1.1 acts as a transcription factor showing sequence-specific binding. In the literature, H1 is described to bind to the nucleosome dyad via its GH1 domain and to interact with linker DNA, which suggest that it binds DNA in a non-sequence specific way. How do the authors reconcile their observations with the data from the literature that H1 binds generally to nucleosomes and that it is associated to nucleosome compaction and in Arabidopsis also favors H3K27me3 enrichment at genes (Teano et al., Cell reports, 2023) so would be rather operating by gene repression?

Can the authors show in Figure 8B that in a nucleosomal context, MdH1.1 binds specifically to the MYB73 promoter and not (or less) elsewhere - by including additional negative controls such as other promoters without WDH binding sites or a heterochromatic target? For the EMSA assays, can the authors include another longer promoter region from an unrelated gene without WHD binding elements?

Can an alternative scenario be envisaged where MdGH1.1 would rather stabilize binding of another transcription factor such as MdClbHLH1 that activates the target genes? A similar scenario has been proposed in the publication Soler, New Phytologist, 2017, https://doi.org/10.1111/nph.14129, in which the authors show that EgH1.3 interacts with EgMYB1 to enhance transcriptional repression of EgMYB1 target genes. Here it would not be EgH1.1 that brings the specificity and its role is rather transcriptional repression.

In the Y1H assays, MdH1.1 is fused to the activation domain, and it is shown to activate transcription of the reporter when under control of the MYB73 promoter. This suggests that it activates transcription through sequence specific

binding. At the same time, in Figure 7K the authors observe auto-activation in the Y2H system, when MdH1.1 is fused to the DNA binding domain suggesting that it would (also?) comprise an activation domain. Again, this feature has to my knowledge never been observed for H1 homologs in other species. Is H1.1 among the genes predicted to contain an activation domain (https://doi.org/10.1016/j.cels.2024.05.007)?

Minor points:

- Figure 7: It is curious that MdH1.2, given the proximity in the evolutionary tree, does not complement the 3h1 mutant. Could the authors include in Figure S5 the alignment with the MdH1.2? Are any critical amino acids missing in this protein that would explain why it would not complement the 3h1 phenotype? If most of the critical amino acids are conserved in MdH1.2 in WHD/globular domain, should it not act similar to MdH1.1 also in transcriptional activation?

- Figure 7H: It is difficult to appreciate the complementation in these images, which look not really like Arabidopsis nuclei. Loss of H1 in Arabidopsis causes decondensation of most chromocenters except those that comprise the rDNA (see Figure 2B, Rutowiscz, et al, 2019). In the Figure 7H, either no or only 2 chromocenter structures are visible, and the chromatin structure looks quite bizarre.

- Figure 2 --- text refers to several panels A-G, no panel is indicated in the figure.
- page 14 line 388 it should be 11B to H.
- page 15 line 407 it should be 12F and G.

In general, the manuscript is a bit lengthy. To ease reading, the authors may want to consider whether certain details could be removed or some Figures with similar experiments could be combined.

Responses to Review

We thank both reviewers for the constructive comments/suggestions on an earlier version of our manuscript (TPC2024-RA-00586). To address the review comments, we have conducted additional experiments and revised the manuscript. The following are our responses to specific review comments (*Review comments in italics*).

Reviewer 1

line 182/185/186 Cite the figure as Figure 2 (A-G is not required, and is not there in the Figure).
line 225/226 Figure 3 A-F numbering missing in the figure and is not required. correct it in the text.
line 235 Supplemental Figure 2: This figure is not related to sorbitol treatment of leaf. It shows the sorbitol and malate concentration in fruits.
line 330/333/344/346 check and correct the Figure numbers in the Figure match with the text
line 388 it should be Supp. Figure 11B to 11H.
line 392 MdMYB73, MdCIbHLH1, and MdPH5 promoters
line 399 should be Supp. Figures 12B to 12E
line 406-407 should be Supp. Figures 12F and 12G

Response: We checked and corrected all the mismatches.

line 410 is it Royal Gala transgenic plants or callus?

Response: It's Royal Gala transgenic plants. We specified it.

line 422 activation domain pGADT7
line 454 CANNTG-containing oligonucleotide? check and correct.
line 455 should be (Figures 10D to 10F)
line 514 should be (Supplemental Figures 14C and 14D).
line 518 should be Supplemental Figures 15
line 588 is the figure number correct?

Response: All checked and corrected.

Figures:
Figure 6B: correct the legend to "50mM glucose".
correct it to match the text. reorganize the Figure 7L and 7M. correct the legend. Include a legend for corrected Figure 7M.
Figure 7J: should be 7I.
Figure 7K: should be 7J.
Figure 7L: should be 7K
Figure 7M: should be 7L
Figure 7I: should be 7M

Response: Checked and corrected.

Figure 8A: which one is "the typical binding" motif core sequence?
Figure 8A legend: The grey rectangles represent the WHD-binding motif. What does the "green rectangle" represent?

Response: Both are core sequence motifs. We could not tell which one is more typical. We removed “typical” in any of the description. We modified the Fig 8A legend to clarify the grey box.

Figure 8F: what is the difference between MYB73-T1 and MYB73-T3 (other than the fragment is longer?)

We found an error in the earlier version of the diagram showing the various fragments of the *MYB73* promoter. We re-drew the diagram and re-did the Y1H assays, focusing on testing the 3 putative WHD-binding cis-elements separately in addition to all three together, with the *MYB1* promoter without a WHD-binding cis-element as an additional negative control. The new diagram and the Y1H data are presented in the revised Fig 8F and 8G.

Figure 9B-C: it seems to me the binding motifs have no additive effects on activation? why did the authors use three different fragments for LUC assay. A single assay is sufficient? correct me if I am wrong?

We found an error in labeling the Luc imaging assays of the various fragments of the *MYB73* promoter in the earlier version. The correct labeling is presented in the revised Fig 9B-C. We focused on testing the activity of the 3 WHD-binding cis-elements individually using the Luc assay, and the data show that binding of MdH1.1 to each enhances their activity.

Supp. Fig. 11: why did the authors use a longer and shorter version of the promoter with the binding motif?

Response: Having T3, the longer version with the binding motif is not essential, but it allowed us to see if the length of the non-motif containing region makes any difference.

Supp. Fig. 13: MdH1.1 and MdCIbHLH1 both cloned in GADT7 (-Leu selection). How do the authors select for both plasmids in yeast, can be either one, the cells still can grow on selection medium? Is this data quantitative?

Response: We used PCR to detect the presence of both *MdH1.1* and *MdCIbHLH1* in the yeast clone used for the Y1H assay. Y1Hs are largely qualitative, but additive effects of MdH1.1 and MdCIbHLH1 on yeast growth were clearly seen.

Figure 10A: I am really surprised to see a nominal (~10-20%) increase (H1.1 expression in MYB73-OE, line #12, 14) is statistically significant.

Response: Two factors have contributed to the statistical difference here: 1) the actual increases in *H1.1* expression levels are 49% and 88% in MYB73-OE lines 12 and 14, respectively; 2) the small SEs helped as well.

Yeast one hybrid assay: A number of Y1H assays were performed here using different promoter/promoter fragments and transcription factors. It is really surprising to see that 200mM 3AT was invariably used in all assays to eliminate the background activity (threshold is same for all promoters!).

Response: We tested a range of 3-AT concentrations for each promoter and transcription factor combination (0, 50, 100 and 200 mM). We found that 50 mM was not effective in eliminating background activity, 100 mM was effective for some, but not for others whereas 200 mM was effective in eliminating the background activity for all combinations. We decided to use 200 mM 3-AT in all combinations, which allowed us to look at any additive effect of having two transcription factors in GADT7 on yeast growth.

Reviewer 2

This is a thorough study with many independent experimental approaches to prove a specific point. The authors identify key factors involved in malate accumulation in response to sorbitol in apple. I have, however, questions concerning one of the main conclusions of the paper that MdH1.1 acts as a transcription factor showing sequence-specific binding. In the literature, H1 is described to bind to the nucleosome dyad via its GH1 domain and to interact with linker DNA, which suggest that it binds DNA in a non-sequence specific way. How do the authors reconcile their observations with the data from the literature that H1 binds generally to nucleosomes and that it is associated to nucleosome compaction and in Arabidopsis also favors H3K27me3 enrichment at genes (Teano et al., Cell reports, 2023) so would be rather operating by gene repression?

Response: we do not have a complete answer to the reconciliation question at this point, but our data strongly suggest that MdH1.1 has the capacity to do both and these two types of binding are not mutually exclusive to each other. As discussed briefly in the revised text, how this is accomplished by MdH1.1 warrants further research. As all our data point to MdH1.1 being a transcriptional activator for *MYB73* in addition to being an architectural protein for chromatin, it is unlikely that our observations are the result of MdH1.1 operating by gene repression via general binding to nucleosomes and associated nucleosome compaction, especially considering that the indirect effects of MdH1.1 via its physical interaction with two other proteins MdCIbHLH1 and MdBT2 are excluded experimentally (See our response to your alternative scenario question below).

Can the authors show in Figure 8B that in a nucleosomal context, MdH1.1 binds specifically to the MYB73 promoter and not (or less) elsewhere - by including additional negative controls such as other promoters without WDH binding sites or a heterochromatic target? For the EMSA assays, can the authors include another longer promoter region from an unrelated gene without WHD binding elements?

Response: We included the promoter of an unrelated gene without a WHD-binding site, *MYB1*, as an additional control in our ChIP-PCR (Fig. 8B). For the EMSA assay, we included a longer fragment of the *MYB73* promoter without a WHD-binding cis-element and a fragment of another gene *tDT* with a putative WHD-binding cis-element but MdH1.1 does not bind to (Supplemental Fig S10). In addition, we re-did the Y1H assay, with the promoter of *MYB1* as an additional control (Fig. 8G). These new data along with the earlier data clearly show that MdH1.1 specifically binds to the WHD cis-elements in the promoter of *MYB73* as a transcription factor.

Can an alternative scenario be envisaged where MdGH1.1 would rather stabilize binding of another transcription factor such as MdClbHLH1 that activates the target genes? A similar scenario has been proposed in the publication Soler, New Phytologist, 2017,, in which the authors show that EgH1.3 interacts with EgMYB1 to enhance transcriptional repression of EgMYB1 target genes. Here it would not be EgH1.1 that brings the specificity and its role is rather transcriptional repression.

Response: We examined two alternative scenarios where MdH1.1 could indirectly alter the expression of its downstream target genes: 1) Interaction with MdCIbHLH1 on the transactivation activity of MdMYB73; and 2) Interaction with the BTB-TAZ domain protein MdBT2 on the transactivation activities of MdCIbHLH1 and MdMYB73. Both Y2H and BiFC assays show that MdH1.1 does not physically interact with either MdCIbHLH1 or MdBT2 (Supplemental Fig. S17). Exclusion of these two indirect pathways for the action of MdH1.1 further strengthens the conclusion that MdH1.1 directly activates the transcription of MdMYB73 by specifically binding to the WHD cis-elements in its promoter.

In the Y1H assays, MdH1.1 is fused to the activation domain, and it is shown to activate transcription of the reporter when under control of the MYB73 promoter. This suggests that it activates transcription through sequence specific binding. At the same time, in Figure 7K the authors observe auto-activation in the Y2H system, when MdH1.1 is fused to the DNA binding domain suggesting that it would (also?) comprise an activation domain. Again, this feature has to my knowledge never been observed for H1 homologs in other species. Is H1.1 among the genes predicted to contain an activation domain (https://doi.org/10.1016/j.cels.2024.05.007)?

Response: We went through the list of genes identified by Hummel et al (2024) (https://doi.org/10.1016/j.cels.2024.05.007) and found that two genes (At1G54230 and At1G54240) with the H1 globular domain at the bottom of the phylogenetic tree (Supplemental Fig S4) are among the genes predicted to have an activation domain, which corresponds to the H1 globular domain. However, H1.1 is not on the list. The most likely explanation for this discrepancy is that the algorithm used by the authors may be too restrictive as all 15 Arabidopsis genes in the phylogenetic tree including the three canonical H1s have the H1 globular domain. In our work, we experimentally showed that MdH1.1 has autoactivation activity in yeast just like another transcription factor MYB73 (Fig. 7K), and that MdH1.1 binds to the WHD cis-elements in the promoter of *MYB73*, activating its transcription (Figures 8 and 9; Supplemental Figs S10).

Minor points:

- Figure 7: It is curious that MdH1.2, given the proximity in the evolutionary tree, does not

complement the 3h1 mutant. Could the authors include in Figure S5 the alignment with the MdH1.2? Are any critical amino acids missing in this protein that would explain why it would not complement the 3h1 phenotype? If most of the critical amino acids are conserved in MdH1.2 in WHD/globular domain, should it not act similar to MdH1.1 also in transcriptional activation?

Response: We included MdH1.2 in the alignment figure (Supplemental Fig S5) and conducted promoter binding assays for MdH1.2 (Supplemental Fig. S11). We found that MdH1.2 differs with MdH1.1 in only 7 amino acids in the predicted H1 globular domain and 5 amino acids in the predicted WHD binding domain. Both ChIP-PCR and Y1H assays show that MdH1.2 does not bind to the promoter of *MYB73*, and MdH1.2 does not activate the transcription of *MYB73* (Supplemental Fig. S14). So, MdH1.2 is not a transcription factor for *MYB73*, nor is it a functional linker histone. Exploring which amino acids are responsible for the functional difference between MdH1.2 and MdH1.1 in the future would help us better understand the structure-function relationship of MdH1.1 as both a linker histone and a transcription factor.

- Figure 7H: It is difficult to appreciate the complementation in these images, which look not really like Arabidopsis nuclei. Loss of H1 in Arabidopsis causes decondensation of most chromocenters except those that comprise the rDNA (see Figure 2B, Rutowiscz, et al, 2019). In the Figure 7H, either no or only 2 chromocenter structures are visible, and the chromatin structure looks quite bizarre.

Response: We re-did the chromatin analysis to obtain better images showing the difference in chromocenters between Arabidopsis 3h1 and the complemented lines. We used these new images in the revised Fig 7H.

- Figure 2 --- text refers to several panels A-G, no panel is indicated in the figure
- page 14 line 388 it should be 11B to H.
- page 15 line 407 it should be 12F and G.

Responses: All corrected.

In general, the manuscript is a bit lengthy. To ease reading, the authors may want to consider whether certain details could be removed or some Figures with similar experiments could be combined.

Responses: We shortened some sections of the manuscript. However, due to inclusion of significant amount of additional data and corresponding description in the main text, the total length of the manuscript remained largely unchanged. We carefully considered the suggestion of removing some details or combining some figures, but came to the conclusion that the current layout of the figures appears to be the best choice for presenting our data. The length of this paper is comparable with other papers published in TPC.

Dear Dr. Lailiang Cheng:

We have received reviews of your manuscript entitled "A Linker Histone Acts as a Transcription Factor to Orchestrate Malic Acid Accumulation in Apple in Response to Sorbitol." On the basis of the advice received, the board of reviewing editors would like to accept your manuscript for publication in The Plant Cell. This acceptance is contingent on revision based on the comments of our reviewers. In particular, please consider the following:

Please include all text changes suggested by reviewer 1, and especially adapt the statement about TF coexpression to state that the correlation matrix suggests co-expression of MdH1.1 and MdMYB73, and co-expression of MdWRKY53 and MdWRKY53L, but not between all four factors.

Indicate gene names for known GH1-domain proteins in the phylogeny of Fig. S3 and change caption to "Phylogenetic analysis of apple and Arabidopsis GH1-domain proteins"

Thoroughly address the reviewer 2 comment on background activity of the high MdMYB73pro::Luc reporter in S14 (compared to S11 and Fig. 11) and whether transactivation activity of MdH1.2 in this scenario can be still detected. Address this in the text after the statement in L416. Alternatively, you may provide new data to directly compare transactivation activity of MdH1.1 and MdH1.2 on the MdMYB73pro::Luc reporter in the same experiment.

Notes from the Editor-in-Chief
• The color palette in many figures uses very similar tones (for example, blue, purple, and magenta in Figures 1, 3, 4, and 5, or pink/purple in others), which makes rapid visualization of the results challenging. I encourage you to adjust the palette to one with stronger tonal contrasts. Please also avoid, as much as possible, using green/red combinations to accommodate color-blind readers.
• The number of figures in the paper is quite large. While this isn't an issue in itself, extensive papers can discourage readers and may affect the visibility of your work. If possible, consider moving some data (such as control results) to supplementary materials or combining figures where feasible (for example, Figures 1 and 2; 3 and 4; 5 and 6; and 11, 12, and 13 can be merged while maintaining compliance with our guidelines).

Please highlight all changes and include a detailed annotation of changes of the text, with line numbers, and noting your responses to the comments.

To submit your revised manuscript, click:

Link Not Available

If you have any questions about the revision submission procedures, please contact the Editorial Office Staff (tpc-submissions@aspb.org). If you cannot return the revised manuscript within 30 days, please let us know. Otherwise, we will assume that you have elected not to revise the manuscript and withdraw it.

Thank you very much for the privilege of reviewing this work. I look forward to receiving the next version.

On behalf of the editorial board,

Sebastian Soyk, Board of Reviewing Editors
Robert Schmitz, Senior Editor
Pablo Manavella, Incoming Editor-in-Chief

The Plant Cell

Reviewer #1 (Comments for the Author):

The authors have addressed my queries/comments and the revised manuscript is much improved. There are some minor issues and the authors should address these issues in the final version.

line 253-254 "MdH1.1, MdWRKY53, and MdWRKY53L were highly co-expressed with MdMYB73 during fruit development in A4 and WT fruits (Figure 4B)": Is this correct? I think, MdH1.1 highly co-expressed only with MYB73.

line 369 electrophoretic mobility shift assays (EMSAs) by using a prokaryote-expressed....

Supplement Figures 1 and 2 legend: MdALMT9/Ma is annotated as a homolog (not analog) of Arabidopsis ALMT6 here.

Reviewer #2 (Comments for the Author):

The authors have responded to all the questions raised, performed additional experiments, have improved figure quality (Figure 7) and included additional discussion points.

I have only a few minor suggestions:

Figure S3: Phylogenetic analysis of apple and Arabidopsis linker histone proteins.
Not all of these proteins are linker histones. They have been called GH1-domain proteins (PMID: 28298478). Some of them have already names e.g. GH1-HMGA1 or TRB that could be indicated.

Figure S14: Why is the basal luciferase activity so much lower in the S15 (~ 100) and Figure 11, compared to S14? Expression of MdH1.1 stimulates expression to over 500, but those levels are nearly already attained by the MdMYB73 vector alone in S14 questioning whether they could still be stimulated more in this experimental setting?

Reviewing editor comments:

Show individual data points for bar and line plots where the number of data points is less than 6 (e.g. Figs. 1B, 3, 5, 6B, 6D, 6F, 7L, 9C, 9E, 9F, 9G, 10A, 12A, 13, S6, S8B, S8D, S8F, S9).

Show exact P-values for significance statement (e.g. in Figs. 4B, 8B, 9E, 10B, 11F)

Remove statement "One asterisk means significant, and two asterisks mean extremely significant" from the legend of Fig. 4 and the asterisks from panel Fig. 4B.

Change, if appropriate, "Tukey's Honest Significant Difference test" to "Tukey's Honestly Significant Difference test".

Specify in Fig. 7B whether gDNA or cDNA was used as template in the PCR reaction, and indicate the molecular size of the band in the gel.

Remove boxes around Figs. panels 8A, 8B, 10B.

Please also note the following:

-The Plant Cell now requires authors to complete and submit an author revisions checklist upon submission of a revised manuscript. The aim of the checklist is to aid authors in preparing a high-quality manuscript, facilitate the review and assessment of revised manuscripts, and help to ensure that journal standards are maintained across the board. If your manuscript is accepted, the completed checklist will be published as supplemental material attached to the article online. Please download a copy of the checklist (pdf fillable form) at this link, for submission with your revised manuscript: https://tpc.msubmit.net/html/Author_Revisions_Checklist.pdf.

-Supplemental materials should be restricted to large datasets and tables, presentation of replicates, and validation of reagents, methods, or genotypes. Any data that are used to support the major claims must be in the main manuscript. Supplemental figure legends must indicate what figure in the main manuscript is supported by the supplemental data presented. Please justify how each of the supplemental figures meet the criteria.

-Sampling methods and the nature of "biological replicates" should be described precisely (i.e. different plants, parts of plants, pooled tissue, independent pools of tissue, sampled at different times, etc.), along with a clear description of and rationale for any statistical analyses conducted. The reader should know exactly what was sampled; what forms the basis of the calculation of any means and statistical parameters reported. This is also necessary to ensure that proper statistical analysis was conducted.

Responses to Review

We thank reviewers and editors for the constructive comments/suggestions on an earlier version of our manuscript (TPC2024-RA-00586R1). To address the review comments, we re-did the transactivation activity assay of MdH1.2, with MdH1.1 as a positive control, and revised the manuscript. The following are our responses to specific review comments (*Review comments in italics*).

Reviewer #1

The authors have addressed my queries/comments and the revised manuscript is much improved. There are some minor issues and the authors should address these issues in the final version.

line 253-254 "MdH1.1, MdWRKY53, and MdWRKY53L were highly co-expressed with MdMYB73 during fruit development in A4 and WT fruits (Figure 4B)": Is this correct? I think, MdH1.1 highly co-expressed only with MYB73.

Response: The original statement was incorrect. We re-worded it as "*MdH1.1* was highly co-expressed with *MdMYB73* whereas *MdWRKY53* was highly co-expressed with *MdWRKY53L* during fruit development in A4 and WT fruits"

line 369 electrophoretic mobility shift assays (EMSAs) by using a prokaryote-expressed....

Response: Corrected as suggested and checked the rest of the text as well.

Supplement Figures 1 and 2 legend: MdALMT9/Ma is annotated as a homolog (not analog) of Arabidopsis ALMT6 here.

Response: Corrected as suggested.

Reviewer #2

The authors have responded to all the questions raised, performed additional experiments, have improved figure quality (Figure 7) and included additional discussion points.

I have only a few minor suggestions:

Figure S3: Phylogenetic analysis of apple and Arabidopsis linker histone proteins.
Not all of these proteins are linker histones. They have been called GH1-domain proteins (PMID: 28298478). Some of them have already names e.g. GH1-HMGA1 or TRB that could be indicated.

Response: We modified the title of the figure (now Fig S4) to "Phylogenetic analysis of apple and Arabidopsis histone H1 globular (GH1)-domain proteins" and indicated those that have been named.

Figure S14: Why is the basal luciferase activity so much lower in the S15 (~ 100) and Figure 11,

compared to S14? Expression of MdH1.1 stimulates expression to over 500, but those levels are nearly already attained by the MdMYB73 vector alone in S14 questioning whether they could still be stimulated more in this experimental setting

Response: We re-did the dual Luc assay of MdH1.2, with MdH1.1 as a positive control. The data (now Fig S15) clearly show that MdH1.2 does not have transactivation activity on *MYB73* whereas MdH1.1 does. The significant background (basal) activity of *MYB73* promoter is likely related to the fact that two out of the three individual WHD binding sites in the promoter of *MYB73* have some background activity (Fig 7B and 7C). As the entire *MYB73* promoter is used here (Fig S15), the significant basal (background) activity is expected.

Reviewing Editor

Show individual data points for bar and line plots where the number of data points is less than 6 (e.g. Figs. 1B, 3, 5, 6B, 6D, 6F, 7L, 9C, 9E, 9F, 9G, 10A, 12A, 13, S6, S8B, S8D, S8F, S9).

Response: Done as suggested.

Show exact P-values for significance statement (e.g. in Figs. 4B, 8B, 9E, 10B, 11F)

Response: Done as suggested.

Remove statement "One asterisk means significant, and two asterisks mean extremely significant" from the legend of Fig. 4 and the asterisks from panel Fig. 4B.

Response: Removed the statement.

Change, if appropriate, "Tukey's Honest Significant Difference test" to "Tukey's Honestly Significant Difference test".

Response: Changed as suggested.

Specify in Fig. 7B whether gDNA or cDNA was used as template in the PCR reaction, and indicate the molecular size of the band in the gel.

Response: Clearly indicated gDNA and the size of the band.

Remove boxes around Figs. panels 8A, 8B, 10B.

Response: Done as suggested.

Editor-in-Chief

• *The color palette in many figures uses very similar tones (for example, blue, purple, and magenta in Figures 1, 3, 4, and 5, or pink/purple in others), which makes rapid visualization of the results challenging. I encourage you to adjust the palette to one with stronger tonal contrasts.*

Please also avoid, as much as possible, using green/red combinations to accommodate color-blind readers.

Response: We tested several color schemes, and settled on the current one that gives good contrast for rapid visualization of the results while avoiding green/red combinations as much as we can. We modified most of the figures in the paper accordingly.

• *The number of figures in the paper is quite large. While this isn't an issue in itself, extensive papers can discourage readers and may affect the visibility of your work. If possible, consider moving some data (such as control results) to supplementary materials or combining figures where feasible (for example, Figures 1 and 2; 3 and 4; 5 and 6; and 11, 12, and 13 can be merged while maintaining compliance with our guidelines).*

Response: We moved the original Fig 2 to Supplemental Information as Supplemental Fig S1, and combined the original Figs 5 and 6 into the new Fig 4, and the original Figs 12 and 13 into the new Fig 10. This led to a reduction of the total number of main figures from the original 14 to 11 now.

Dear Dr. Lailiang Cheng:

We are pleased to inform you that your paper entitled "A Linker Histone Acts as a Transcription Factor to Orchestrate Malic Acid Accumulation in Apple in Response to Sorbitol" has been accepted for publication in The Plant Cell, pending a final editorial review by a science editor. At this stage, your manuscript will be evaluated by a science editor with respect to its presentation of scientific content, compliance with journal policies, and presentation for a broad readership. The Plant Cell has contracted with Plant Editors (planteditors.com) to provide this service to our authors, and you will soon receive additional information on this process.

Please note that each author needs to link their ORCID identifier to their account in the system before your manuscript can be published. If any authors do not have an ORCID linked to their account, they will receive a message with a link to complete this task. Please ensure that ALL of your coauthors have completed this task as soon as possible.

LICENSE INFORMATION Shortly after your paper is sent to production, you will receive an email from our publisher's author support team, **SciPris**, with information on article processing charges, as well as the types of Open Access license you would be able to purchase. The accepted version of your manuscript will not appear on our Advance Articles page until the license is selected and signed, so please look out for an email from SciPris. For more information see the Article Publication Fees section of our author guidelines: https://academic.oup.com/plcell/pages/General-Instructions

The Plant Cell and The Arabidopsis Information Resource (TAIR) are collaborating to collect functional annotation data about Arabidopsis genes from authors. This includes information about the gene's molecular function (e.g., kinase activity, ATP synthetase activity), the biological process/es it is involved in (e.g., endosperm development, threonine biosynthesis), its subcellular location (e.g., nucleus, ER), anatomical or developmental expression pattern (e.g., leaf, ovule, flower stage 10, seedling stage), or its partner in a protein-protein interaction (e.g., AT1G01010 interacts with AT1G01020).

If your paper contains results falling into one or more of these categories for Arabidopsis genes, we request that you now submit these data for inclusion in TAIR by filling in the form provided at the following URL: https://www.arabidopsis.org/doc/submit/functional_annotation/123. If you need further clarification on what types of data can be submitted please contact curator@arabidopsis.org.

Finally, we encourage your submission of artwork for the journal cover. If there is an image or illustration related to your paper that you would like to have considered, please email it in .pdf or .tif format to clowe@aspb.org. An image title, brief, 2-3 sentence description of the image, and the name of the person credited for the image are also encouraged. If your image is selected to appear on the online cover of the journal, you will be contacted with the scheduled issue date and further information.

We look forward to seeing your paper published.

On behalf of the editorial board,

Sebastian Soyk, Board of Reviewing Editors
Robert Schmitz, Senior Editor
Pablo Manavella, incoming Editor-in-Chief

The Plant Cell

=====
IMPORTANT REMINDER: PEER REVIEW REPORTS
=====

If you opted to publish a peer review report along with your article during the original submission process, it will be prepared by the editorial staff and publicly posted with your manuscript, inside the zip file that contains any other supplemental material. As a reminder, the peer review report is a public record of all comments from editors and reviewers, as well as your prior responses, as you received them in the decision letters for each draft of your manuscript. If you agreed to publish this report and have changed your mind, or are not sure if you selected this option, please contact the editorial office as soon as possible before signing the license agreement from our publisher.

=====
REVIEWER COMMENTS
=====

----FOR ASPB OFFICE USE ONLY (DO NOT EDIT)----
MSID: 40112
Corresponding Author: Prof. Cheng
